# Constraining and Modifying Peptides Using Pd‐Mediated Cysteine Allylation

**DOI:** 10.1002/cbic.202300098

**Published:** 2023-05-04

**Authors:** Julia Kriegesmann, Thomas Schlatzer, Kateryna Che, Claudia Altdorf, Susanne Huhmann, Hanspeter Kählig, Dennis Kurzbach, Rolf Breinbauer, Christian F. W. Becker

**Affiliations:** ^1^ Institute of Biological Chemistry Faculty of Chemistry University of Vienna 1090 Vienna Austria; ^2^ Institute of Organic Chemistry Graz University of Technology 8010 Graz Austria; ^3^ Syntab Therapeutics GmbH Pauwelstrasse 17 post code? Aachen Germany; ^4^ Department of Organic Chemistry Faculty of Chemistry University of Vienna 1090 Vienna Austria

**Keywords:** allylation reaction, Diels-Alder reaction, peptide stapling, secondary peptide modification

## Abstract

Over the past decades, several strategies for inducing and stabilizing secondary structure formation in peptides have been developed to increase their proteolytic stability and their binding affinity to specific interaction partners. Here, we report how our recently introduced chemoselective Pd‐catalyzed cysteine allylation reaction can be extended to stapling and how the resulting alkene‐containing staples themselves can be further modified to introduce additional probes into such stabilized peptides. The latter is demonstrated by introducing a fluorophore as well as a PEG moiety into different stapled peptides using bioorthogonal thiol‐ene and Diels‐Alder reactions. Furthermore, we investigated structural implications of our allyl staples when used to replace conformationally relevant disulfide bridges. To this end, we chose a selective binder of integrin α_3_β_1_ (LXY3), which is only active in its cyclic disulfide form. We replaced the disulfide bridge by different stapling reagents in order to increase stability and binding affinity towards integrin α_3_β_1_.

## Graphical abstract

**Chemoselective Pd‐catalyzed cysteine allylation** is used for peptide stapling and the resulting alkene‐containing staples are further modified via biorthogonal reactions to introduce additional probes.

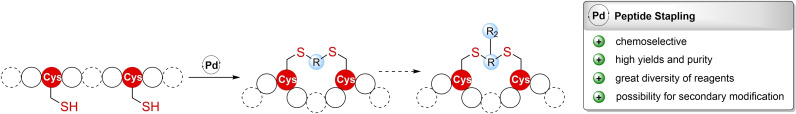

## Introduction

Protein‐protein interactions (PPIs) are involved in many different biological processes and therefore are highly attractive targets for drug development.^[1]^ PPIs cannot be easily targeted by small molecules as they lack defined binding pockets but instead have large contact areas.^[2]^ For this reason, peptides are often used to inhibit PPIs as they can target them more easily by mimicking the binding region of interaction partners.^[3]^ However, peptides lose their secondary structure when removed from their stabilizing protein context, which leads to reduced binding affinity towards the target protein. Furthermore, short unstructured peptides are rapidly degraded by proteases.^[4]^ Therefore, several strategies have been developed to stabilize the conformation of peptides to increase binding affinity towards specific proteins as well as proteolytic stability by cyclization.^[3]^

The main strategies for macrocyclization of peptides are (a) the linkage of two amino acid side chains, (b) the linkage of one side chain with the peptide‘s N‐ or C‐terminus, or (c) head‐to‐tail cyclization.[^3^, ^5^] Linking two side chains is mainly used for the stabilization of α‐helical peptides, a motif commonly found in PPIs. As an α‐helix contains 3.6 residues per turn, many strategies link side chains at the positions i/i+4, i/i+7, or i/i+11.^[3]^ The introduced constraints can either replace non‐interacting residues or they can be designed to replace interacting residues, by choosing a linker that can mimic this interaction.

Verdine and co‐workers introduced the term “stapled peptides” for peptides that are constrained in their α‐helical form by a hydrocarbon staple.^[6]^ Such staples are introduced *via* unnatural α‐methyl, α‐alkenyl amino acid building blocks in the peptide sequence that are macrocyclized by ruthenium‐catalyzed ring closing metathesis (RCM). These staples can either link one or two loops of the α‐helix, when introducing the building blocks at position i/i+4 or i/i+7, respectively. This strategy has been used to generate several bioactive peptides for different biological applications, such as targeting “undruggable” intracellular protein‐protein interactions.[^4^, ^7^, ^8^, ^9^] Besides this hydrocarbon stapling, other chemical strategies have been developed to staple peptides, including disulfide^[10]^ or lactam^[11]^ linkages as well as oxime,^[12]^ hydrazone,^[13]^ triazole[^14^, ^15^] and thioether bonds[^16^, ^17^] to link two side chains within the peptide sequence.

Recently, the staple itself was recognized as an element to introduce further diversity and/or to site‐selectively incorporate probes into the peptide. In this context, tetrazine chloride‐based reagents have been used for peptide stapling. Brown and Smith introduced the tetrazine linkage as an additional handle for further diversification of the peptides with a cyclooctyne carrying a fluorescein label by using an inverse‐electron demand Diels‐Alder reaction.^[18]^ Another approach is based on the modification of cysteine residues with alkyne‐containing dibromomaleimides. The alkyne moiety allows further functionalization with reagents such as biotin, fluorescein and PEG carrying an azide functionality.^[19]^

We previously reported a method for peptide stapling through the reaction of two cysteine side chains with bifunctional reagents in the presence of a Pd/BIPHEPHOS catalyst system.[^20^, ^21^] Now we extend this approach to a small library of staples with varying length and rigidity, analyze their ability to mimic disulfide bonds and explore two chemoselective reactions for further functionalization of these staples.

## Results and Discussion

**Figure 1:**
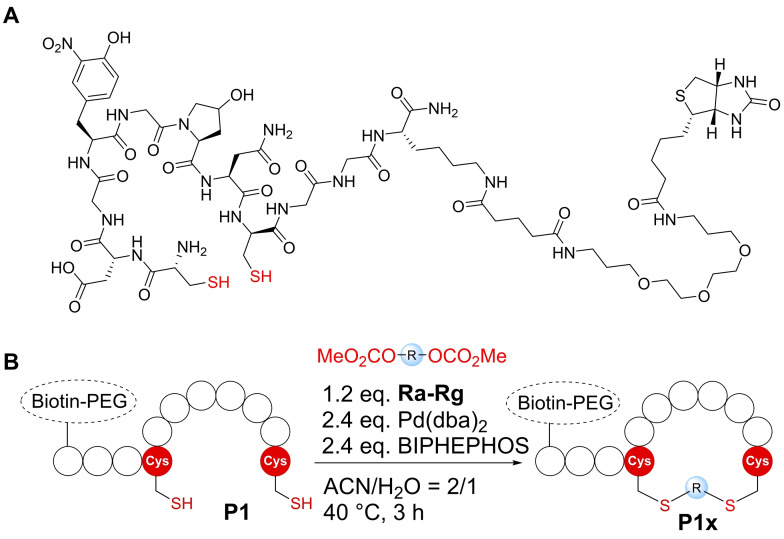
A: Structure of peptide **P1**. Thiol groups shown in red form a disulfide bond to give the active binder (**P2**). B: Cyclization reactions of **P1** under standard conditions with different stapling reagents.

We apply this approach on a relevant and well‐described selective binder peptide of integrin α_3_β_1_, namely LXY3 (sequence: cdGY(3‐NO_2_)GP(OH)Nc). This peptide cyclizes through a disulfide bond between the two terminal D‐cysteine residues.^[22]^ As the peptide only binds the integrin in its cyclic form, we wanted to substitute its disulfide bridge by different staples to chemically stabilize the cyclic peptide and to explore the influence of different linker conformations and lengths on the binding affinity of the peptide towards integrin α_3_β_1_. Therefore, we introduced a C‐terminal biotin tag into the peptide (**P1**, sequence: cdGY(3‐NO_2_)GP(OH)NcGGK(PEG‐Biotin)‐NH_2_, Figure 1) that allows direct readout of binding in flow cytometry experiments *via* streptavidin‐PerCP‐Cy5.5 staining integrin α_3_β_1_ on cell surfaces. The GGK motif as well as the PEG spacer between peptide and biotin were included to avoid interference between binding events to the integrin as well as to labeled streptavidin.

We synthesized seven different bifunctional stapling reagents (**Ra**–**Rg**) with allylic methyl carbonate groups varying in length, hydrophobicity as well as rigidity (Table 1) and reacted them with peptide **P1**. This selection of staples allowed us to assess if different conformations of the staple as well as its length and hydrophobicity contribute to binding. High conversions were observed in all cases (95–100 %, see ESI for exemplary HPLC traces) except for the alkyne‐based reagent **Rd**. Stapled variants **P1 a**–**c** and **P1 e**–**g** were obtained in high yields and purity (Table 1). For reaction 4 with staple **Rd**, the propargylic nature of the staple (instead of the otherwise used allylic staples) reduced the conversion to the desired product (∼50 % in 6 h). Based on similar observations with non‐peptidic thiols (see ESI Figure S1) we concluded that allylic staples react faster and give higher conversion.

**Table 1:** Structures of stapling reagents as well as isolated yields and purity of all stapled P1 variants.

Entry	Product	Stapling reagent	Conv.^[a]^	Isolated yield
1	**P1 a**	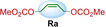	>95 %	1.1 mg, 32 %
2	**P1 b**	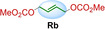	Full conv.	0.7 mg, 19 %
3	**P1 c**	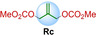	Full conv.	1.0 mg, 30 %
4	**P1 d**	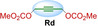	∼50 %	0.3 mg, 9 %
5	**P1 e**	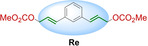	>95 %	1.0 mg, 29 %
6	**P1 f**	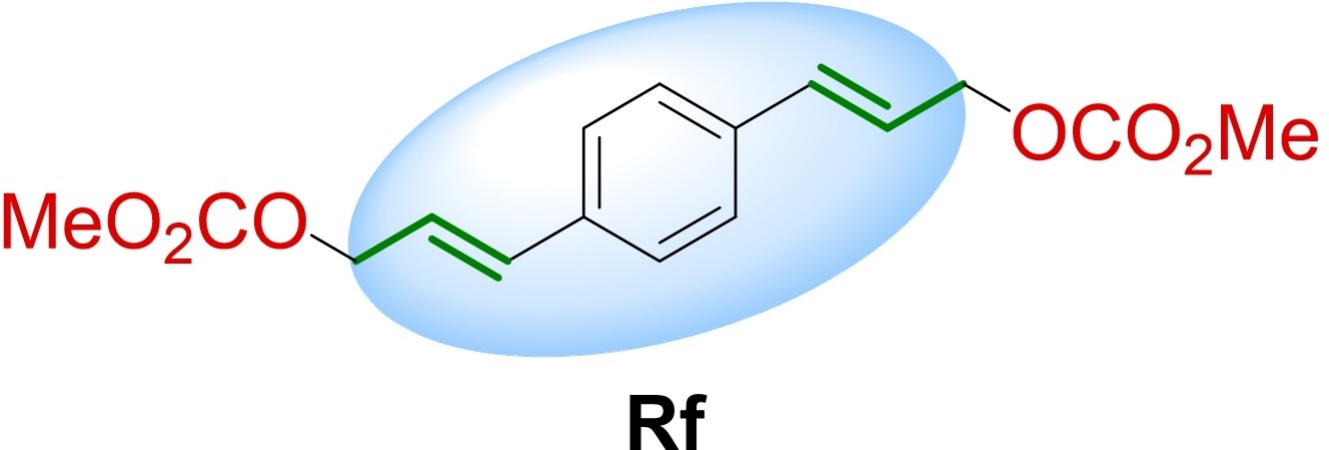	Full conv.	1.3 mg, 36 %
7	**P1 g**	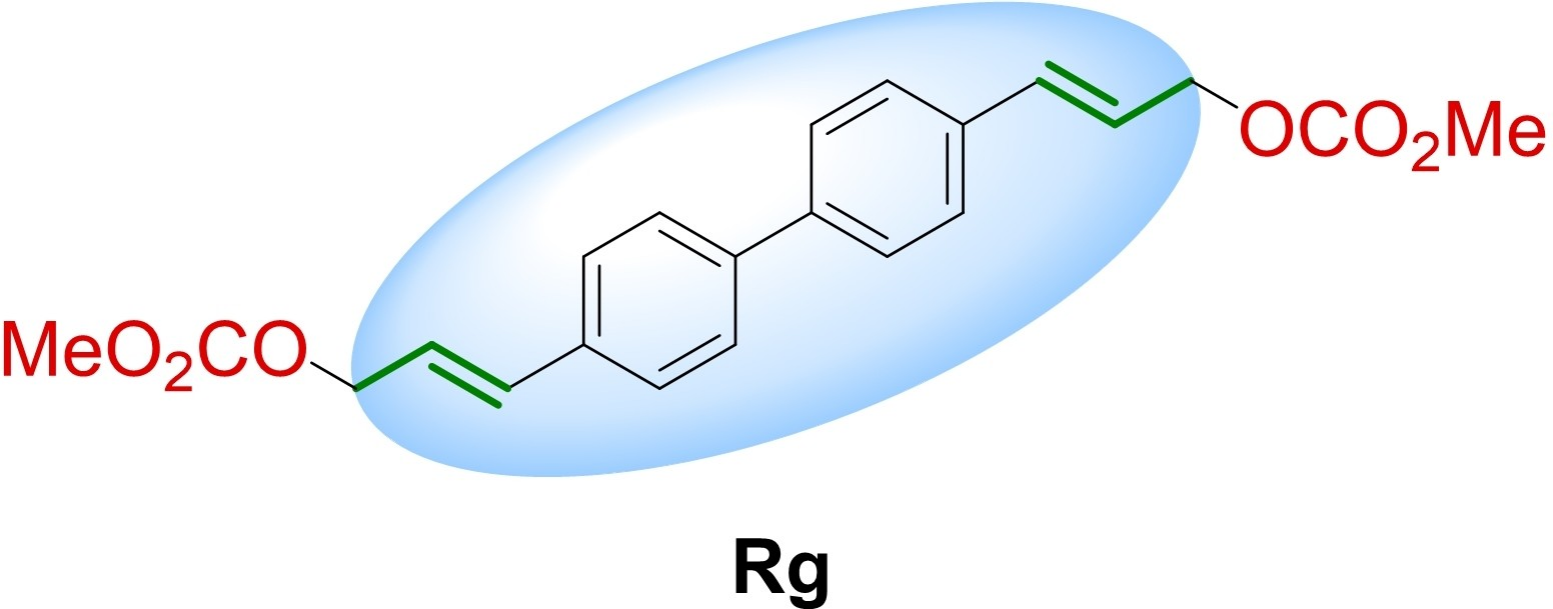	>95 %	1.7 mg, 45 %

[a] Conversion has been determined based on HPLC analysis of the consumed peptide.

The obtained stapled peptides **P1 a**–**g** were tested for binding to integrin α_3_β_1_ expressed on human MDA‐MB231 cells (Figure S2). Upon incubation for 30 min with different concentrations of the stapled **P1 a**–**g** variants, streptavidin‐PerCP‐Cy5.5 was added after three washing steps to allow detection by flow cytometry. Peptide **P2** (**P1** in its disulfide cyclized form) served as a positive control, which bound with an apparent affinity of 100 nM.^[23]^

Interestingly, the olefin‐isomeric peptides **P1 a** and **P1 b** showed very different affinity. While **P1 a** resulted in a binding affinity of ∼10 μM, which is two orders of magnitude lower than for the positive control **P2**, **P1 b** did not show any measurable binding in our assays, although the only difference between the two peptide variants is the *Z*‐ and *E*‐ configuration of the staple. To investigate the impact of staples on peptide conformation in more detail, we performed NMR measurements of the stapled peptide variants as well as MD (molecular dynamics) simulations.

**Figure 2:**
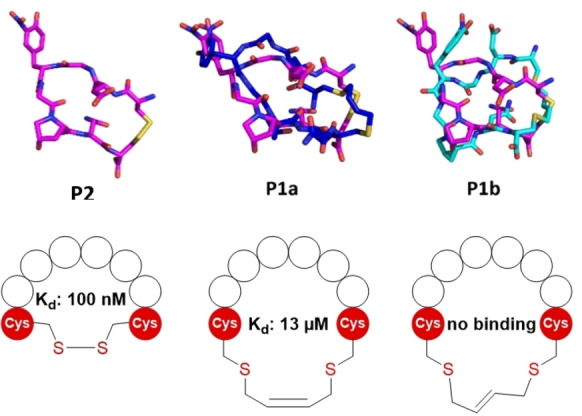
Simulated structures of the peptides **P2** (magenta) as well as **P1 a** (blue) and **P1 b** (cyan) overlayed with **P2** (magenta), obtained from the MD trajectories. The shown snap shots are taken after 800 ns of equilibration, *i. e*., at a time point when the RMSD of the trajectories remained constant (see ESI). Hence, they can be considered representative of an energy‐minimized structure, although it remains unclear whether the structure represents the global energy minimum.

Figure 2 shows structures obtained from MD simulations after 800 ns of peptides **P1 a** and **P1 b** (overlayed with the structure of **P2**) supporting, together with NMR measurements (see ESI Figure S5), the fact that different peptide conformations are induced by the *cis* and *trans*‐configuration of the staples. Such subtle changes in the linker can translate into deviations in ring conformations, resulting in severe differences in binding. While **P1 a** and **P2** roughly resemble each other, **P1 b** adopts a more extended conformation. These findings agree with the recorded NMR spectra (see ESI Figure S5). As expected, the strongest chemical shift differences were observed for the two cysteine residues upon changing the staple configuration. In addition, residue N_7_ showed a significant change in chemical shift between **P1 a** and **P1 b**. The chemical shifts of all other resonances remain largely unchanged, except for the side chain resonances. These observations also align well with the MD‐derived structures. In Fig. 2, it can be observed that the side chain of residue N_7_ is pointing towards the inner space of the peptide ring for **P1 b** (likely due to the open ring configuration), but not for **P1 a**. Peptide **P1 d** exhibited similar binding as determined for **P1 a** (∼10 μM). This data indicates that the **P2** conformation, which allows efficient binding to integrin α_3_β_1_, is easily disturbed by extended staple structures and that only small linkers are acceptable replacements of the original disulfide bond, leading to affinities in the low μM range. (We confirmed the binding affinity of the positive control and **P1 a**–**c** on mouse 4T1 cells as well (see ESI Figure S2).) In contrast, larger, extended stapling reagents such as **Rf**–**Rg** cannot stabilize a more strained ring conformation, and therefore, the resulting cyclic peptides show no detectable binding to integrin α_3_β_1_. Direct interference of the larger staples by blocking binding to the integrin can also not be excluded here. Overall, we provide a set of easily accessible staples that cover a large range of distances and variable rigidity allowing their flexible application in different settings. In case of peptide **P1**, only the initially selected disulfide bridge provides the ideal binder conformation, however this should be different in many other cases, e. g. when used to stabilize helices.

**Figure 3:**
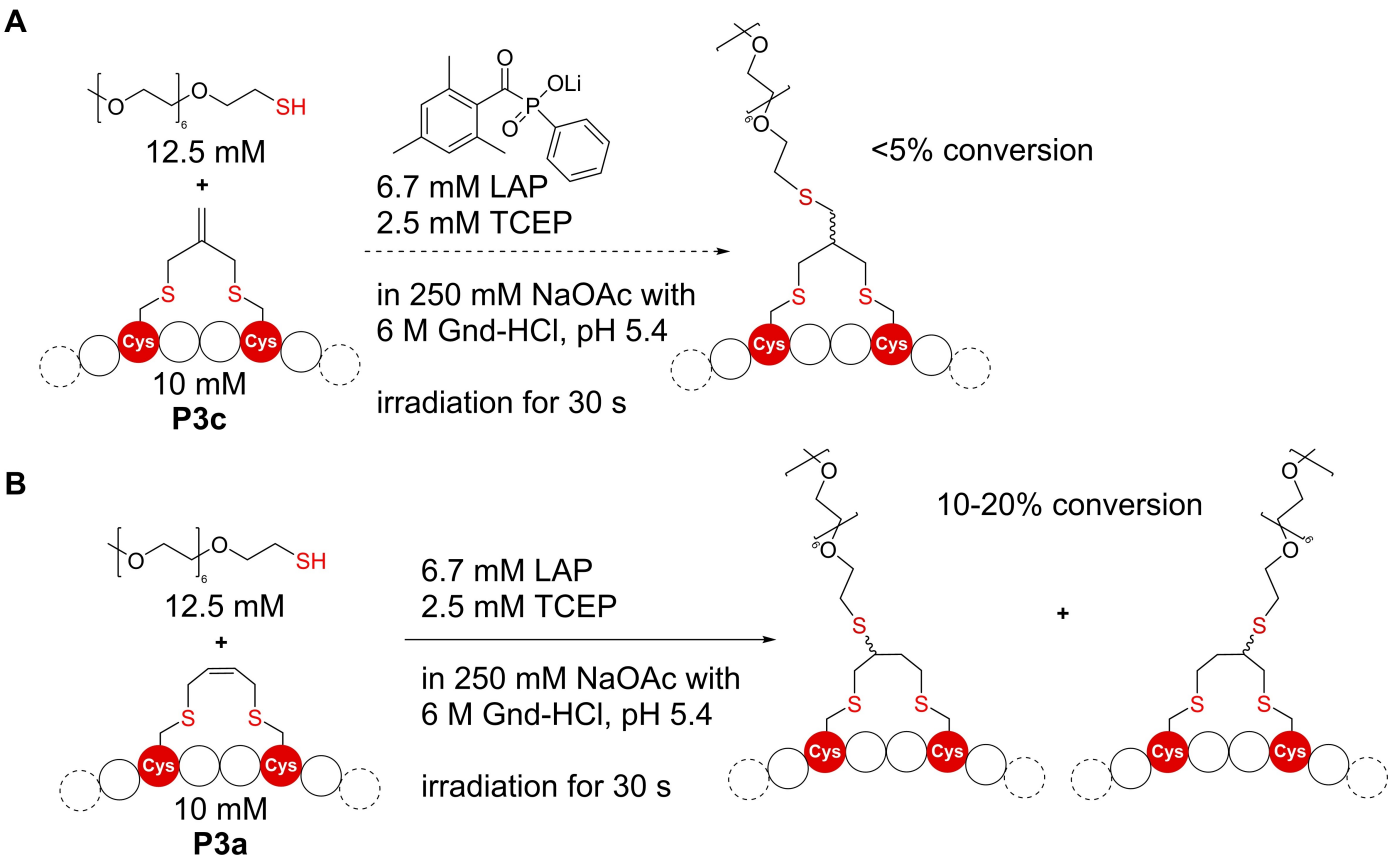
Thiol‐ene reaction of stapled peptides using LAP as a radical initiator. During the modification of a staple containing a terminal double bond (A) two products can be isolated, which are most likely epimers of the terminal addition product. The reaction with an internal double bond (B) opens the way to four different products, which were not resolved.

After generating this series of seven stapled **P1** peptides applying bifunctional allyl/propargyl carbonates, we continued by taking advantage of further modification opportunities offered by these staples. The one or two double bonds in each staple lend themselves to secondary modification reactions, thereby allowing the installation of different functionalities such as fluorophores or affinity tags onto the stabilized peptide. We tested thiolene reactions with peptides **P3 a** and **P3 c** (Figure 3, peptide **3**: EWACTACAKFLAAHA, which has been used in stapling reactions before^[24]^). Stapled peptides **P3 a** and **P3 c**, containing one double bond, were reacted with short, homogeneous, thiol‐functionalized oligoethylenes in the presence of LAP (lithium phenyl‐2,4,6‐trimethylbenzoylphosphinate) as a photoinitiator (Figure 3). We tested different reaction conditions, using published protocols^[25]^ as a starting point for further optimization. During the reaction, degassed buffers were used in combination with 2.5 mM TCEP and the experiments were performed under a gentle stream of argon to prevent disulfide formation. At higher TCEP concentrations, desulfurization of cysteines was observed, similar to established desulfurization strategies for peptides and proteins based on TCEP in combination with radical initiators such as VA‐044.^[26]^ For **P3 c** containing a terminal double bond (Figure 3A), we observed two product peaks with identical molar mass during LC–MS analysis and successfully isolated the two products (see ESI Figure S9). Since the *anti*‐Markovnikov product is highly favored for the addition of thiols to unsymmetrically substituted double bonds (due to the preferred formation of the more stable C‐radical intermediate),[^27^, ^28^] we concluded that we can separate both epimers here. For a similar reaction with **P3a** containing a double bond in the staple (Figure 3B), we only observed traces of product, which most likely also contain a mix of different products. This is in accordance with literature, as additions to higher substituted molecules are generally slower than additions to terminal double bonds.^[27]^ As the conversion was too low in all attempts (10–20 % for **P3 c** and only traces for **P3 a**) to be useful for efficient labeling of peptides, we did not further investigate this reaction.

**Figure 4:**
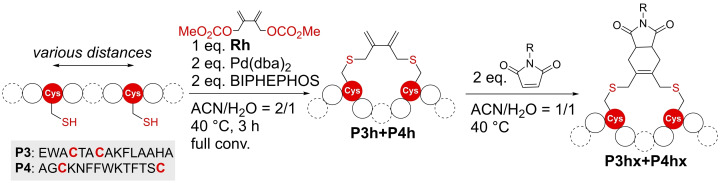
Peptide stapling of **P3** and **P4** with **Rh** followed by Diels‐Alder reaction with different dienophiles. During some of the reactions two peaks with the desired mass were observed during LC–MS analysis.

Alternatively, we explored Diels‐Alder reactions with peptides **P3** and **P4** (sequence: AGCKNFFWKTFTSC, another peptide that has been use in stapling reactions before^[18]^) stapled with a novel bifunctional allyl carbonate **Rh** (Figure 4). Diels‐Alder reactions have been proven to be orthogonal when used in the context of unprotected peptides and proteins.^[29]^ Stapled peptides **P3 h** and **P4 h** differ drastically in the number of amino acids in the macrocycle formed during stapling (2 vs. 10 aa) and further demonstrate the scope of use for our staples. Both peptides were reacted first with the electron poor dienophile 4‐phenyl‐1,2,4‐triazoline‐3,5‐dione (PTAD), leading to the desired products in only 5 min. Then, we investigated the reaction with less reactive but easily available, functionalized dienophiles, such as the maleimide derivatives 6‐maleimido hexanoic acid (6‐MHA), 3‐maleimido propionic acid (3‐MPA) as well as a maleimide containing a fluorescent label. Although the reactions were much slower, full conversion was achieved within 48–72 h when using a peptide concentration of 5 mM and 2 eq. of dienophiles. This clearly demonstrated that Diels‐Alder reactions can be used for quantitative secondary modification with PTAD as well as maleimide derivatives on a set of suitable staples. As a great variety of these dienophiles is commercially available and allows secondary modification of stapled peptides for many different applications.

LC–MS/MS measurement confirmed that the expected Diels‐Alder product was formed and no side reaction with the lysine side chain occurred (see ESI Figure S8). Interestingly, in some cases two peaks with identical mass were observed during LC–MS analysis, similar to the epimers found with **P3 c** (Table 2).

**Table 2:** Diels‐Alder reactions of 5 mM **P3 h** or **P4 h** with 2 eq. of different dienophiles. Reaction times until full conversion was achieved as well as the number of products are shown.

Entry	Compound	Reaction time until full conversion		Number of products	
		**P3 h**	**P4 h**	**P3 h**	**P4 h**
1	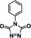	5 min	5 min	1	2
2	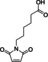	48 h	48 h	1	2
3	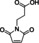	48 h	72 h	2	2
4	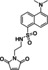	48 h	72 h	2	1

**Figure 5:**
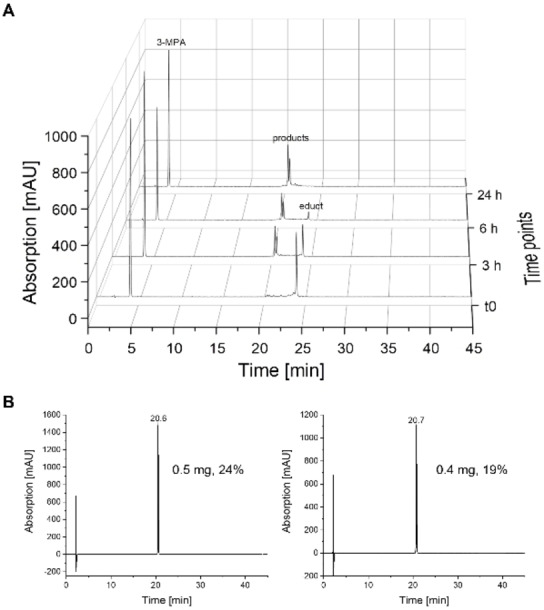
Diels‐Alder reaction of **P3 h** with 3‐MPA. A: HPLC measurements at different time points. The reaction was finished after 24 h and two products with the same mass were formed. B: HPLC analysis of the two isolated products showed that they have different retention times.

To further investigate the cause for these two products, we repeated the reaction of **P3 h** with the 3‐MPA on a larger scale. Increasing the reaction temperature from RT to 40 °C reduced the reaction time from 48 to 24 h (Figure 5). The two obtained products were isolated by RP‐HPLC (yield: 0.5 mg, 24 % and 0.4 mg, 19 %) and analyzed by NMR. The measured NMR spectra are very similar for both products. This can be seen by an overlay of the two individual ^1^H‐^13^C HSQC spectra in Figures S6/S7. The N and C terminal parts of the peptides match completely. Minor deviations appear for the α and β signals of the amino acids within the staple and directly adjacent to the two cysteines in the chain. More pronounced deviations are observable for the signals at the tetrahydroisoindole‐1,3‐dione formed during DA reactions. Typically, only one DA product (*endo*) is favored, but here the stapled peptide **P3 h** has the same substituents on the outer and inner side of the diene, namely protons. Such a system can lead to the formation of both *endo* and *exo*‐like transition states and in turn to DA products in which the dienophile part points to different sides of the peptide. Such two isomers could be formed in all DA reactions with stapled peptides **P3 h** and **P4 h** with the different dienophiles (Table 2), but as the retention times on the RP‐HPLC are very similar, they are not always resolved. Based on the fact that the *endo/exo*‐like orientation occurs within the staple and based on our NMR data (Figure S6/S7) showing a non‐altered peptide conformation, we do not expect detrimental effects on peptide properties.

## Conclusion

The efficient stapling of three different peptides with a series of eight different stapling reagents establishes this Pd‐catalyzed reaction as a versatile and robust alternative to other stapling strategies discussed above. New data on the impact of minor differences in staples (e. g. *cis/trans* orientation of the double bond) reveals the need for easily exchangeable stapling options provided by the allylic methyl carbonate staples used here. Bifunctional allyl carbonate staples can be efficiently incorporated into peptides containing two cysteine residues and typically lead to constrained peptides in high yields and purity without any recognizable limits with respect to ring size and the use of non‐canonical amino acid building blocks. Therefore, a set of stapled peptides varying in size and conformational flexibility, as shown for **P1**, can be generated from one precursor peptide. This strategy has the additional advantage over methods, such as the ones based on allylic halides, that our reagents are highly selective for cysteine and are bench stable. Secondary modification of the staples has been explored here as well, and even though thiolene reactions are possible, they did not lead to satisfactory yields. Alternatively, diene‐containing staples provide the basis for high‐yielding Diels‐Alder reactions. Such staples pave the way for further diversifying the stapled peptides and allow the addition of variable probes. The latter are often commercially available as maleimide reagents and can help to quickly increase the diversity of available peptides and to switch probes such as fluorophores or affinity tags.

## Conflict of interest

The authors declare no conflict of interest.

1

## Supporting information

As a service to our authors and readers, this journal provides supporting information supplied by the authors. Such materials are peer reviewed and may be re‐organized for online delivery, but are not copy‐edited or typeset. Technical support issues arising from supporting information (other than missing files) should be addressed to the authors.

Supporting Information

## Data Availability

The data that support the findings of this study are available from the corresponding author upon reasonable request.
