# Supplementary material for: Constraining and Modifying Peptides Using Pd‐Mediated Cysteine Allylation
Source: Chembiochem. 2023 May 4;24(13):e202300098. doi: 10.1002/cbic.202300098 (PMC10947015; doi:10.1002/cbic.202300098)
Supplement: Supplementary file 1 — Supporting Information [file CBIC-24-0-s001.pdf]

ChemBioChem

Supporting Information

Constraining and Modifying Peptides Using Pd-Mediated Cysteine Allylation

Julia Kriegesmann, Thomas Schlatzer, Kateryna Che, Claudia Altdorf, Susanne Huhmann, Hanspeter Kählig, Dennis Kurzbach, Rolf Breinbauer, and Christian F. W. Becker*

Table of Content

General information 2
Intrinsic reactivity of propargylic and allylic stapling reagents 5
Binding of stapled peptides to murine 4T1 cells and human MDA-MB231 cells 6
MD simulations of P1 variants 8
NMR measurements of peptides P1a and P1b 9
NMR measurements of the two Diels-Alder products 11
LC-MS/MS measurements to confirm correct Diels-Alder product 14
Peptide Stapling 15
Thiol-ene reactions 16
Diels-Alder reactions 17
Synthesis of allylic carbonates 18
Synthesis of propargylic and allylic stapling reagents 24
NMR-Spectra 26
Analysis of the peptides 41
References 48

General information

Peptide Synthesis

All commercially available chemicals and solvents were purchased from Acros Organics, Alfa Aesar, Fisher, Fluka, Honeywell, Merck, Roth, Sigma-Aldrich, TCI, VWR and used without further purification, unless otherwise stated. Solvents for peptide synthesis and chromatography were of "peptide synthesis grade" and "HPLC grade", respectively. Protected Fmoc-amino acids, resins and coupling reagents were purchased from Novabiochem and Iris.

The solid-phase peptide synthesis, purification and analysis as well as allylation of peptides were performed based on our published protocols.[1]

Chemical Synthesis

General aspects. If reactions were performed under inert conditions, e.g. exclusion of water, oxygen or both, all experiments were carried out using established Schlenk techniques or inside a nitrogen-filled glovebox (MBraun UNIlab pro). Solvents were dried with common methods and afterwards stored under inert gas atmosphere (argon) over molecular sieves. In some cases, when explicitly mentioned, dry solvents were received from the mentioned suppliers. In general, when high vacuum (*in vacuo*) was stated in experimental procedures, typically a vacuum of 10^{-2}-10^{-3} mbar was applied. Degassing of solvents or reaction mixtures was performed via three freeze-pump-thaw cycles. All reagents were added in a counterstream of inert gas to keep the inert atmosphere. All reactions were stirred with Teflon-coated magnetic stirring bars. Molecular sieves (3 Å or 4 Å) were activated in a round-bottom flask with a gas inlet adapter by heating them carefully in a heating mantle at level 1 at least for 24 h under high vacuum until complete dryness was obtained. These activated molecular sieves were stored at r.t. under argon atmosphere. Temperatures were measured externally if not otherwise stated. When working at a temperature of 0 °C, an ice-water bath served as the cooling medium. Lower temperatures were achieved by using an acetone/dry ice cooling bath. Reactions, which were carried out at higher temperatures than r.t., were heated in a silicon oil bath on a heating plate (RCT basic IKAMAG® safety control, 0-1500 rpm) equipped with an external temperature controller.

Chemicals. All commercially available chemicals and solvents were purchased from abcr, Acros Organics, Alfa Aesar, Fisher, Fluka, Honeywell, Merck, Roth, Sigma Aldrich, TCI, VWR and used without further purification, unless otherwise stated.

Anhydrous Solvents. Anhydrous acetonitrile was purchased from Alfa Aesar in >99.8% purity. It was transferred into an amber 1 L Schlenk bottle and stored over activated 3 Å molecular sieves under argon atmosphere. Dichloromethane (stabilized with EtOH) was purchased from Fisher, dried over phosphorus pentoxide, distilled and heated under reflux over CaH_2 for 24 h. It was distilled into an amber 1 L Schlenk bottle and stored over 4 Å molecular sieves under argon atmosphere. Anhydrous *N*,*N*-dimethylformamide was purchased from Sigma

Aldrich in 99.8% purity. It was transferred into an amber 1 L Schlenk bottle and stored over activated 4 Å molecular sieves under argon atmosphere. Tetrahydrofuran was purchased from VWR and heated under reflux over Na until benzophenone indicated dryness (intense blue color). It was distilled into an amber 1 L Schlenk bottle and stored over 4 Å molecular sieves under argon atmosphere. Toluene was purchased from Fisher and dried through an aluminium oxide column under inert conditions. It was filled into an amber 1 L Schlenk bottle and stored over activated 4 Å molecular sieves under argon atmosphere.

Thin layer chromatography. Analytical thin layer chromatography (TLC) was carried out on Merck TLC silica gel aluminum sheets (silica gel 60, F254, 20 x 20 cm). All separated compounds were visualized by UV light (λ = 254 nm and/or λ = 366 nm) and by $KMnO_4$ staining reagent (3.0 g $KMnO_4$ and 20 g K_2CO_3 were dissolved in 300 mL H_2O and afterwards 5.0 mL 5% aq. NaOH were added) followed by development in heat.

Flash column chromatography. Flash column chromatography was performed on silica gel 60 from Acros Organics with particle sizes between 35 µm and 70 µm. Depending on the problem of separation, a 30 to 100-fold excess of silica gel was used with respect to the dry amount of crude material. The dimension of the column was adjusted to the required amount of silica gel and formed a pad between 10 cm and 30 cm. In general, the silica gel was mixed with the eluent and the column was equilibrated. Subsequently, the crude material was dissolved in the eluent and loaded onto the top of the silica gel and the mobile phase was forced through the column using a rubber bulb pump. The volume of each collected fraction was adjusted between 20% and 40% of the silica gel volume.

Gas Chromatography. GC-MS analyses were performed on an Agilent Technologies 7890A GC system equipped with a 5975C mass selective detector (inert MSD with Triple Axis Detector system) by electron-impact ionization (EI) with a potential of E = 70 eV. Herein, the samples were separated depending on their boiling point and polarity. The desired crude materials or pure compounds were dissolved, and the solutions were injected by employing the autosampler 7683B in a split mode 1/20 (inlet temperature: 280 °C; injection volume: 0.2 µL). Separations were carried out on an Agilent Technologies J&W GC HP-5MS capillary column ((5%-phenyl)methylpolysiloxane, 30 m x 0.2 mm x 0.25 µm) with a constant helium flow rate (He 5.0 (Air Liquide), 1.085 mL·min^{-1}, average velocity: 41.6 cm·s^{-1}). A general gradient temperature method (50S) was used: initial temperature: 50 °C for 1 min; linear increase to 300 °C (40 °C·min^{-1}); hold for 5 min; 1 min post-run at 300 °C; detecting range: 50.0-550.0 amu; solvent delay: 2.60 min.

High Performance Liquid Chromatography. Analytical HPLC-MS measurements were performed on an Agilent Technologies 1200 Series system (G1379 Degasser, G1312 Binary Pump, G1367C HiP ALS SL Autosampler, G1330B FC/ALS Thermostat, G1316B TCC SL column compartment, G1365C MWD SL multiple wavelength detector (deuterium lamp, 190-400 nm)) equipped with a single quadrupole LCMS detector "6120 LC/MS" using electrospray ionization source (ESI in positive and negative mode). Separations were carried out on a reversed phase Agilent Poroshell 120 EC-C18 (100 x 3.0 mm, 2.7 µm) column equipped with a Merck LiChroCART® 4-4 pre-column. The following method (2-100-EC-C18) was used: 0.0 min: 98% H_2O (0.05% TFA) and 2% CH_3CN; 0.0-6.0

min: linear gradient to 100% CH_3CN; 6.0-8.0 min: 100% CH_3CN; 8.0-8.5 min: linear gradient to 98% H_2O (0.05% TFA) and 2% CH_3CN; 8.5-9.5 min: 98% H_2O (0.05% TFA) and 2% CH_3CN; 0.700 mL·min^{-1}; 35 °C.

Nuclear Magnetic Resonance Spectroscopy. NMR spectra were recorded on a Bruker Avance III 300 spectrometer (^{1}H: 300.36 MHz; ^{13}C: 75.53 MHz) with autosampler. Chemical shifts δ are referenced to the residual proton and carbon signal of the deuterated solvent ($CDCl_3$: δ = 7.26 ppm (^{1}H), 77.16 ppm (^{13}C)).[2,3] Chemical shifts δ are given in ppm (parts per million) and coupling constants *J* in Hz (Hertz). If necessary, 1D spectra (APT and NOESY) as well as 2D spectra (H,H-COSY, HSQC, HMBC) were recorded for the identification and confirmation of the structure. Signal multiplicities are abbreviated as s (singlet), br s (broad singlet), d (doublet), t (triplet), q (quartet), quint (quintet), m (multiplet), dd (doublet of doublets), td (triplet of doublets), dt (doublet of triplets), and qd (quartet of doublets). Deuterated solvents for nuclear resonance spectroscopy were purchased from euriso-top®.

High Resolution Mass Spectrometry. High-resolution mass spectra (DI/GC-EI-TOF) were recorded on a Waters Micromass GCT Premier system. Ionization was realized by an electron impact source (EI ionization) at a constant potential of 70 eV. Herein, individual samples were either inserted directly (direct inlet electron impact ionization) or prior to this gas chromatographically separated on an Agilent 7890A system equipped with an Agilent Technologies J&W GC-column DB-5MS (length: 30 m; inner-diameter: 0.250 mm; film: 0.25 µm) at a constant helium flow. Molecule ions were analyzed by a time-of-flight (TOF) mass analyzer in the positive mode (TOF MS EI+). Further high-resolution mass spectra (LC-ESI-MS/MS) were acquired by data-dependent high-resolution tandem mass spectrometry on a QExactive Focus (Thermo Fisher Scientific, Germany). The electrospray ionization potential was set to +3.5 or -3.0 kV, the sheath gas flow was set to 20, and an auxiliary gas flow of 5 was used. Samples were diluted with an appropriate solvent (methanol or chloroform) and 1 µL was injected on a SeQuant® ZIC®-pHILIC HPLC column (Merck, 100 x 2.1 mm; 5 µm; 100 Å; peek coated; equipped with a guard column) or on a RP-column (Waters, ACQUITY UPLC HSS T3 150 x 2.1 mm; 1.8 µm with VanGuard column). The separation solvent (pHILIC: A: CH_3CN, B: 25 mM NH_4HCO_3; RP: A: 0.1% HCOOH, B: 0.1% HCOOH in CH_3CN) was delivered through an Ultimate 3000 HPLC system (Thermo Fisher Scientific, Germany) with a flow rate of 100 µL·min^{-1} and appropriate gradients were used for proper sample elution.

Determination of Melting Points. Melting points were determined on a Mel-Temp® melting point apparatus from Electrothermal with an integrated microscopical support. They were measured in open capillary tubes with a mercury-in-glass thermometer and were not corrected.

Intrinsic reactivity of propargylic and allylic stapling reagents

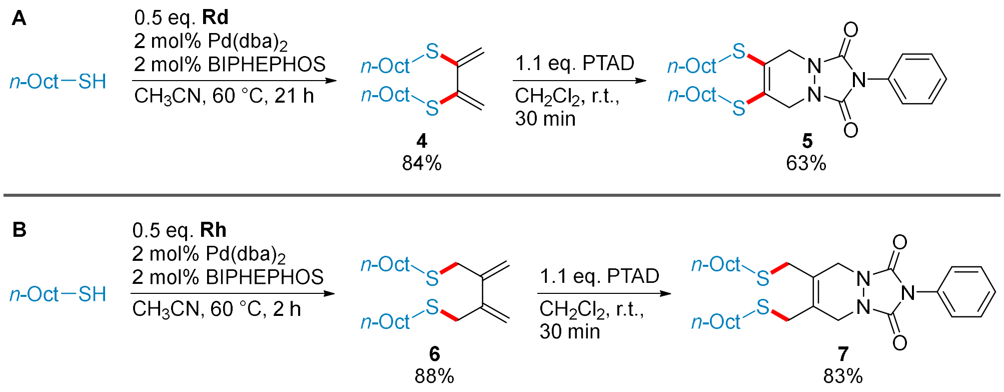

Figure S1: Comparison of propargylic (**A**) and allylic (**B**) carbonate reagents for the installation of 1,3-butadiene motifs and their subsequent Diels-Alder reaction.

Binding of stapled peptides to murine 4T1 cells and human MDA-MB231 cells

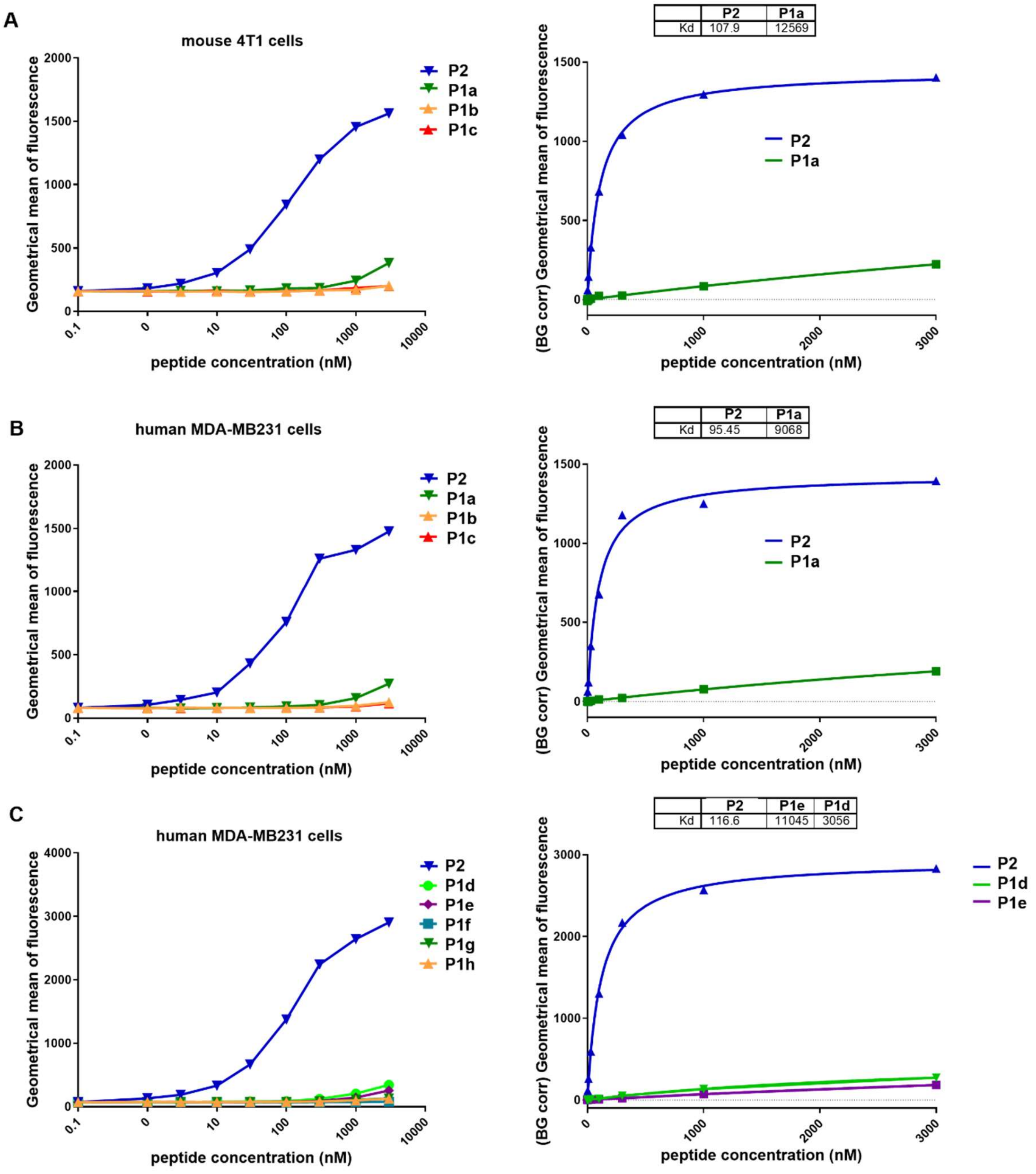

Figure S2: Determination of K_d values for peptides **P1a**-**h** based on streptavidin-PerCP-Cy5.5 staining of integrin-containing cells incubated with different peptide concentrations. A: Peptides **P1a**-**c** in mouse 4T1 cells. B: **P1a**-**c** in human MDA-MB231 cells. C: Peptides **P1d**-**h** in human MDA-MB231 cells.

4T1 and MDA-MB231 cells were grown in culture according to the suppliers' guidelines, and cells were detached by using trypsin/EDTA (PAN, diluted 1:2 with PBS). Resuspended cells in complete culture medium were centrifuged and washed once with washing buffer [HEPES (pH 7.3, 10 mM) in NaCl (0.9 %)] at 4°C and resuspended ($2x10^6$ cells mL^{-1}) in peptide blocking buffer (PBB): HEPES (pH 7.3, 10 mM) in NaCl (0.9%) + KCl (5 mM), BSA (3%) + FCS (5 %) + $MnCl_2$ (4 mM). Peptide samples were prepared at twice the final concentration in PBB and added to the cells to give final peptide concentrations of 1 nM - 3 µM. Cells ($1x10^5$ per 50 µL) were aliquoted into FACS tubes and incubated for 10 min on ice before addition of the peptide samples (50 µL). After 30 min incubation on ice, cells were washed three times with washing buffer and resuspended in streptavidin-

PerCP-Cy5.5 (100 µL, Beckton Dickinson, 0.5 mg mL^{-1}) in PBB. After incubation on ice for 15 min, PBS (1 mL) was added, and cells were pelleted and resuspended in PBS (100 µL) 0°C/Hoechst (0.5 mg mL^{-1}). Cells were kept on ice until analysis with a FACS Canto instrument and 10000 events/sample were recorded. Data were evaluated with Flowing software. Gating was performed on single and live cells, and histograms of PerCP–Cy5.5 fluorescence were plotted. K_d values were calculated by fitting the data to a one-site specific binding model [$Y=B_{max}VX/(K_d+X)$] by using GraphPad Prism, and errors represent the standard error of the fit.

MD simulations of P1 variants

All-atoms MD simulations were carried out using the YASARA software package.[6,7] The peptides were created together with the biotin tag directly in YASARA. Energy minimization was performed before each MD run for each peptide. All simulations were performed at 37°C using explicit solvent (1% NaCl in H_2O at pH 7.4). Trajectories were run using the AMBER14[8] force field with periodic boundary conditions and 1.25 fs time steps. The total length of the MD trajectory for each peptide was 1 µs. Each MD simulation was repeated 3 times. The simulation box had a size of 37 Å side length (cubic). The pressure was set to 1 bar.

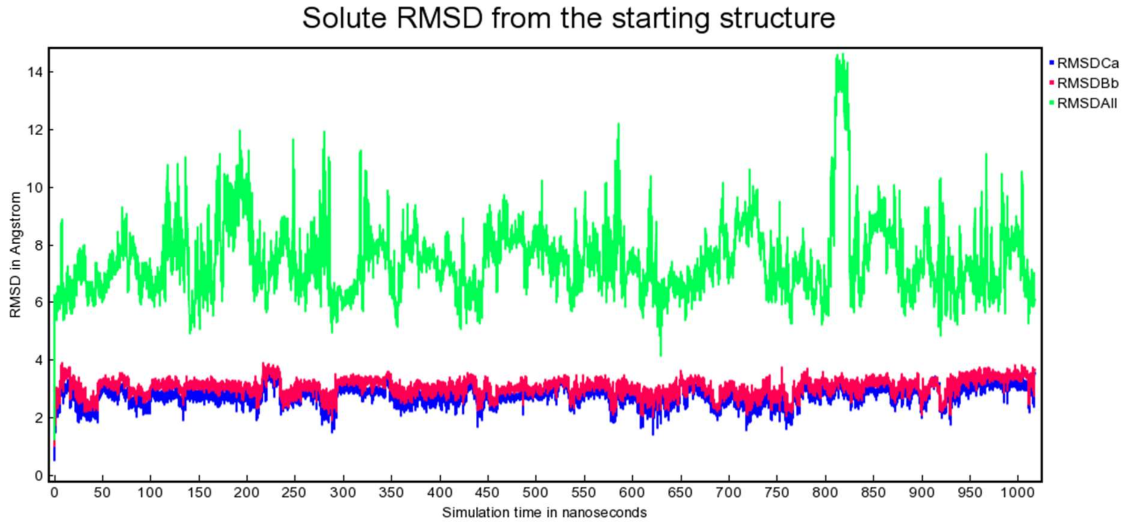

Figure S3: Solute RMSD of the P1a peptide from the starting structure (vertical axis) as a function of simulation time (horizontal axis).

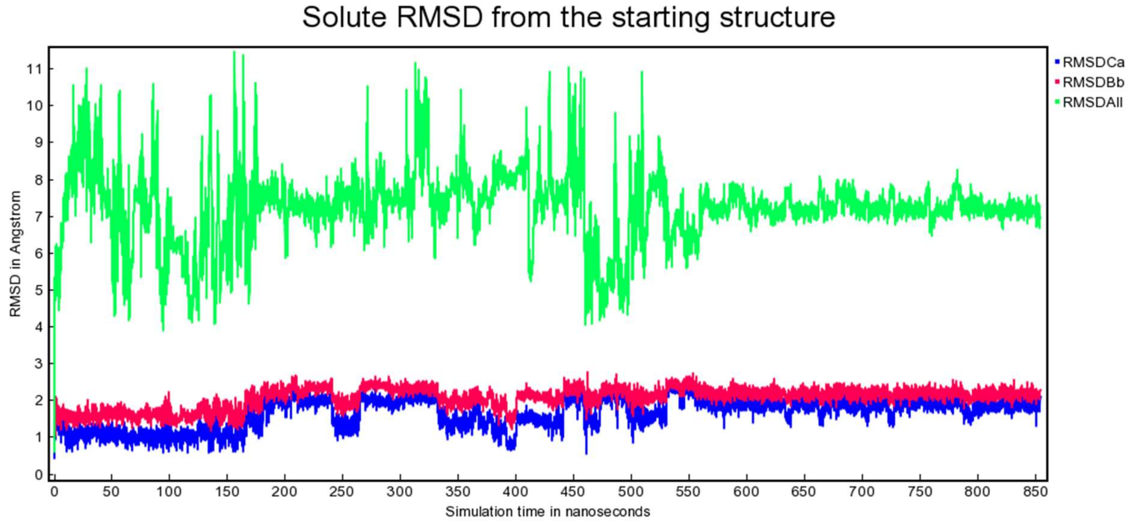

Figure S4: Solute RMSD of the P1c peptide from the starting structure (vertical axis) as a function of simulation time (horizontal axis).

NMR measurements of peptides P1a and P1b

All NMR measurements were performed on a 600 MHz Bruker NEO spectrometer equipped with a Prodigy TCI probe head. The temperature was set to 25°C. The peptides were dissolved at >1.6 mg/mL in MES buffer at pH 5.5. TOCSY spectra were recorded using "dipsi2gpph19" pulse sequence for TopSpin 4, while ROESY were recorded using the "roesygpph19.2" pulse sequence. In the TOCSY experiments, the spectral width was set to 8196.7 Hz in both dimensions. We recorded 256 t_1 incrementents at a mixing time of 150 ms. States-TPPI was used for QUADRATURE detection. ROESY spectra were measured with a spectral width of 5882.4 Hz in both dimensions. 256 t_1 increments at a mixing time of 200 ms were recorded. Again states-TPPI was used for QUADRATURE detection. All NMR spectra were processed using TopSpin, NMRPipe[4] and Sparky.[5]

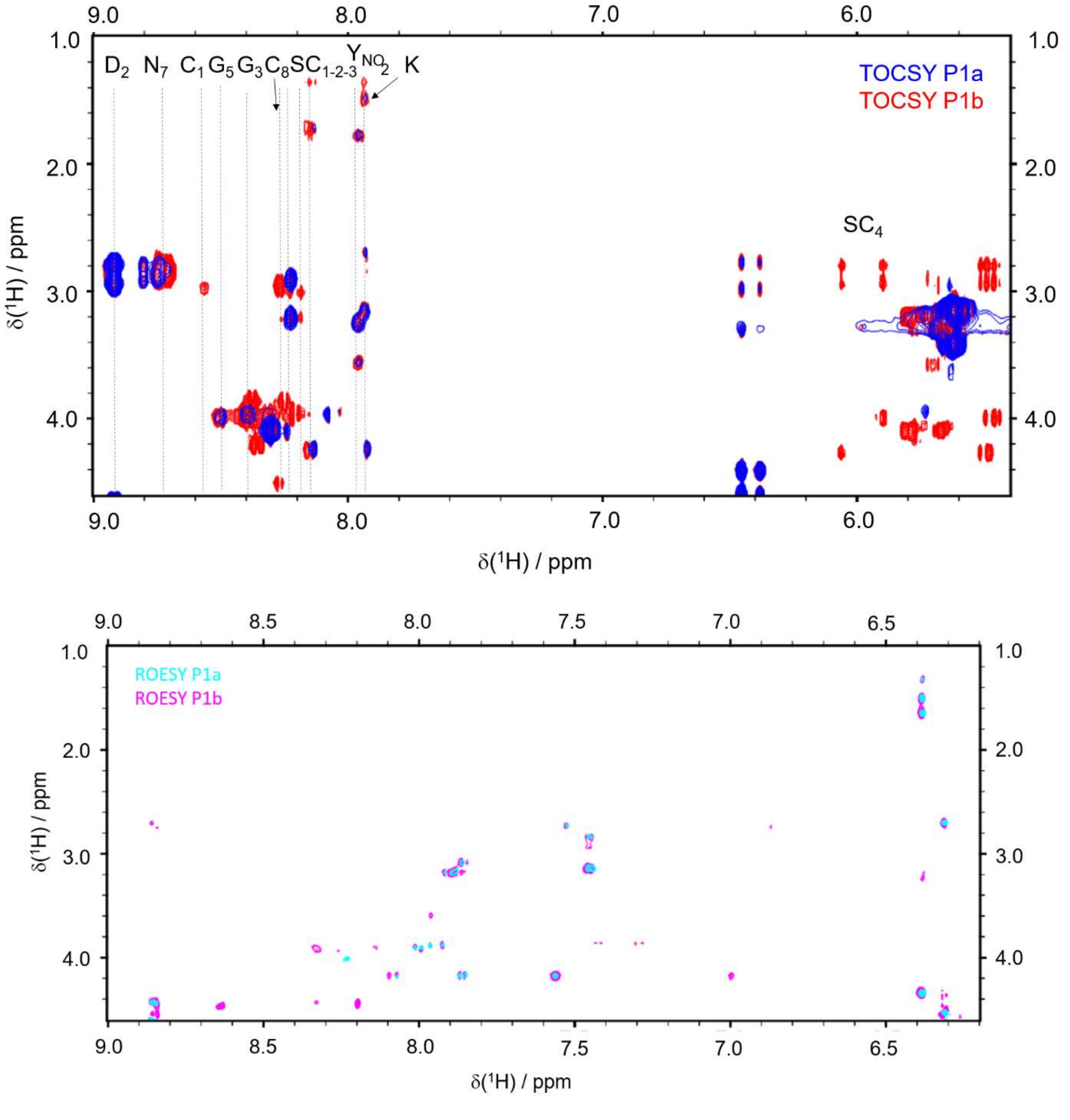

Figure S5: Top: Overlay of ^{1}H–^{1}H TOCSY NMR spectra of the peptides **P1a** in blue and **P1b** in red, respectively. The peptide residue assignments are shown on top. SC is an abbreviation for side chains. The differences in resonance positions indicate that the peptide conformation changes when the staple stereochemistry changes. Bottom**:** Overlay of a ^{1}H–^{1}H ROESY NMR

spectra of the peptides **P1a** (cyan) and **P1c** (magenta). Again different patterns are observed indicating a change in conformation upon switching the staple stereochemistry. All spectra were obtained at a proton Larmor frequency of 600 MHz, at 25°C.

NMR measurements of the two Diels-Alder products

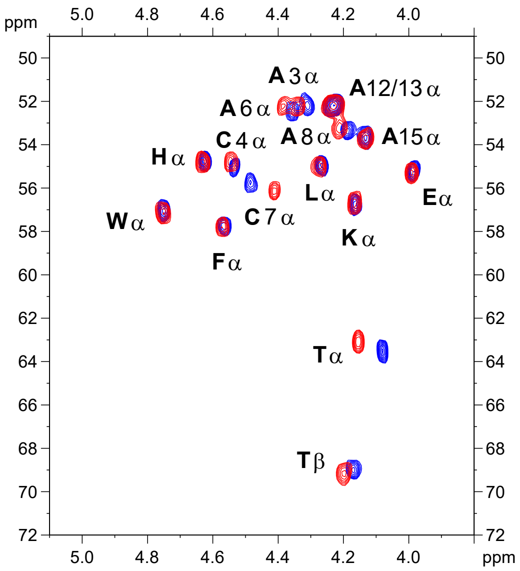

Figure S6: Overlay of the two ^{1}H-^{13}C HSQC spectra of the endo/exo like Diels-Alder products from the reaction of the peptide **P3h** with 3-MPA, showing the region of the CH α signals. (blue: peak 1; red: peak 2).

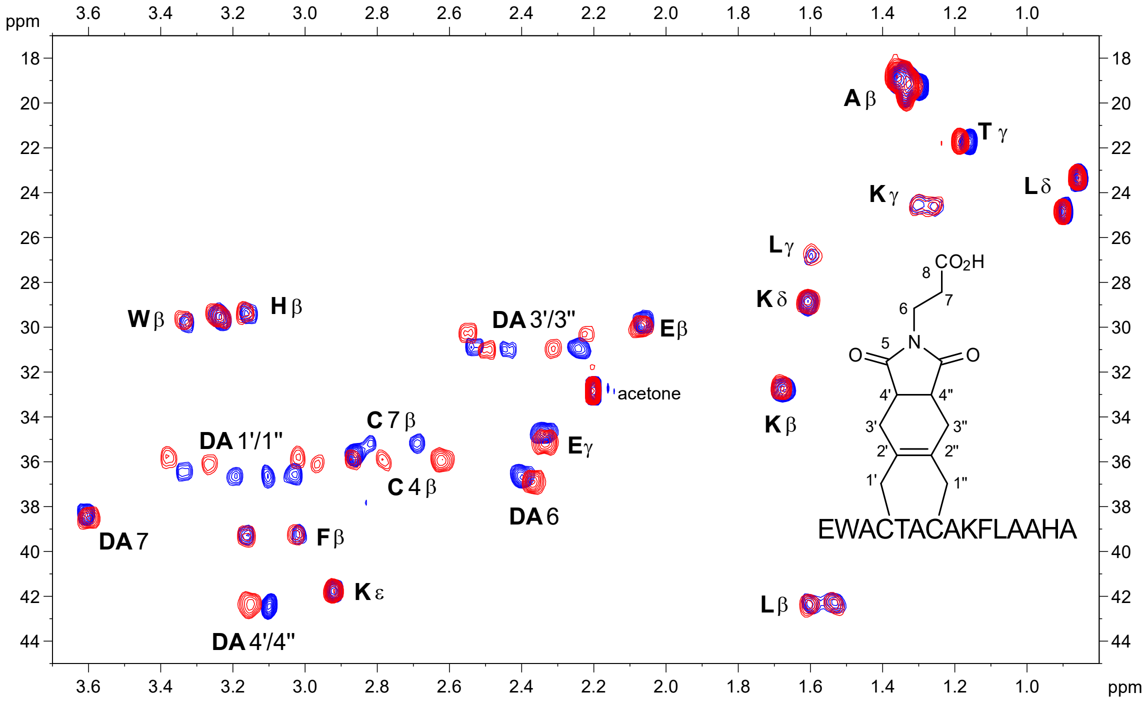

Figure S7: Overlay of the two ^{1}H-^{13}C HSQC spectra of the endo/exo like Diels-Alder products from the reaction of the peptide **P3h** with 3-MPA, showing the region of the CH β and other aliphatic side chain signals together with the CH_2 signals of the DA reaction side. (blue: peak 1; red: peak 2).

no.	aa		C=O	α	β	γ	other
1	E	peak 1 1H		3.99	2.07	2.34	
		peak 1 ^{13}C	172.2	55.3	29.9	34.8	δ: 182.4
		peak 2 ^{1}H		4.00	2.07	2.34	
		peak 2 ^{13}C	172.2	55.4	30.1	35.2	δ: 182.7
2	W	peak 1 ^{1}H		4.75	3.33/3.24		2: 7.28; 4: 7.64; 5: 7.13; 6: 7.22; 7: 7.48
		peak 1 ^{13}C	175.4	57.1	29.9		2: 127.1; 3: 111.7; 4: 121.4; 5: 122.2; 6: 124.7; 7: 114.6; 8: 139.0; 9: 129.5
		peak 2 ^{1}H		4.76	3.34/3.23		2: 7.30; 4: 7.65; 5: 7.14; 6: 7.23; 7: 7.48
		peak 2 ^{13}C	175.6	57.3	29.8		2: 127.0; 3: 111.4; 4: 121.0; 5: 122.1; 6: 124.7; 7: 114.7; 8: 138.8; 9: 129.5
3	A	peak 1 ^{1}H		4.31	1.30		
		peak 1 ^{13}C	176.7	52.2	19.4		
		peak 2 ^{1}H		4.34	1.33		
		peak 2 ^{13}C	177.0	52.3	19.4		
4	C	peak 1 ^{1}H		4.54	2.87		
		peak 1 ^{13}C	174.9	55.1	35.8		
		peak 2 ^{1}H		4.55	2.78/2.62		
		peak 2 ^{13}C	174.8	54.9	36.0		
5	T	peak 1 ^{1}H		4.08	4.17	1.16	
		peak 1 ^{13}C	175.2	63.6	69.1	21.9	
		peak 2 ^{1}H		4.16	4.20	1.19	
		peak 2 ^{13}C	174.7	63.2	69.3	21.8	
6	A	peak 1 ^{1}H		4.36	1.36		
		peak 1 ^{13}C	177.0	52.6	19.1		
		peak 2 ^{1}H		4.39	1.34		
		peak 2 ^{13}C	176.8	52.3	18.9		
7	C	peak 1 ^{1}H		4.49	2.83/2.69		
		peak 1 ^{13}C	174.6	55.8	35.3		
		peak 2 ^{1}H		4.41	2.87/2.63		
		peak 2 ^{13}C	174.9	56.2	36.0		
8	A	peak 1 ^{1}H		4.19	1.35		
		peak 1 ^{13}C	178.1	53.4	18.8		
		peak 2 ^{1}H		4.22	1.37		
		peak 2 ^{13}C	177.9	53.3	18.9		
9	K	peak 1 ^{1}H		4.17	1.68	1.31/1.26	δ: 1.61; ε: 2.92
		peak 1 ^{13}C	176.4	56.8	32.9	24.7	δ: 29.0; ε: 41.9
		peak 2 ^{1}H		4.17	1.68	1.31/1.27	δ: 1.61; ε: 2.93
		peak 2 ^{13}C	176.5	56.8	32.8	24.7	δ: 28.9; ε: 41.9
10	F	peak 1 ^{1}H		4.57	3.17/3.02		2/6: 7.21; 3/5: 7.31; 4: 7.26
		peak 1 ^{13}C	176.0	57.8	39.4		1: 138.8; 2/6: 131.8; 3/5: 131.4; 4 129.9
		peak 2 ^{1}H		4.57	3.17/3.03		2/6: 7.22; 3/5: 7.32; 4: 7.27
		peak 2 ^{13}C	175.9	57.9	39.4		1: 138.8; 2/6: 131.8; 3/5: 131.5; 4 129.9
11	L	peak 1 ^{1}H		4.27	1.61/1.54	1.60	δ: 0.90/0.86
		peak 1 ^{13}C	176.7	55.0	42.4	26.9	δ: 24.9/23.4
		peak 2 ^{1}H		4.28	1.61/1.54	1.60	δ: 0.91/0.87
		peak 2 ^{13}C	176.8	55.1	42.5	26.9	δ: 24.9/23.5
12/13	A	peak 1 ^{1}H		4.24	1.33		
		peak 1 ^{13}C	177.2	52.2	19.1		

		peak 2 ^{1}H		4.24	1.33					
		peak 2 ^{13}C	177.2	52.2	19.1					
	A	peak 1 ^{1}H		4.23	1.36					
		peak 1 ^{13}C	177.2	52.3	19.1					
		peak 2 ^{1}H		4.23	1.37					
		peak 2 ^{13}C	177.3	52.3	19.1					
14	H	peak 1 ^{1}H		4.63	3.25/3.16		2: 8.54; 4: 7.28			
		peak 1 ^{13}C	173.1	54.9	29.5		2: 136.4; 4: 120.4; 5: 130.8			
		peak 2 ^{1}H		4.63	3.25/3.17		2: 8.54; 4: 7.29			
		peak 2 ^{13}C	173.1	54.9	29.5		2: 136.3; 4: 120.4; 5: 130.8			
15	A	peak 1 ^{1}H		4.14	1.34					
		peak 1 ^{13}C	182.8	53.8	19.7					
		peak 2 ^{1}H		4.13	1.34					
		peak 2 ^{13}C	182.9	53.8	19.7					
	DA		1’ or 1’’	2' or 2’’	3' or 3’’	4' or 4’’	5	6	7	8
	DA’	peak 1 ^{1}H	3.20/3.11		2.54/2.26	3.10		2.40	3.61	
		peak 1 ^{13}C	36.7	134.1	31.0	42.5	185.2	36.8	38.5	180.5
		peak 2 ^{1}H	3.27/2.97		2.55/2.23	3.15		2.37	3.60	
		peak 2 ^{13}C	36.2	133.9	30.4	42.5	185.2	37.0	38.6	180.8
	DA’’	peak 1 ^{1}H	3.34/3.04		2.44/2.24	3.10				
		peak 1 ^{13}C	36.5	134.2	31.1	42.5				
		peak 2 ^{1}H	3.38/3.03		2.50/2.32	3.16				
		peak 2 ^{13}C	35.9	133.9	31.1	42.5				

Table S1: ^{1}H and ^{13}C NMR data (δ in ppm) for the endo/exo like Diels-Alder products (peak 1 and 2) from the reaction of the peptide **P3h** with 3-MPA. (no.: sequence number; aa: amino acid; peak 1/2: RP-HPLC peaks; DA: Diels-Alder part of the product, for the numbering see Figure S5).

The NMR analysis of the two endo/exo like DA products from the reaction of the peptide **P3h** with 3-MPA was done on a Bruker Advance III HDX 700 MHz spectrometer using a quadruple Helium cooled cryoprobe (QCIF). The samples (0.5 mg for peak 1 and 0.4 mg for peak 2) were dissolved in 0.5 mL D_2O, all experiments were done at a temperature of 25°C. The spectra were referenced for ^{1}H to the signal of the methyl groups of DSS (δ = 0 ppm). Chemical shifts for ^{13}C are reported on a unified scale relative to ^{1}H using the Ξ value for DSS.

LC-MS/MS measurements to confirm correct Diels-Alder product

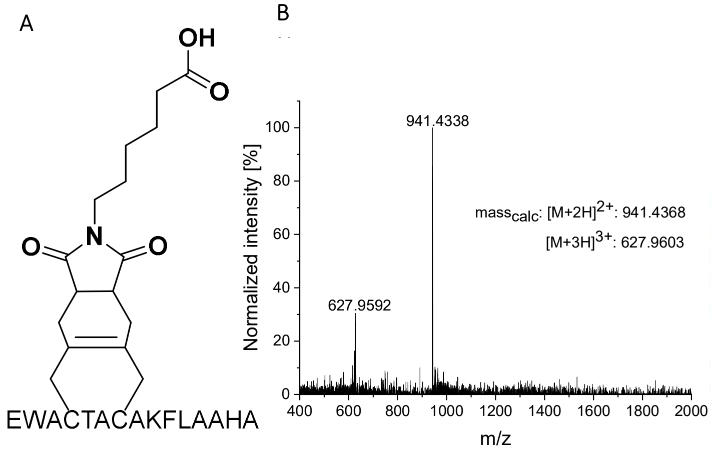

C

b	b^{+2}				y	y^{+2}
---	---	1	**E**	15	---	---
316.1292	---	2	**W**	14	1752.8236	876.9155
387.1663	---	3	**A**	13	1566.7443	783.8758
779.3069	---	4	**C(289.1314)**	12	1495.7072	748.3572
880.3546	---	5	**T**	11	1103.5666	552.2870
951.3917	---	6	**A**	10	1002.5189	501.7631
1054.4009	---	7	**C**	9	931.4818	466.2446
1125.4380	---	8	**A**	8	828.4726	414.7400
1253.5329	627.2701	9	**K**	7	757.4355	379.2214
1400.6013	700.8043	10	**F**	6	629.3406	315.1739
1513.6854	757.3463	11	**L**	5	482.2722	241.6397
1584.7225	792.8649	12	**A**	4	369.1881	185.0977
1655.7596	828.3835	13	**A**	3	298.1510	149.5791
1792.8186	896.9129	14	**H**	2	227.1139	114.0606
---	---	15	**A**	1	90.0550	---

D

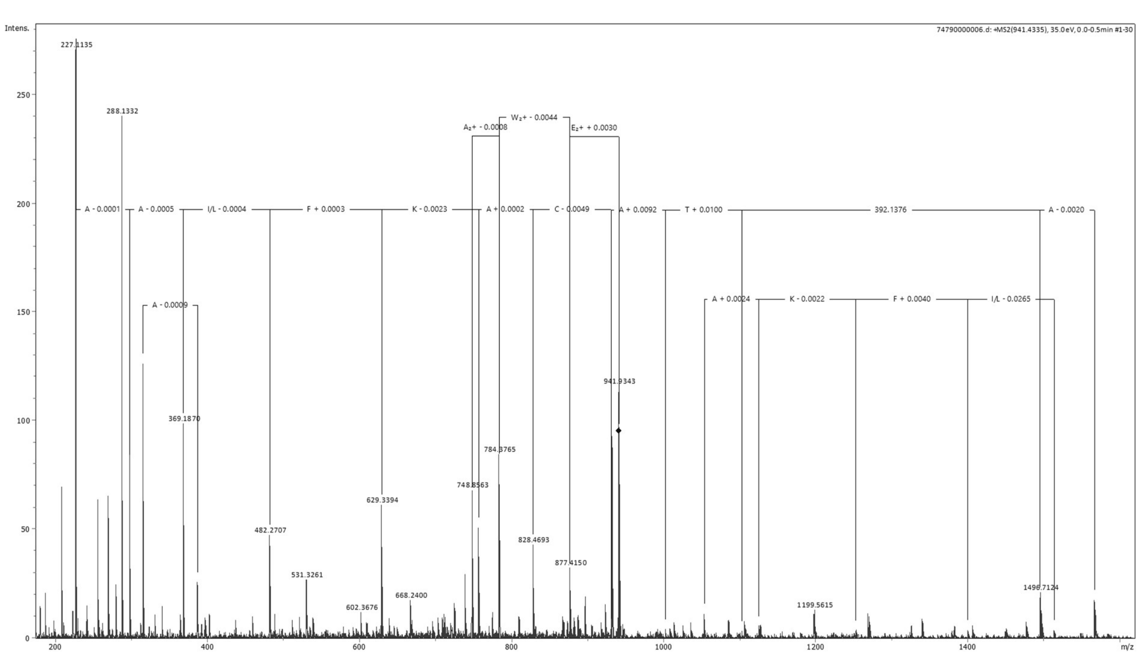

Figure S8: LC-MS/MS results of the Diels-Alder reaction. A: Desired product. B: MS of the modified peptide shown in A. C: Fragmentation calculated by prospector. D: MS/MS of the modified peptide shown in A. The MS/MS data show that the modification occurs selectively on the staple (see 392.1376 on the top right side, 289 Da for the modification and 103 Da for cysteine).

Peptide Stapling

The peptide stapling was performed based on our published protocols.[1] The conversion of the reactions was determined based on HPLC measurements at 214 nm. The peaks around 8.8 min are caused by the catalyst.

Crude **P1**:

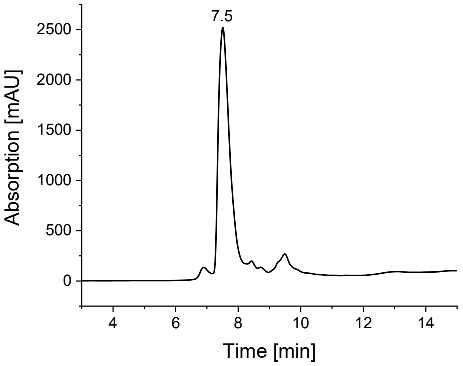

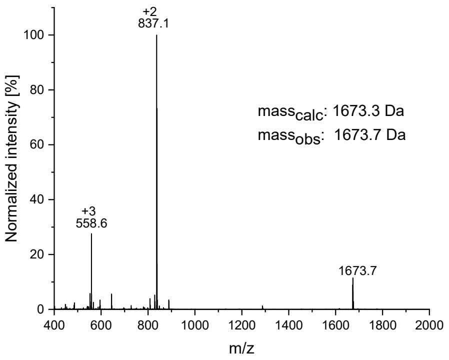

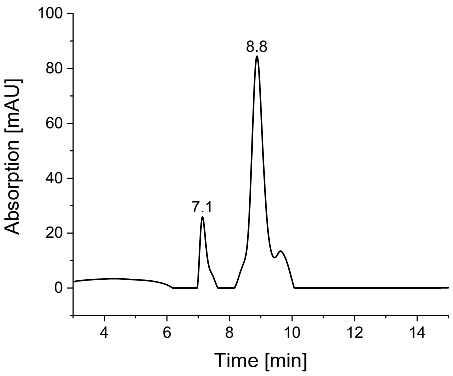

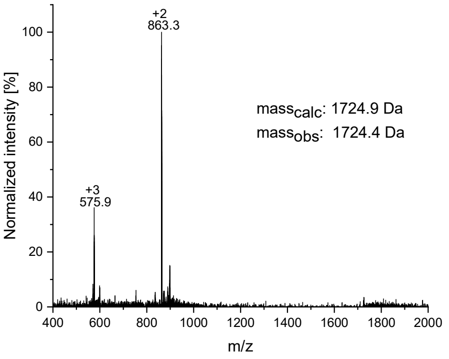

Stapling reaction of **P1** with **Ra**:

Stapling reaction of **P1** with **Rb**:

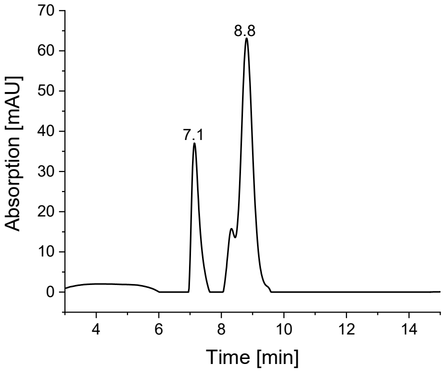

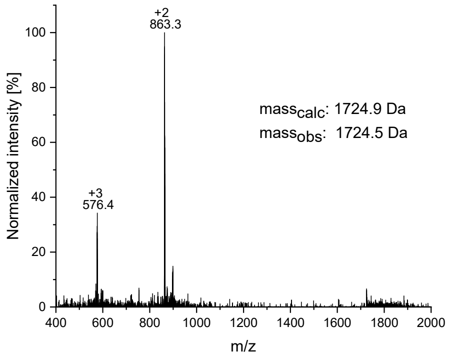

Thiol-ene reactions

6.25 µL of a 50 mM PEG-SH stock solution in 250 mM NaOAc buffer with 6 M Gnd-HCl, pH 5.4 were mixed with 1.25 µL of a 50 mM TCEP stock solution in H_2O and incubated on ice for 5 min. Afterwards, **P3a**/**P3c**, dissolved in 15.3 µL 250 mM NaOAc buffer with 6 M Gnd-HCl, pH 5.4, and 2.22 µL of a 75 mM LAP stock solution in H_2O were added, giving 25 µL of 10 mM **P3a**/**P3c**, 12.5 mM PEG-SH, 2.5 mM TCEP and 6.7 mM LAP. The reaction tube was equipped with a magnetic stirrer, placed on ice and irradiated from above with 365 nm light (83 mW/cm2) for 30 sec total (15 sec on, 15 sec off, 15 sec on, 15 sec off). The light source was placed approx. 5 cm above the sample and the reaction was covered with aluminum foil. The experiment was performed under a gently stream of argon. The buffer and H_2O were degassed with argon for 15 min before setting up the stock solutions and reaction.

For the reaction with **P3a** two products with identical mass during LC-MS analysis were isolated.

Product 1:

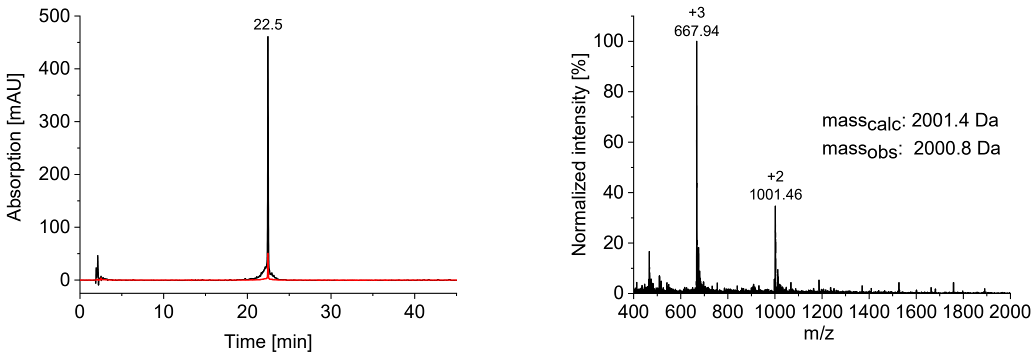

Product 2:

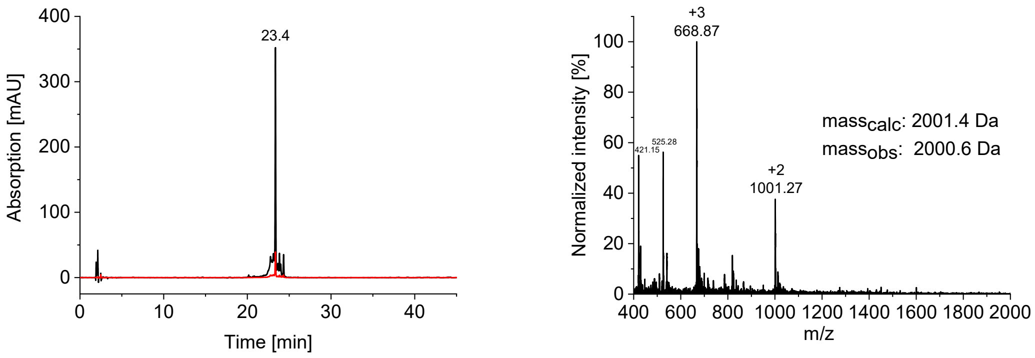

Figure S9: HPLC- and MS analysis of the two isolated products obtained during the thiol-ene reaction with **P3a**.

Diels-Alder reactions

0.1 mg peptide was mixed with 9.6 µL of a 12.5 mM Dansyl or 2.4 µL of a 50 mM 3-MPA/6-MHA stock solution in 50% ACN in H_2O. 50% ACN in H_2O was added to a total volume of 12 µL, giving 5 mM peptide and 10 mM Dansyl/3-MPA/6-MHA.

0.1 mg peptide was dissolved in 57 µL DMF mixed with 3 µL of a 50 mM PTAD stock solution in DMF, giving 60 µL of 5 mM peptide and 10 mM PTAD.

The reaction mixtures were stirred at RT and the reaction progress was monitored by HPLC-MS. 50% ACN and DMF were degassed with argon for 15 min before setting up the stock solutions and reactions.

Synthesis of allylic carbonates

*(Z)-But-2-ene-1,4-diyl dimethyl bis(carbonate) (**Ra**)*[1]

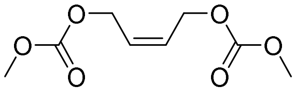

Following the general procedure, (*Z*)-but-2-ene-1,4-diol (2.00 g, 22.7 mmol) was reacted with pyridine (7.33 mL, 90.8 mmol) and methyl chloroformate (7.03 mL, 90.8 mmol) in CH_2Cl_2 (50 mL). General work-up afforded the desired compound as a yellowish oil (4.84 g, quant.).

$C_8H_{12}O_6$ [204.18 g·mol^{-1}]
R_f = 0.76 (cyclohexane:EtOAc = 1:1 (v/v), $KMnO_4$)
GC-MS (method: 50S): t_R = 5.04 min; *m/z* (%) = 128 (26), 85 (33), 69 (100), 59 (90).
b.p. = approx. 130 °C (0.10 mbar)
^{1}H-NMR (300 MHz, $CDCl_3$): δ = 5.80 (t, *J* = 3.9 Hz, 2H), 4.74 (d, *J* = 4.2 Hz, 4H), 3.78 (s, 6H).
^{13}C-NMR (76 MHz, $CDCl_3$): δ = 155.7, 128.1, 63.3, 55.0.
Analytical data are in accordance with the literature.[9]

*(E)-But-2-ene-1,4-diyl dimethyl bis(carbonate) (**Rb**)*

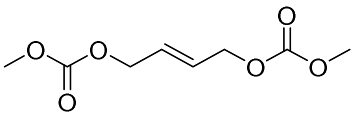

Following the general procedure, (*E*)-but-2-ene-1,4-diol (1.11 g, 12.6 mmol) was reacted with pyridine (4.1 mL, 51 mmol) and methyl chloroformate (3.9 mL, 51 mmol) in CH_2Cl_2 (30 mL). General work-up afforded the desired compound as a colorless solid (2.40 g, 93%).

$C_8H_{12}O_6$ [204.18 g·mol^{-1}]
R_f = 0.76 (cyclohexane:EtOAc = 1:1 (v/v), $KMnO_4$)
GC-MS (method: 50S): t_R = 5.31 min; *m/z* (%) = 128 (29), 85 (31), 69 (100), 59 (99).
m.p. = 65-66 °C
^{1}H-NMR (300 MHz, $CDCl_3$): δ = 5.97-5.81 (m, 2H), 4.71-4.54 (m, 4H), 3.77 (s, 6H).
^{13}C-NMR (76 MHz, $CDCl_3$): δ = 155.6, 128.1, 67.1, 55.0.
Analytical data are in accordance with the literature.[10]

*2-Methylenepropane-1,3-diyl dimethyl bis(carbonate) (**Rc**)*[1]

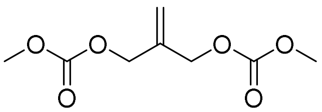

Following the general procedure, 2-methylenepropane-1,3-diol (0.989 g, 11.2 mmol) was reacted with pyridine (3.7 mL, 46 mmol) and methyl chloroformate (3.5 mL, 45 mmol) in CH_2Cl_2 (30 mL). General work-up afforded the desired compound as a yellow oil (2.41 g, quant.).

$C_8H_{12}O_6$ [204.18 g·mol^{-1}]
R_f = 0.57 (cyclohexane:EtOAc = 2:1 (v/v), $KMnO_4$)
b.p. = approx. 125 °C (0.095 mbar)
^{1}H-NMR (300 MHz, $CDCl_3$): δ = 5.34 (s, 2H), 4.66 (s, 4H), 3.78 (s, 6H).
^{13}C-NMR (76 MHz, $CDCl_3$): δ = 155.6, 137.7, 118.3, 67.8, 55.0.
Analytical data are in accordance with the literature.[11]

*But-2-yne-1,4-diyl dimethyl bis(carbonate) (**Rd**)*

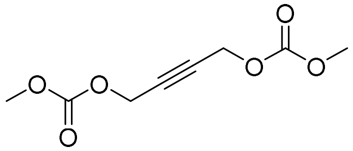

Following the general procedure, but-2-yne-1,4-diol (1.01 g, 11.7 mmol) was reacted with pyridine (3.7 mL, 46 mmol) and methyl chloroformate (3.6 mL, 47 mmol) in CH_2Cl_2 (30 mL). General work-up afforded the desired compound as a yellowish oil (2.45 g, quant.), which solidifies upon standing.

$C_8H_{10}O_6$ [202.16 g·mol^{-1}]
R_f = 0.79 (cyclohexane:EtOAc = 1:1 (v/v), $KMnO_4$)
GC-MS (method: 50S): t_R = 5.43 min; *m/z* (%) = 127 (29), 81 (48), 77 (65), 59 (100).
m.p. < 40 °C
b.p. = approx. 160 °C (0.18 mbar)
^{1}H-NMR (300 MHz, $CDCl_3$): δ = 4.76 (s, 4H), 3.80 (s, 6H).
^{13}C-NMR (76 MHz, $CDCl_3$): δ = 155.2, 81.0, 55.5, 55.3.
Analytical data are in accordance with the literature.[12]

Dimethyl 3,3'-(1,3-phenylene)(2E,2'E)-diacrylate (1)

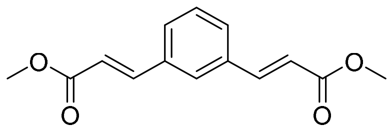

A flame-dried and argon-flushed Schlenk flask, equipped with a Teflon-coated magnetic stirring bar, was charged with $Pd(OAc)_2$ (24.1 mg, 107 µmol), P(*o*Tol)$_3$ (64.5 mg, 212 µmol), K_2CO_3 (1.17 g, 8.47 mmol) and anhydrous DMF (10 mL). The mixture was three times evacuated and argon-flushed before 1,3-dibromobenzene (501 mg, 2.12 mmol) and methyl acrylate (763 µL, 8.47 mmol) were added. The yellowish-brown suspension was then stirred at 100 °C for 22 h (reaction monitoring via GC-MS). In order to reach complete conversion, additional $Pd(OAc)_2$ (12.6 mg, 55.9 µmol), P(*o*Tol)$_3$ (32.5 mg, 107 µmol), K_2CO_3 (590 mg, 4.27 mmol) and methyl acrylate (382 µL, 4.24 mmol) was added and the suspension was stirred for another 6 h at 100 °C. The reaction mixture was cooled to r.t., diluted with EtOAc (50 mL), washed with 1 M LiCl (5 x 20 mL), dried over Na_2SO_4, filtered and concentrated under reduced pressure to give the desired compound as a yellow solid (650 mg, quant.).

$C_{14}H_{14}O_4$ [246.26 g·mol^{-1}]
m.p. = 126-128 °C
GC-MS (method: 50S): t_R = 7.38 min; *m/z* (%) = 246 (43), 231 (100), 215 (42), 199 (26).
^{1}H-NMR (300 MHz, $CDCl_3$): δ = 7.68 (d, *J* = 16.1 Hz, 2H), 7.63 (s, 1H), 7.53 (d, *J* = 7.4 Hz, 2H), 7.41 (t, *J* = 7.6 Hz, 1H), 6.47 (d, *J* = 16.0 Hz, 2H), 3.81 (s, 6H).
^{13}C-NMR (76 MHz, $CDCl_3$): δ = 167.3, 144.0, 135.3, 129.6, 127.8, 119.0, 51.9.
Analytical data are in accordance with the literature.[13]

*(2E,2'E)-1,3-Phenylenebis(prop-2-ene-3,1-diyl) dimethyl bis(carbonate) (**Re**)*

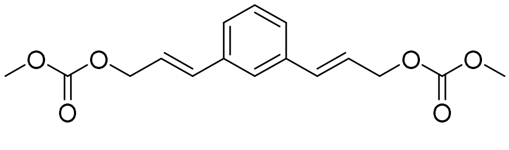

In an evacuated and argon-flushed 100 mL round-bottom flask, equipped with a Teflon-coated magnetic stirring bar, **1** (615 mg, 2.50 mmol) was dissolved in anhydrous CH_2Cl_2 (20 mL) and cooled to -78 °C (dry ice/acetone). Subsequently, DIBAL-H (15.0 mL, 1.0 M soln. in CH_2Cl_2, 15.0 mmol) was added over a period of 20 min resulting in an orange solution, which was stirred at -78 °C for 30 min. In order to reach complete consumption of the starting material (according to HPLC-MS), additional DIBAL-H (3.5 mL, 1.0 M soln. in CH_2Cl_2, 3.5 mmol) was added and the solution was allowed to warm to -10 °C over a period of 2 h. The reaction mixture was then quenched by the addition of satd. Rochelle salt (45 mL) and the CH_2Cl_2 was removed under reduced pressure. Subsequently, EtOAc (30 mL) was added and the mixture was vigorously stirred overnight. The aqueous phase was extracted with EtOAc (2 x 30 mL) and the combined organic layers were dried over Na_2SO_4, filtered and concentrated under reduced pressure to give the crude allylic alcohol.

In a 50 mL round-bottom flask, equipped with a Teflon-coated magnetic stirring bar, the crude intermediate was dissolved in CH_2Cl_2 (20 mL) and cooled to 0 °C (ice bath). Subsequently, pyridine (808 µL, 10.0 mmol) and methyl chloroformate (0.775 mL, 10.0 mmol) were added and the reaction mixture was stirred at r.t. for 30 min (reaction monitoring via HPLC-MS). After work-up following the general procedure, the crude product was purified via flash column chromatography (75 g SiO_2, 20.0 x 3.0 cm, cyclohexane:EtOAc = 4:1 to 3:1 (v/v)) to give the desired compound as a yellowish solid (459 mg, 60% over two steps).

$C_{16}H_{18}O_6$ [306.31 g·mol^{-1}]
R_f = 0.25 (cyclohexane:EtOAc = 4:1 (v/v), $KMnO_4$)
HPLC-MS (method: 2-100-EC-C18): t_R = 5.49 min; *m/z* (ESI+) = 329 $[M+Na]^+$.
m.p. = 78-80 °C
^{1}H-NMR (300 MHz, $CDCl_3$): δ =7.40 (s, 1H), 7.29 (br s, 3H), 6.67 (d, *J* = 15.9 Hz, 2H), 6.30 (dt, *J* = 15.8, 6.3 Hz, 2H), 4.79 (d, *J* = 6.2 Hz, 4H), 3.81 (s, 6H).
^{13}C-NMR (76 MHz, $CDCl_3$): δ = 155.8, 136.6, 134.5, 129.0, 126.6, 125.2, 123.1, 68.4, 55.0.
No HRMS data could be obtained.

Diethyl 3,3'-(1,4-phenylene)(2E,2'E)-diacrylate (2)
This compound was prepared similar to a procedure described by Einaru et al.[14]

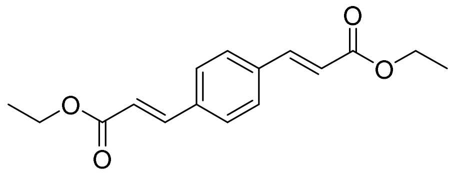

In a flame-dried and argon-flushed Schlenk flask, equipped with a Teflon-coated magnetic stirring bar and dropping funnel, NaH (482 mg, 60% dispersion in mineral oil, 12.1 mmol) was suspended in anhydrous THF (15 mL) and cooled to 0 °C (ice bath). Subsequently, triethyl phosphonoacetate (2.4 mL, 12 mmol) was added over a period of 10 min. After another 10 min a solution of benzene-1,4-dicarboxaldehyde (669 mg, 4.99 mmol) in anhydrous THF (5 mL) was added over a period of 5 min. The resulting rose solution was warmed to r.t. and then heated to 60 °C for 1 h. Upon complete consumption of the starting material (according to TLC), the reaction mixture was cooled to r.t., washed with satd. NH_4Cl (20 mL) and the aqueous phase was back-extracted with EtOAc (2 x 10 mL). The combined organic layers were washed with 0.1 M NaOH (20 mL) and the aqueous phase was back-extracted with EtOAc (2 x 10 mL). The combined organic layers were washed with satd. $NaHCO_3$ (20 mL), dried over Na_2SO_4, filtered and concentrated under reduced pressure. The crude product was purified via flash column chromatography (50 g SiO_2, 10.0 x 4.0 cm, cyclohexane:EtOAc = 10:1 to 6:1 (v/v)) to give the desired compound as a colorless solid (1.11 g, 81%).

$C_{16}H_{18}O_4$ [274.32 g·mol^{-1}]
R_f = 0.30 (cyclohexane:EtOAc = 6:1 (v/v), $KMnO_4$)
m.p. = 97-100 °C
^{1}H-NMR (300 MHz, $CDCl_3$): δ = 7.66 (d, *J* = 16.0 Hz, 2H), 7.53 (s, 4H), 6.46 (d, *J* = 16.0 Hz, 2H), 4.27 (q, *J* = 7.1 Hz, 4H), 1.34 (t, *J* = 7.1 Hz, 6H).
^{13}C-NMR (76 MHz, $CDCl_3$): δ = 166.9, 143.5, 136.3, 128.6, 119.5, 60.8, 14.4.
Analytical data are in accordance with the literature.[15]

*(2E,2'E)-1,4-Phenylenebis(prop-2-ene-3,1-diyl) dimethyl bis(carbonate) (**Rf**)*

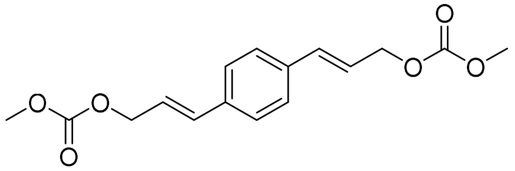

In a flame-dried and argon-flushed Schlenk flask, equipped with a Teflon-coated magnetic stirring bar, **2** (200 mg, 730 µmol) was dissolved in anhydrous CH_2Cl_2 (7 mL) and cooled to -78 °C (dry ice/acetone). Subsequently, DIBAL-H (3.3 mL, 1.0 M soln. in CH_2Cl_2, 3.3 mmol) was added over a period of 10 min resulting in a bright yellow solution, which was stirred at -78 °C for 30 min. Upon complete consumption of the starting material (according to HPLC-MS), the reaction mixture was warmed to 0 °C, diluted with Et_2O (10 mL) and consecutively treated with H_2O (132 µL), 15% NaOH (132 µL), H_2O (330 µL) and stirred at r.t. The mixture was dried over $MgSO_4$, filtrated through a pad of Celite (3.0 x 3.5 cm) and washed with EtOAc (50 mL). The filtrate was concentrated under reduced pressure to give the crude allylic alcohol. (*NOTE: Better phase separation and higher overall yields were usually observed when using a different work-up procedure. To this end, the cold reaction mixture was quenched by addition of satd. Rochelle salt, followed by the removal of CH_2Cl_2 under reduced pressure. Then EtOAc was added and the resulting mixture was vigorously stirred overnight.*)

In a 50 mL round-bottom flask, equipped with a Teflon-coated magnetic stirring bar, the crude intermediate (49.2 mg, 259 µmol) was suspended in CH_2Cl_2 (2 mL) and cooled to 0 °C (ice bath). Subsequently, pyridine (83.7 µL, 1.03 mmol) and methyl chloroformate (79.9 µL, 1.03 mmol) was added and the reaction mixture was stirred at r.t. for 30 min. In order to reach full conversion (reaction monitoring via HPLC-MS) additional pyridine (83.7 µL, 1.03 mmol) and methyl chloroformate (79.9 µL, 1.03 mmol) were added. After work-up following the general procedure, the crude product was purified via flash column chromatography (25 g SiO_2, 17.0 x 1.0 cm, cyclohexane:EtOAc = 4:1 (v/v) to CH_2Cl_2) to give the desired compound as a colorless solid (55.9 mg, 25% over two steps).

$C_{16}H_{18}O_6$ [306.31 g·mol^{-1}]
R_f = 0.33 (cyclohexane:EtOAc = 4:1 (v/v), $KMnO_4$)
HPLC-MS (method: 2-100-EC-C18): t_R = 3.56 min; *m/z* (ESI+) = 173.
m.p. = 133-135 °C
^{1}H-NMR (300 MHz, $CDCl_3$): δ = 7.34 (s, 4H), 6.65 (d, *J* = 15.9 Hz), 6.28 (dt, *J* = 15.8, 6.4 Hz, 2H), 4.78 (d, *J* = 6.2 Hz, 4H), 3.80 (s, 6H).
^{13}C-NMR (76 MHz, $CDCl_3$): δ = 155.7, 136.0, 134.3, 127.0, 122.8, 68.4, 54.9.
HRMS (DI/GC-EI-TOF): calc. for $C_{16}H_{18}O_6^+$ $[M]^+$: 306.1104; found: 306.1105.

Diethyl 3,3'-([1,1'-biphenyl]-4,4'-diyl)(2E,2'E)-diacrylate (3)
This compound was prepared similar to a procedure described by Einaru et al.[16]

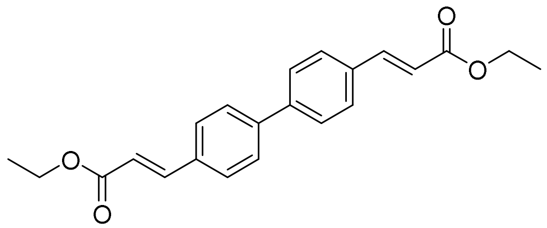

In a flame-dried and argon-flushed Schlenk flask, equipped with a Teflon-coated magnetic stirring bar and dropping funnel, NaH (276 mg, 60% dispersion in mineral oil, 6.9 mmol) was suspended in anhydrous THF (5 mL) and cooled to 0 °C (ice bath). Subsequently, triethyl phosphonoacetate (1.4 mL, 7.1 mmol) was added over a period of 10 min. After another 10 min a solution of 4,4'-biphenyldicarboxaldehyde (603 mg, 2.87 mmol) in anhydrous THF (10 mL) was added over a period of 5 min. The resulting yellowish suspension was warmed to r.t. and then heated to 60 °C for 3 h. Upon complete consumption of the starting material (according to TLC), the reaction mixture was cooled to r.t., washed with satd. NH_4Cl (10 mL) and the aqueous phase was back-extracted with EtOAc (3 x 10 mL). The combined organic layers were dried over Na_2SO_4, filtered and concentrated under reduced pressure. The crude product was adsorbed on 4 g SiO_2 and purified via flash column chromatography (50 g SiO_2, 8.5 x 4.0 cm, cyclohexane:EtOAc = 8:1 to 1:1 (v/v)) to give the desired compound as a yellowish solid (836 mg, 83%).

$C_{22}H_{22}O_4$ [350.41 g·mol^{-1}]
R_f = 0.22 (cyclohexane:EtOAc = 8:1 (v/v), $KMnO_4$)
m.p. = 146-148 °C
^{1}H-NMR (300 MHz, $CDCl_3$): δ = 7.72 (d, *J* = 16.0 Hz, 2H), 7.67-7.50 (m, 8H), 6.48 (d, *J* = 16.0 Hz, 2H,), 4.28 (q, *J* = 7.1 Hz, 4H), 1.35 (t, *J* = 7.1 Hz, 6H).
^{13}C-NMR (76 MHz, $CDCl_3$): δ = 167.1, 144.0, 142.0, 134.1, 128.8, 127.5, 118.6, 60.7, 14.5.
Analytical data are in accordance with the literature.[17]

(2E,2'E)-[1,1'-Biphenyl]-4,4'-diylbis(prop-2-ene-3,1-diyl) dimethyl bis(carbonate) (Rg)

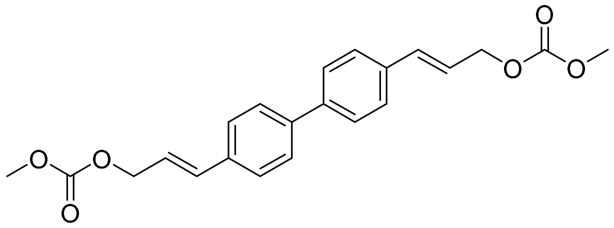

In a flame-dried and argon-flushed Schlenk flask, equipped with a Teflon-coated magnetic stirring bar, **3** (551 mg, 1.57 mmol) was dissolved in anhydrous CH_2Cl_2 (15 mL) and cooled to -78 °C (dry ice/acetone). Subsequently, DIBAL-H (7.2 mL, 1.0 M soln. in CH_2Cl_2, 7.2 mmol) was added over a period of 15 min resulting in a bright orange solution, which was stirred at -78 °C for 30 min. Upon complete consumption of the starting material (according to HPLC-MS), the reaction mixture was warmed to 0 °C, diluted with Et_2O (20 mL) and consecutively treated with H_2O (288 µL), 15% NaOH (288 µL), H_2O (720 µL) and stirred at r.t. The mixture was dried over $MgSO_4$, filtrated through a pad of Celite (1.0 x 6.0 cm) and washed with EtOAc (100 mL) and EtOAc:MeOH = 1:1 (2 x 80 mL). The filtrate was concentrated under reduced pressure to give the crude allylic alcohol. (*NOTE: Better phase separation and higher overall yields were usually observed when using a different work-up procedure. To this end, the cold reaction mixture was quenched by addition of satd. Rochelle salt, followed by the removal of CH_2Cl_2 under reduced pressure. Then EtOAc was added and the resulting mixture was vigorously stirred overnight.*)

In a 50 mL round-bottom flask, equipped with a Teflon-coated magnetic stirring bar, the crude intermediate (78 mg, 293 µmol) was suspended in CH_2Cl_2 (2 mL) and cooled to 0 °C (ice bath). Subsequently, pyridine (94.8 µL, 1.17 mmol) and methyl chloroformate (90.5 µL, 1.17 mmol) were added and the reaction mixture was stirred at r.t. for 15 min. In order to reach full conversion (reaction monitoring via HPLC-MS) three additional portions of pyridine (3 x 94.8 µL, 3 x 1.17 mmol) and methyl chloroformate (3 x 90.5 µL, 3 x 1.17 mmol) were added. After work-up following the general procedure, the crude product was purified via flash column chromatography (12 g SiO_2, 14.5 x 1.5 cm, cyclohexane:EtOAc = 4:1 (v/v) to CH_2Cl_2) to give the desired compound as a colorless solid (54.7 mg, 9% over two steps).

$C_{22}H_{22}O_6$ [382.41 g·mol^{-1}]
R_f = 0.53 (CH_2Cl_2, $KMnO_4$)
HPLC-MS (method: 2-100-EC-C18): t_R = 6.13 min; *m/z* (ESI+) = 405 [M+Na]$^+$.
m.p. = 207-208 °C
^{1}H-NMR (300 MHz, $CDCl_3$): δ = 7.57 (d, *J* = 8.2 Hz, 4H), 7.46 (d, *J* = 8.2 Hz, 4H), 6.72 (d, *J* = 15.9 Hz, 2H), 6.34 (dt, *J* = 15.8, 6.4 Hz, 2H), 4.82 (d, *J* = 5.6 Hz, 4H), 3.82 (s, 6H).
^{13}C-NMR (76 MHz, $CDCl_3$): δ = 155.8, 140.4, 135.4, 134.4, 127.3, 127.3, 122.8, 68.6, 55.0.
HRMS (DI/GC-EI-TOF): calc. for $C_{22}H_{22}O_6^+$ [M]$^+$: 382.1416; found: 382.1413.

*2,3-Dimethylenebutane-1,4-diyl dimethyl bis(carbonate) (**Rh**)*
This compound was prepared similar to a procedure described by Hirata et al.[18]

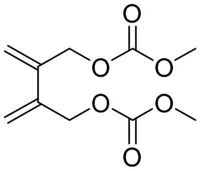

In a flame-dried and argon-flushed Schlenk flask, Grubbs catalyst 2nd generation (20.9 mg, 24.6 µmol) and **Rd** (502 mg, 2.49 mmol) were dissolved in anhydrous toluene (10 mL) and a balloon filled with ethylene (~600 mL) was attached to the flask. The flask was then evacuated, flushed with ethylene and heated to 80 °C for 24 h (reaction monitoring via GC-MS). In order to reach complete conversion, additional Grubbs catalyst 2nd generation (21.4 mg, 25.2 µmol) as well as a freshly filled ethylene-balloon were added and the mixture was stirred at 80 °C for another 24 h. This procedure was repeated for a third time before complete conversion was indicated by GC-MS. The reaction mixture was cooled to r.t., quenched by the addition of ethyl vinyl ether (5 mL), filtrated through a pad of SiO_2 (1.5 x 6.0 cm), washed with EtOAc (100 mL) and the filtrate was concentrated under reduced pressure. The crude product was purified via flash column chromatography (55 g SiO_2, 17.0 x 3.0 cm, cyclohexane:EtOAc = 6:1 (v/v)) to give the desired compound as a colorless solid (325 mg, 57%).

$C_{10}H_{14}O_6$ [230.22 g·mol^{-1}]
R_f = 0.27 (cyclohexane:EtOAc = 6:1 (v/v), $KMnO_4$)
m.p. = 65-67 °C
^{1}H-NMR (300 MHz, $CDCl_3$): δ = 5.38 (d, *J* = 10.7 Hz, 4H), 4.83 (s, 4H), 3.79 (s, 6H).
^{13}C-NMR (76 MHz, $CDCl_3$): δ = 155.6, 138.8, 116.8, 68.4, 55.0.
HRMS (DI/GC-EI-TOF): calc. for $C_{10}H_{14}O_6^+$ [M]$^+$: 230.0790; found: 230.0780.

Synthesis of propargylic and allylic stapling reagents

Buta-1,3-diene-2,3-diylbis(octylsulfane) (4)

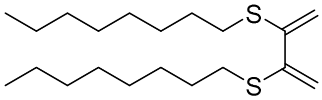

Following the general procedure, **Rd** (61.5 mg, 304 µmol) and 1-octanethiol (88.7 mg, 606 µmol) were reacted in the presence of $Pd(dba)_2$ (7.0 mg, 12 µmol) and BIPHEPHOS (9.4 mg, 12 µmol) in 2.0 mL anhydrous CH_3CN. Purification via flash column chromatography (5 g SiO_2, 12.0 x 1.0 cm, cyclohexane) afforded the desired compound as a yellow oil (87.0 mg, 84%).

$C_{20}H_{38}S_2$ [342.64 g·mol^{-1}]
R_f = 0.50 (cyclohexane, $KMnO_4$)
GC-MS (method: 50S): t_R = 7.91 min; *m/z* (%) = 342 (26), 243 (9), 229 (100), 143 (21).
^{1}H-NMR (300 MHz, $CDCl_3$): δ = 5.60 (s, 2H), 5.10 (s, 2H), 2.69 (t, *J* = 7.3 Hz, 4H), 1.70-1.52 (m, 4H), 1.47-1.14 (m, 20H), 0.99-0.78 (m, 6H).
^{13}C-NMR (76 MHz, $CDCl_3$): δ = 143.8, 112.4, 32.4, 31.9, 29.3, 29.1, 28.7, 22.8, 14.2.
HRMS (DI/GC-EI-TOF): calc. for $C_{20}H_{38}S_2^+$ $[M]^+$: 342.2415; found: 342.2414.

6,7-Bis(octylthio)-2-phenyl-5,8-dihydro-1H-[1,2,4]triazolo[1,2-a]pyridazine-1,3(2H)-dione (5)

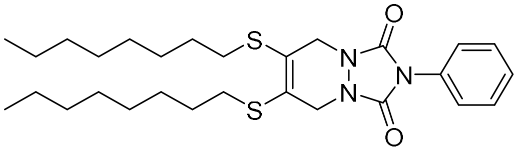

In a 10 mL round-bottom flask, equipped with a Teflon-coated magnetic stirring bar, **4** (66.2 mg, 193 µmol) and PTAD (38.2 mg, 218 µmol) were dissolved in CH_2Cl_2 (1 mL) and stirred at r.t. for 30 min. Upon complete consumption of the starting material (according to TLC), the reaction mixture was concentrated under reduced pressure. The crude product was purified via flash column chromatography (15 g SiO_2, 21.0 x 1.3 cm, cyclohexane) to afford the desired compound as a yellowish oil (63 mg, 63%).

$C_{28}H_{43}N_3O_2S_2$ [517.79 g·mol^{-1}]
R_f = 0.31 (cyclohexane, $KMnO_4$)
GC-MS (method: 50S): t_R = 7.31 min; *m/z* (%) = 281 (27), 233 (100), 207 (58), 144 (34), 120 (36).
^{1}H-NMR (300 MHz, $CDCl_3$): δ = 7.60-7.29 (m, 5H), 4.31 (s, 4H), 2.78 (t, *J* = 7.3 Hz, 4H), 1.67-1.51 (m, 4H), 1.48-1.12 (m, 20H), 0.87 (t, *J* = 6.8 Hz, 6H).
^{13}C-NMR (76 MHz, $CDCl_3$): δ = 152.4 , 131.1, 129.3, 128.4, 126.5, 125.5, 47.6, 31.9, 31.8, 29.9, 29.2, 29.2, 28.8, 22.7, 14.2.
HRMS (DI/GC-EI-TOF): calc. for $C_{28}H_{43}N_3O_2S_2^+$ $[M]^+$: 517.2790; found: 517.2797.

(2,3-Dimethylenebutane-1,4-diyl)bis(octylsulfane) (6)

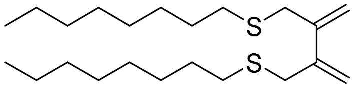

Following the general procedure, **Rh** (35.2 mg, 153 µmol) and 1-octanethiol (43.9 mg, 300 µmol) were reacted in the presence of $Pd(dba)_2$ (3.4 mg, 5.9 µmol) and BIPHEPHOS (4.8 mg, 6.1 µmol) in 1.0 mL anhydrous CH_3CN. Purification via flash column chromatography (5 g SiO_2, 13.0 x 1.0 cm, cyclohexane to cyclohexane:EtOAc = 50:1 (v/v)) afforded the desired compound as a yellow oil (48.7 mg, 88%).

$C_{22}H_{42}S_2$ [370.70 g·mol^{-1}]
R_f = 0.12 (cyclohexane:EtOAc = 100:1 (v/v), $KMnO_4$)
^{1}H-NMR (300 MHz, $CDCl_3$): δ = 5.29 (s, 2H), 5.14 (s, 2H), 3.36 (s, 4H), 2.45 (t, *J* = 7.4 Hz, 4H), 1.65-1.46 (m, 4H), 1.46-1.07 (m, 20H), 0.98-0.75 (m, 6H).
^{13}C-NMR (76 MHz, $CDCl_3$): δ = 142.1, 115.9, 35.8, 31.9, 31.8, 29.4, 29.3, 29.1, 22.8, 14.2.
HRMS (LC-ESI-MS/MS): calc. for $C_{22}H_{43}S_2^+$ [M+H]$^+$: 371.2806; found: 371.2800.

6,7-Bis((octylthio)methyl)-2-phenyl-5,8-dihydro-1H-[1,2,4]triazolo[1,2-a]pyridazine-1,3(2H)-dione (7)

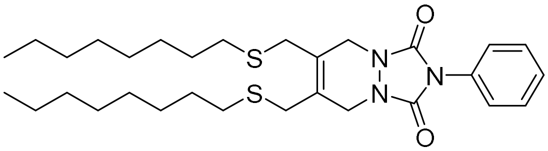

In a 10 mL round-bottom flask, equipped with a Teflon-coated magnetic stirring bar, **6** (47.3 mg, 128 µmol) and PTAD (24.8 mg, 142 µmol) were dissolved in CH_2Cl_2 (1 mL) and stirred at r.t. for 30 min. Upon complete consumption of the starting material (according to TLC), the reaction mixture was concentrated under reduced pressure. The crude product was purified via flash column chromatography (6 g SiO_2, 13.5 x 1.0 cm, cyclohexane:EtOAc = 5:1 (v/v)) to afford the desired compound as a colorless oil (58.0 mg, 83%).

$C_{30}H_{47}N_3O_2S_2$ [545.85 g·mol^{-1}]
R_f = 0.37 (cyclohexane:EtOAc = 5:1 (v/v), $KMnO_4$)
^{1}H-NMR (300 MHz, $CDCl_3$): δ = 7.58-7.42 (m, 4H), 7.36 (t, *J* = 7.0 Hz, 1H), 4.28 (s, 4H, H1), 3.32 (s, 4H), 2.51 (t, *J* = 7.3 Hz, 4H), 1.73-1.49 (m, 4H), 1.49-1.04 (m, 20H), 0.98-0.78 (m, 6H).
^{13}C-NMR (76 MHz, $CDCl_3$): δ = 152.5, 131.3, 129.3, 128.2, 125.9, 125.5, 45.5, 32.7, 31.9, 31.5, 29.7, 29.3, 29.3, 29.0, 22.7, 14.2.
HRMS (LC-ESI-MS/MS): calc. for $C_{30}H_{48}N_3O_2S_2^+$ [M+H]$^+$: 546.3188; found: 546.3185.

NMR-Spectra

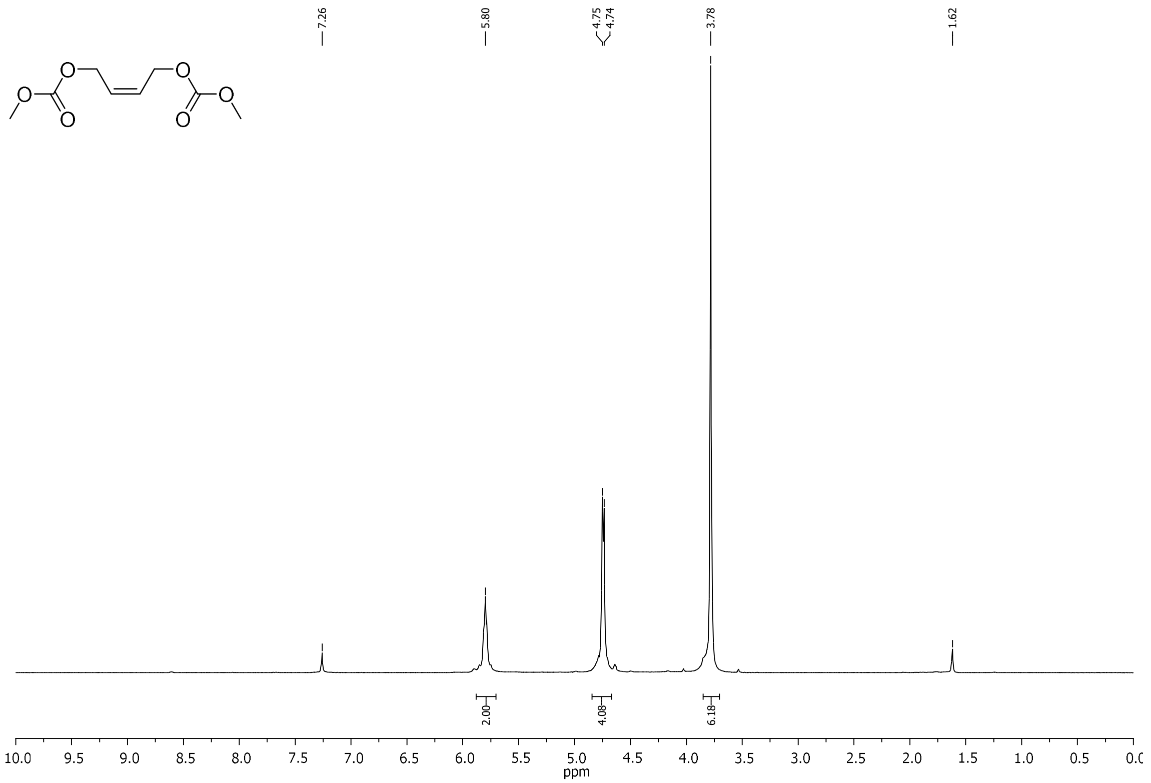

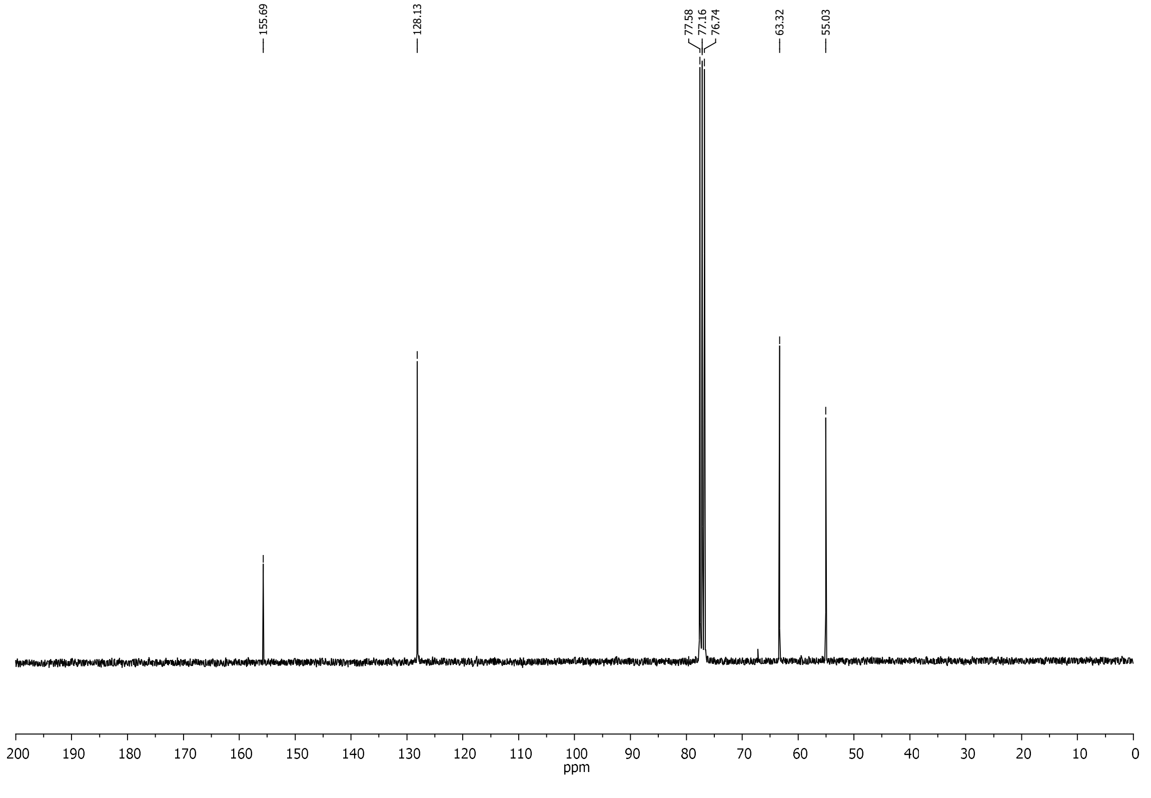

Compound **Ra**: ^{1}H-NMR (300 MHz, $CDCl_3$); ^{13}C-NMR (76 MHz, $CDCl_3$)

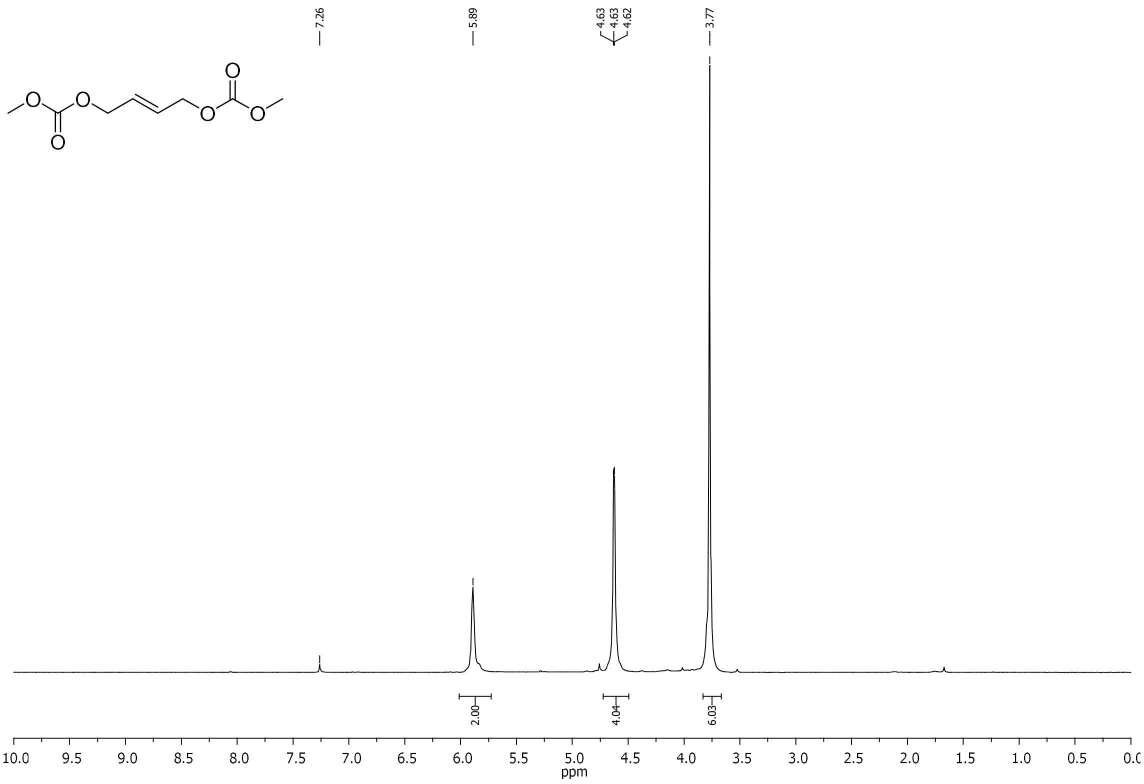

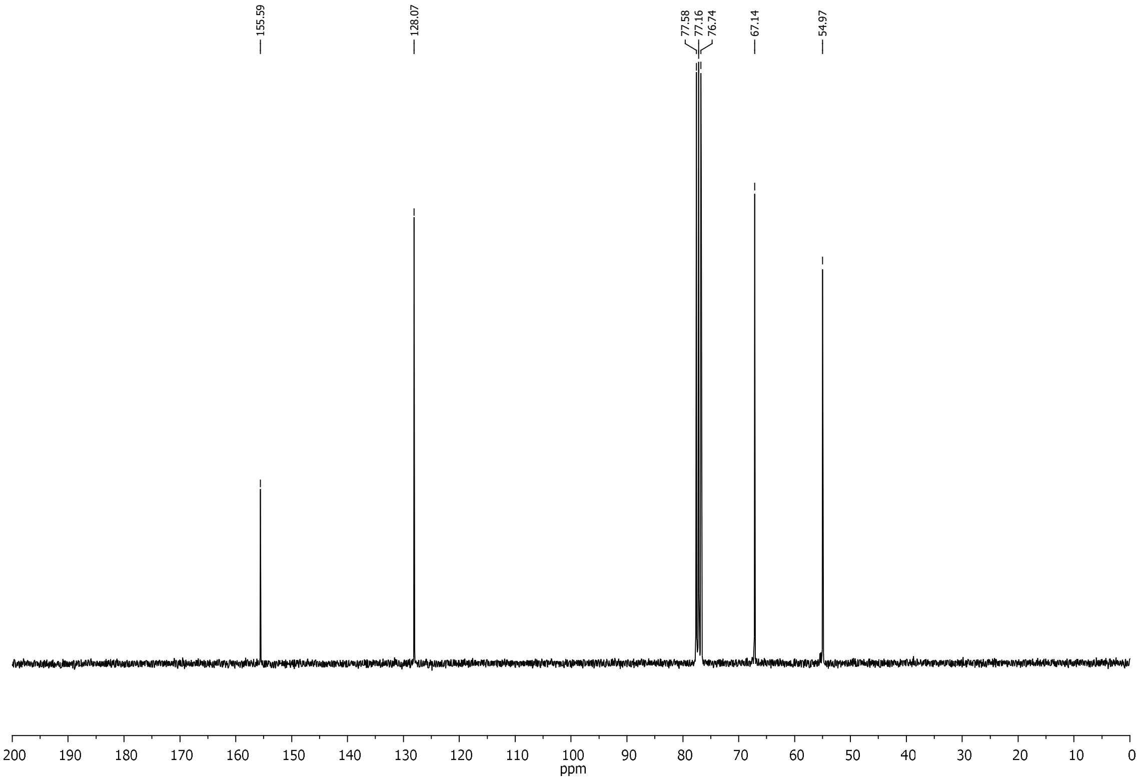

Compound **Rb**: ^{1}H-NMR (300 MHz, $CDCl_3$); ^{13}C-NMR (76 MHz, $CDCl_3$)

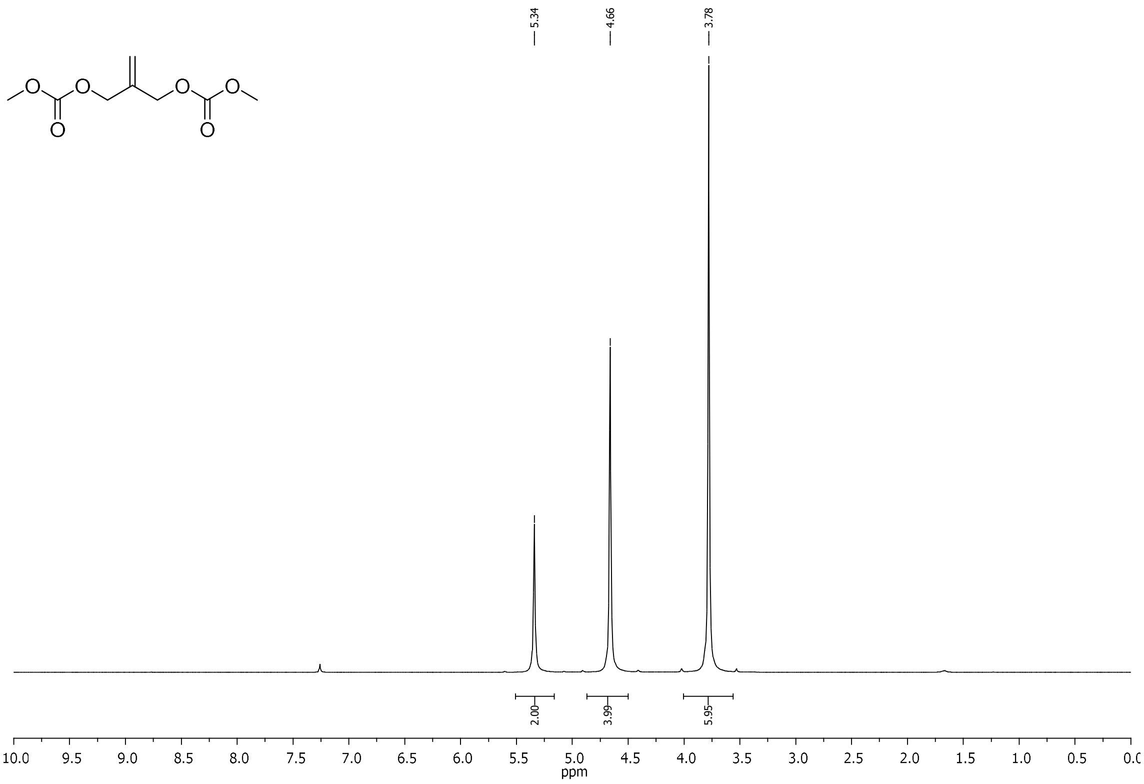

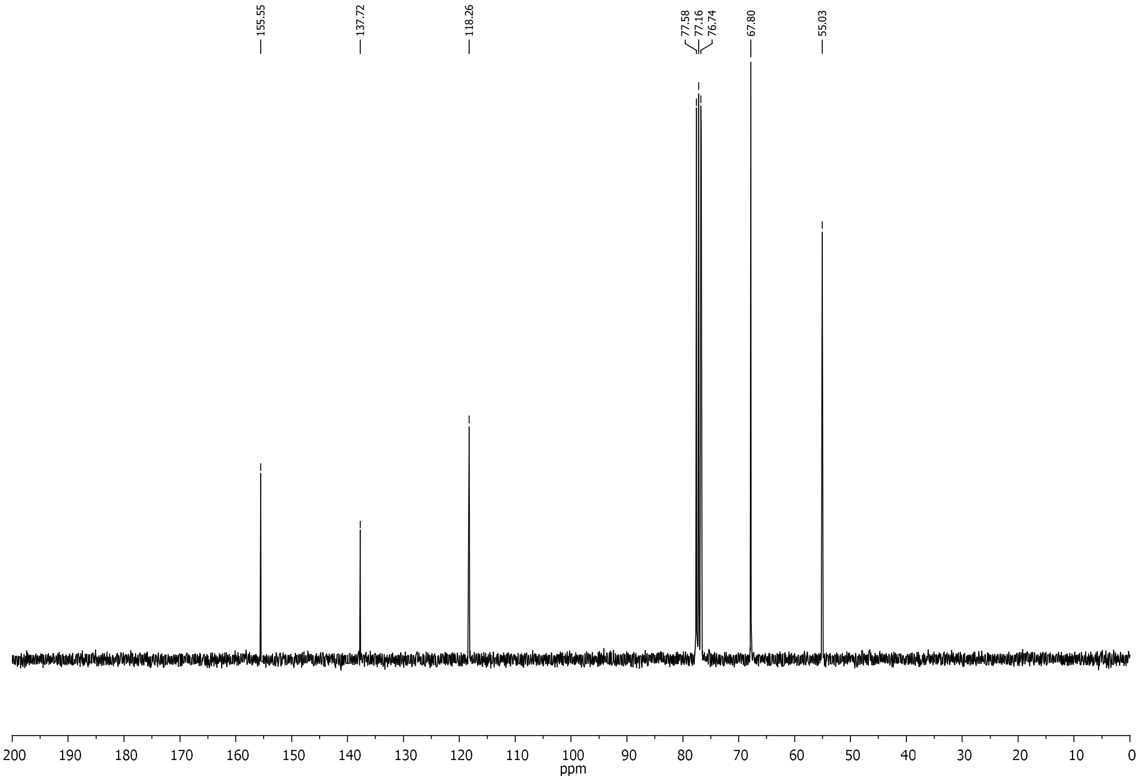

Compound **Rc**: ^{1}H-NMR (300 MHz, $CDCl_3$); ^{13}C-NMR (76 MHz, $CDCl_3$)

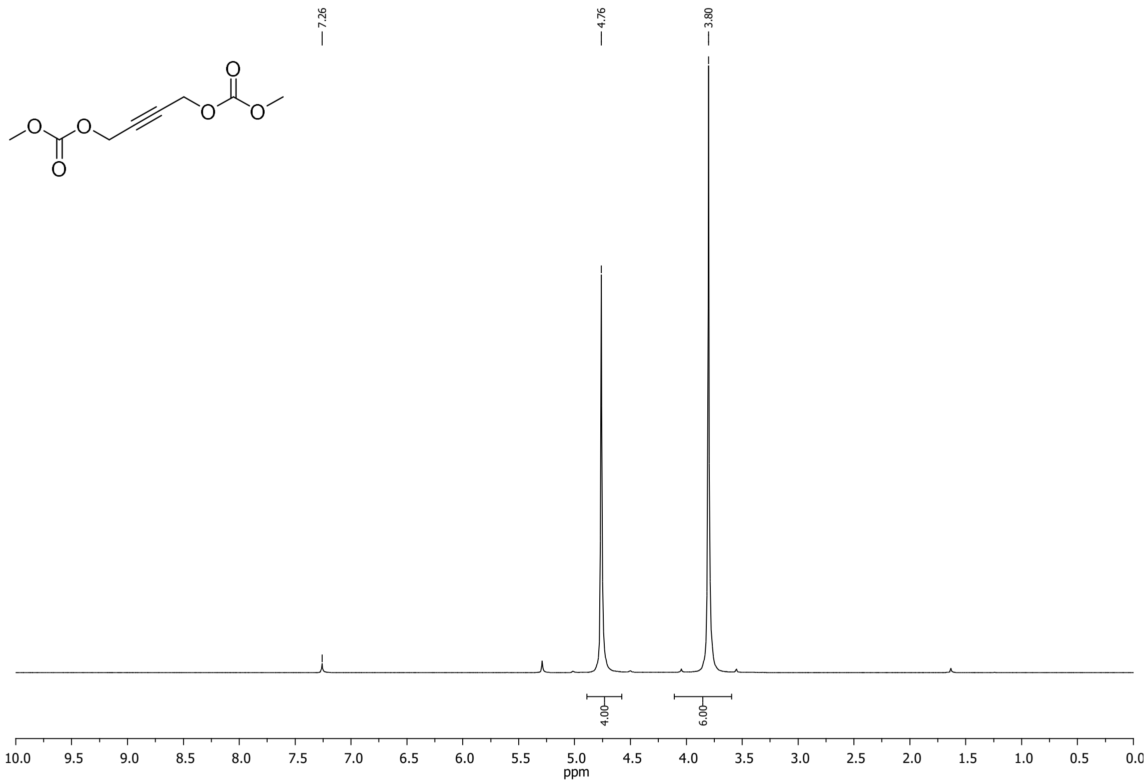

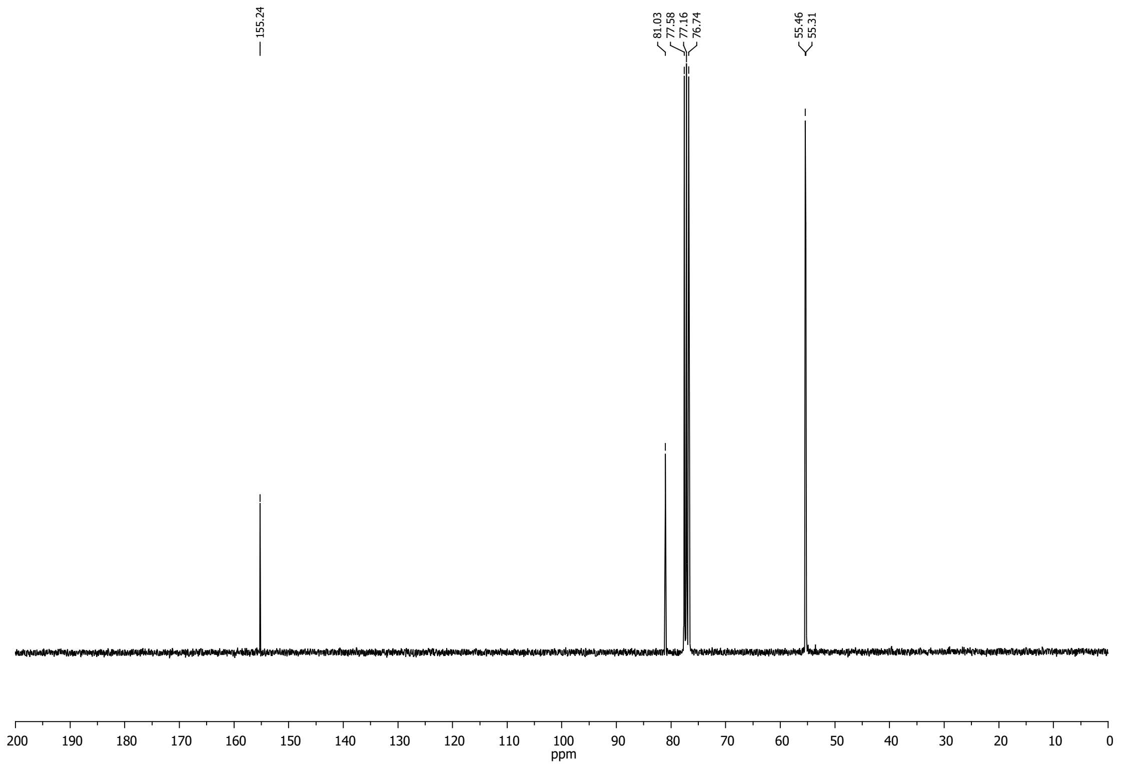

Compound **Rd**: ^{1}H-NMR (300 MHz, $CDCl_3$); ^{13}C-NMR (76 MHz, $CDCl_3$)

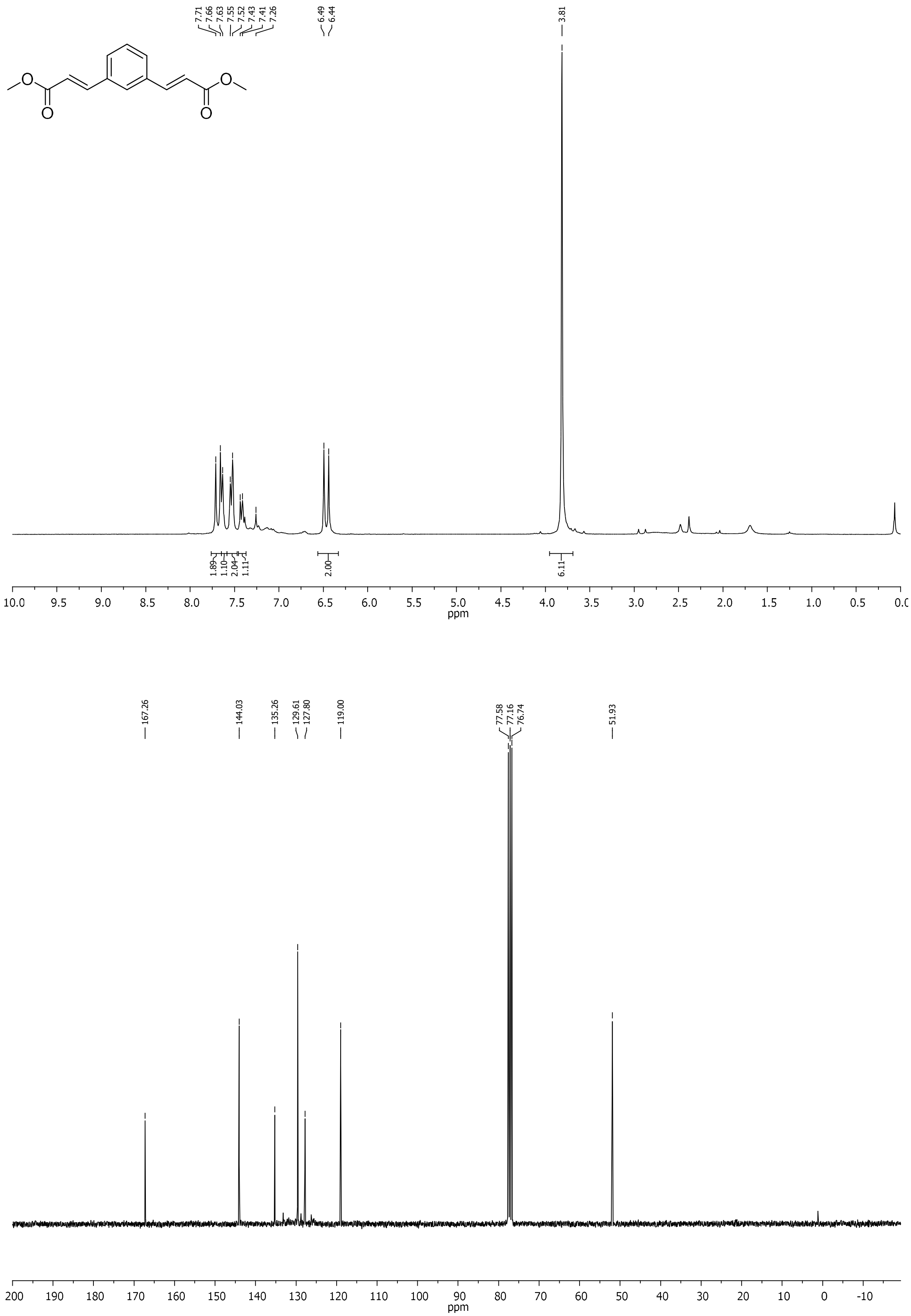

Compound **1**: ^{1}H-NMR (300 MHz, $CDCl_3$); ^{13}C-NMR (76 MHz, $CDCl_3$)

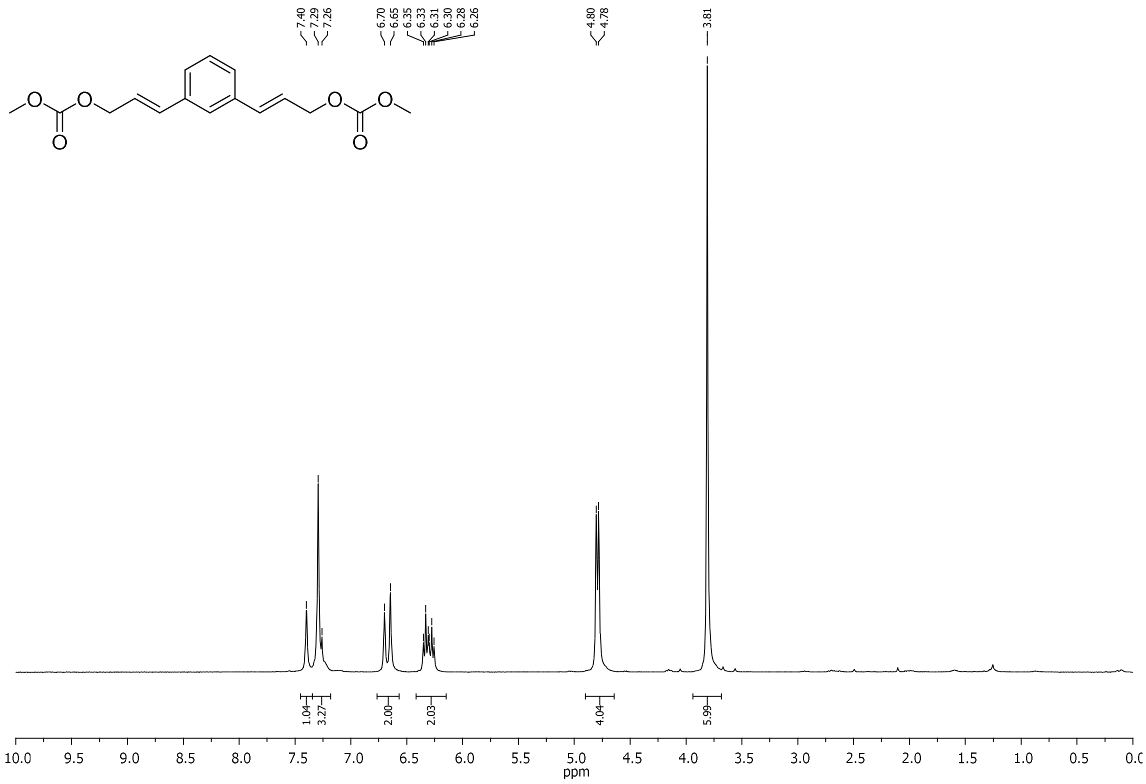

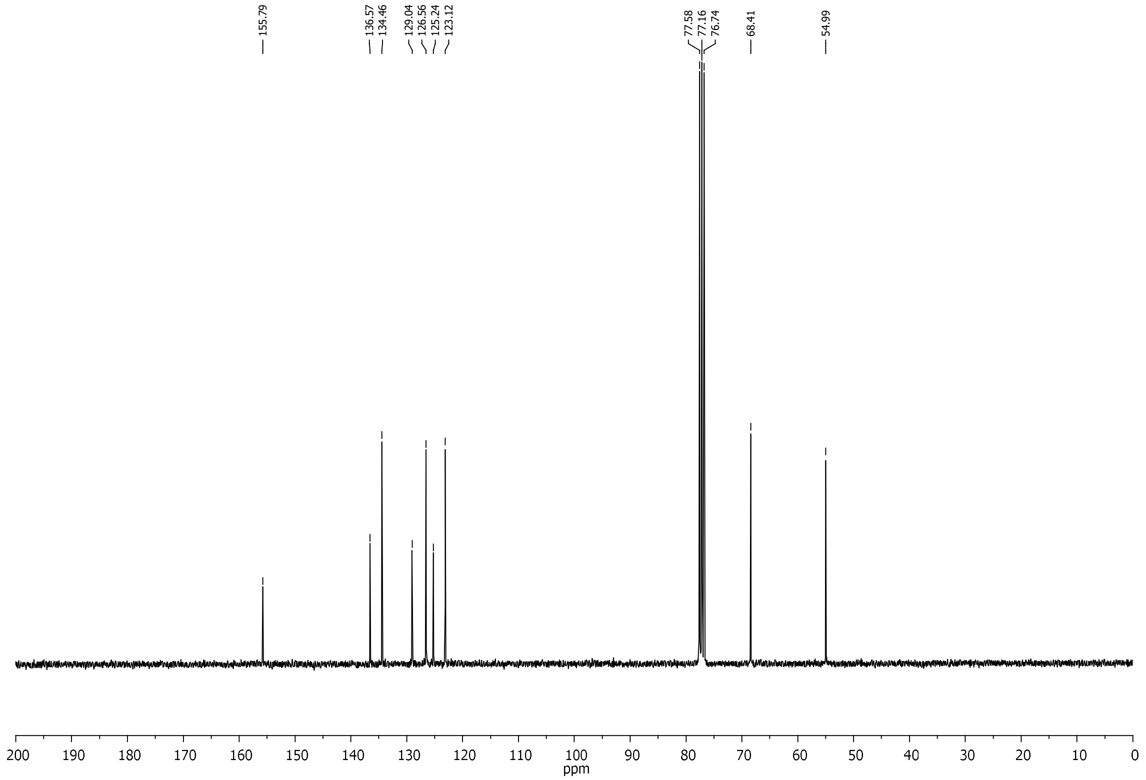

Compound **Re**: ^{1}H-NMR (300 MHz, $CDCl_3$); ^{13}C-NMR (76 MHz, $CDCl_3$)

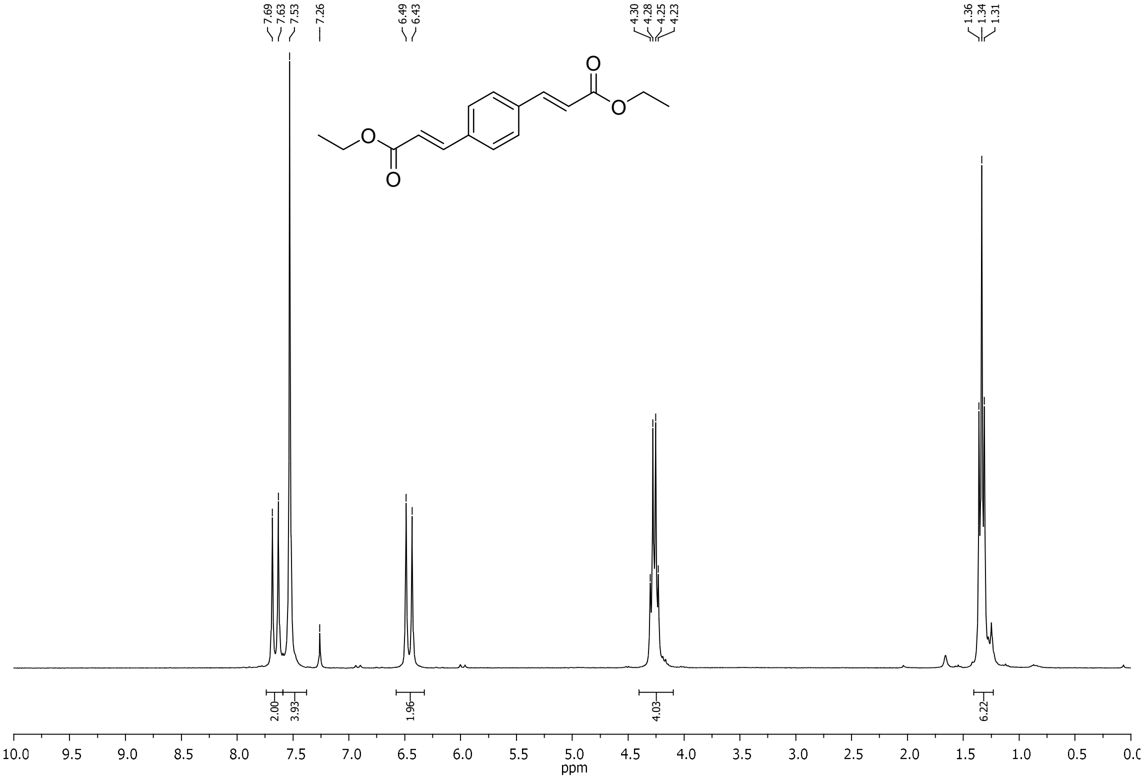

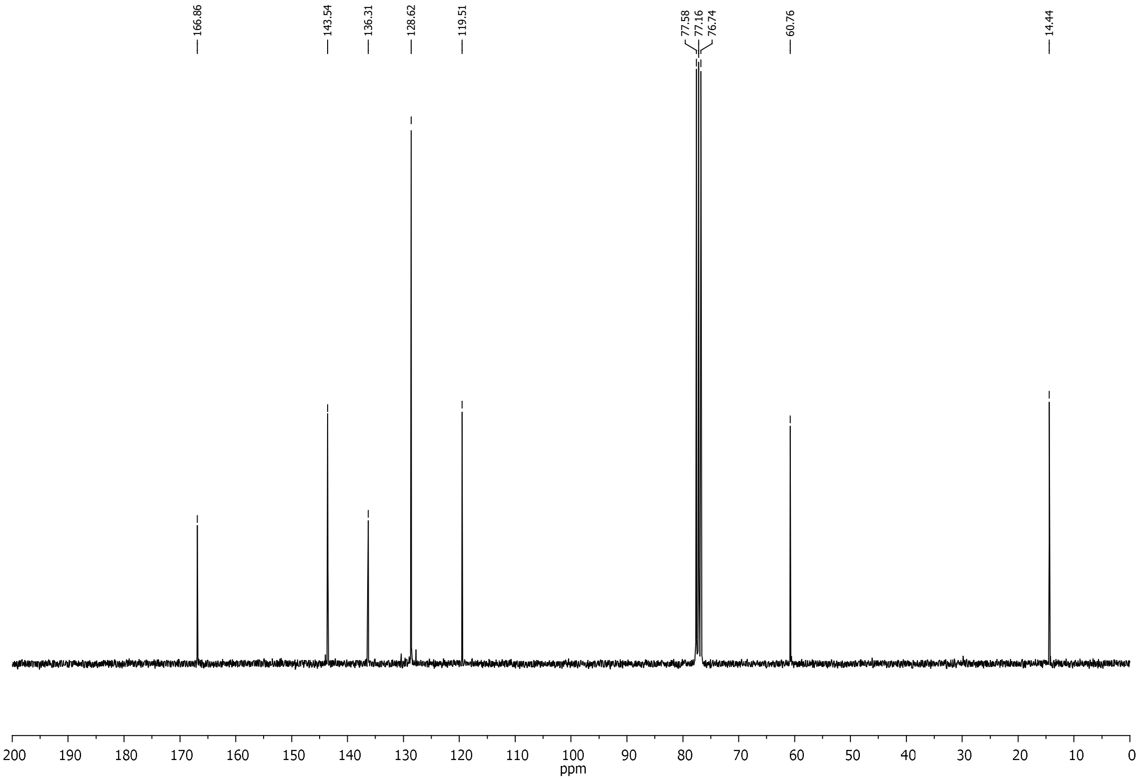

Compound **2**: ^{1}H-NMR (300 MHz, $CDCl_3$); ^{13}C-NMR (76 MHz, $CDCl_3$)

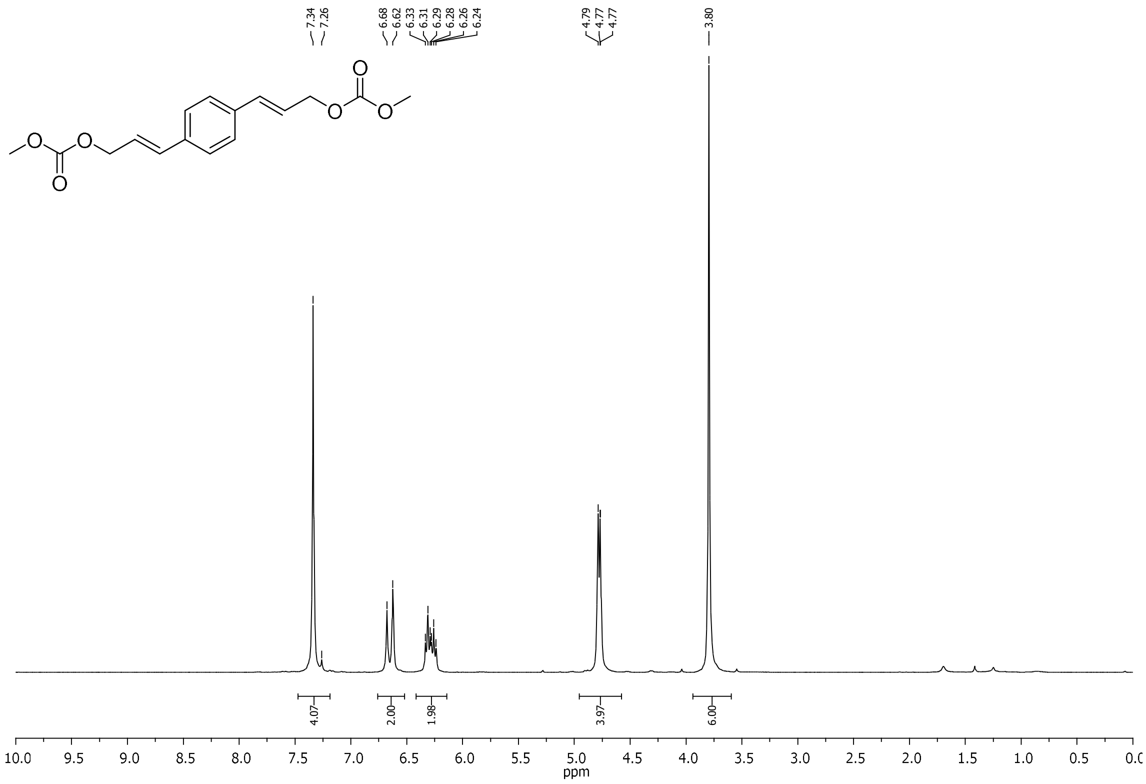

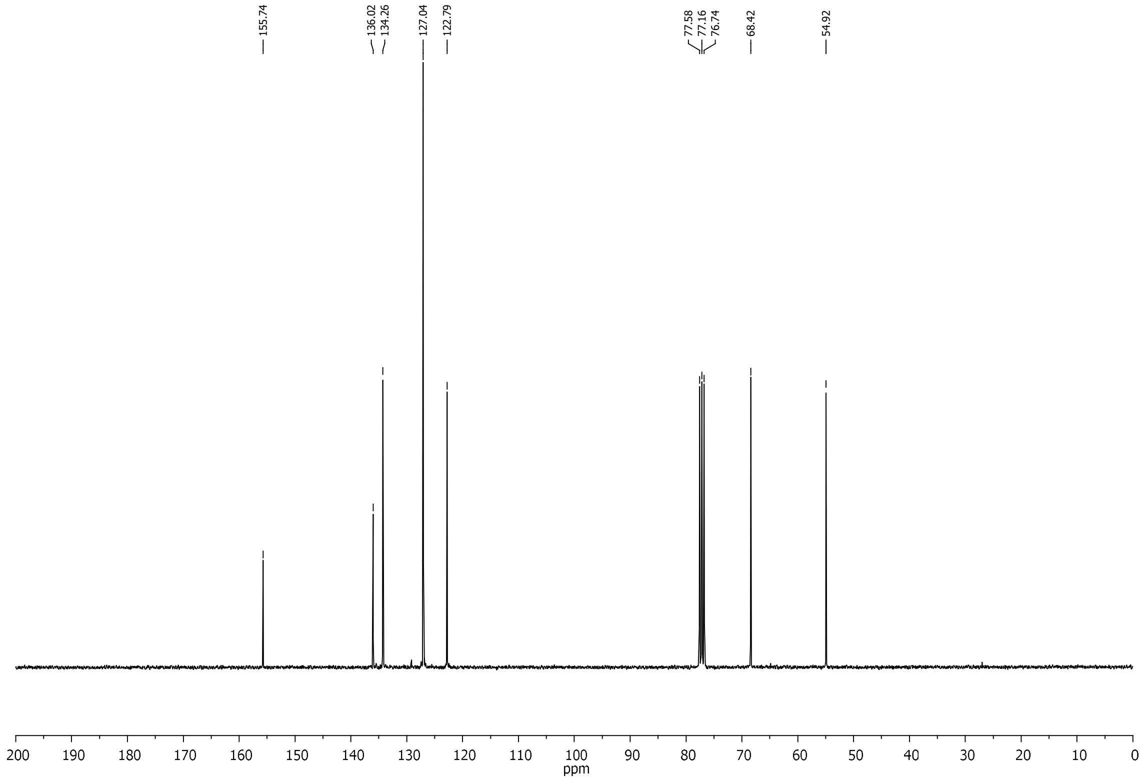

Compound **Rf**: ^{1}H-NMR (300 MHz, $CDCl_3$); ^{13}C-NMR (76 MHz, $CDCl_3$)

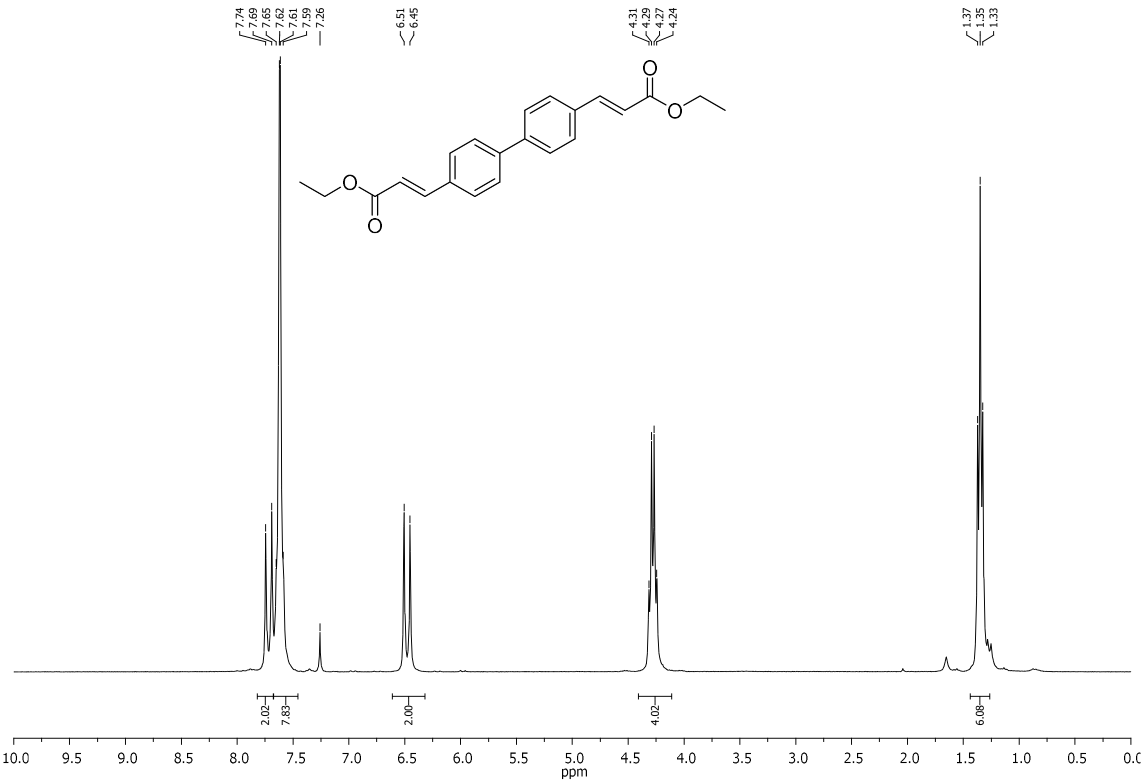

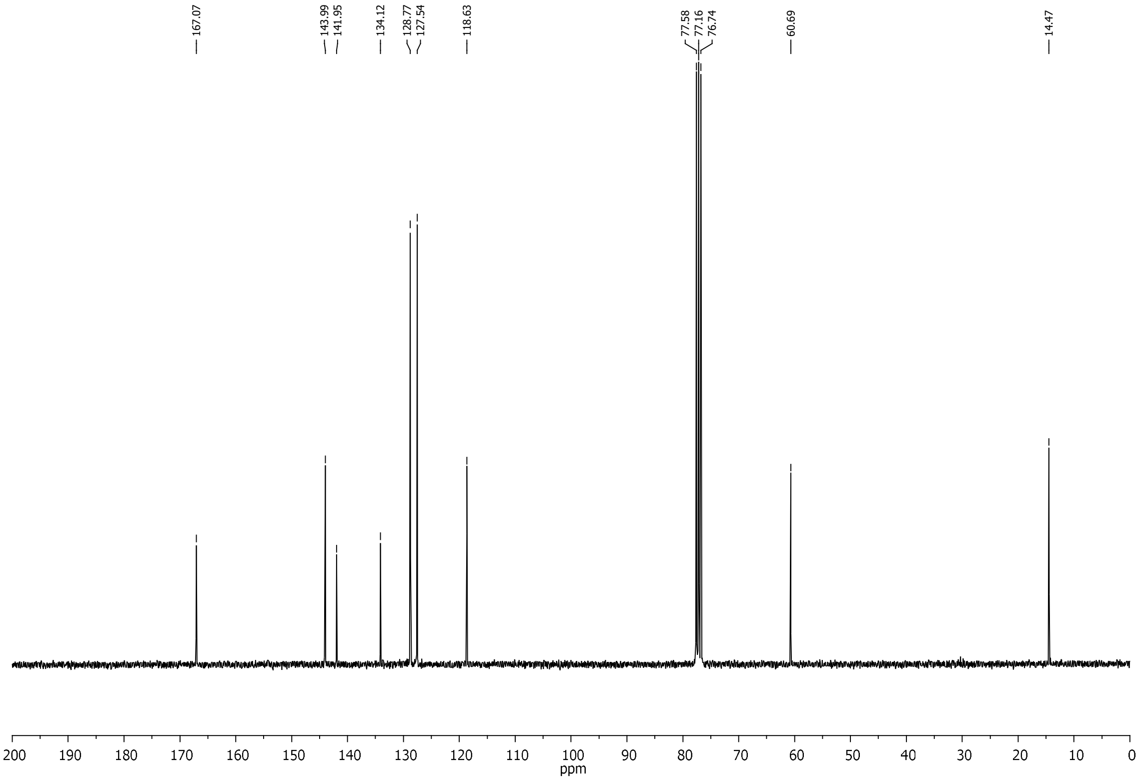

Compound **3**: ^{1}H-NMR (300 MHz, $CDCl_3$); ^{13}C-NMR (76 MHz, $CDCl_3$)

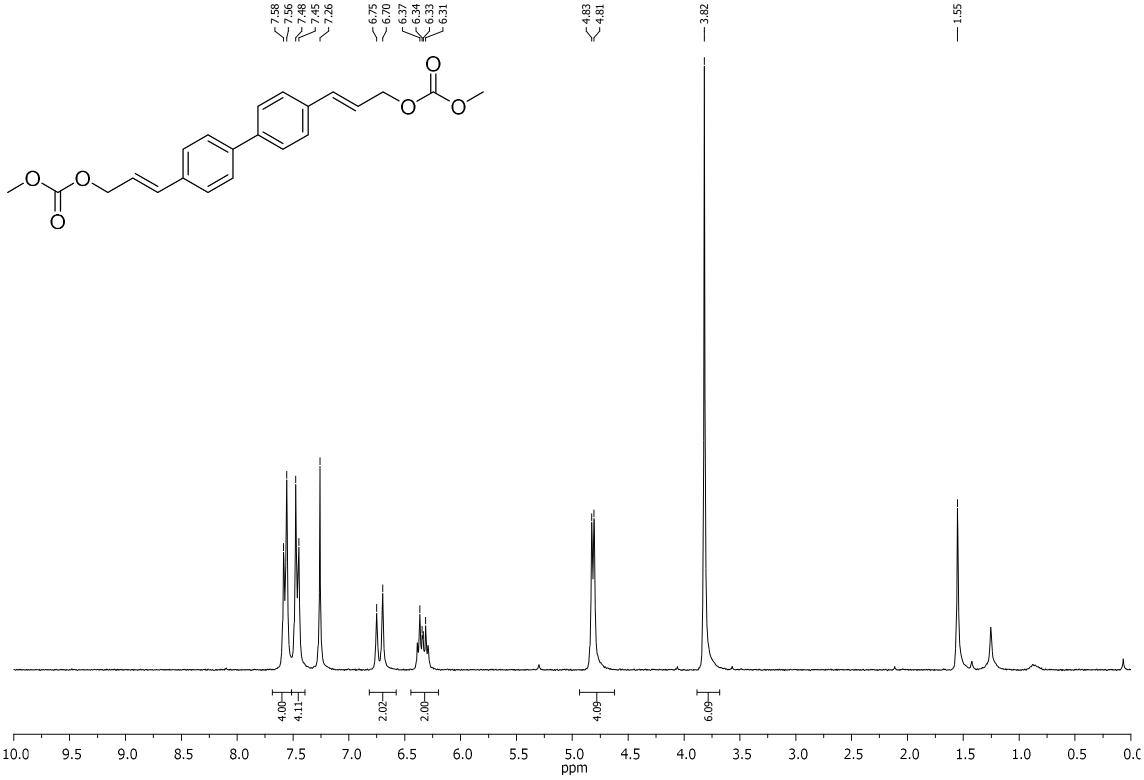

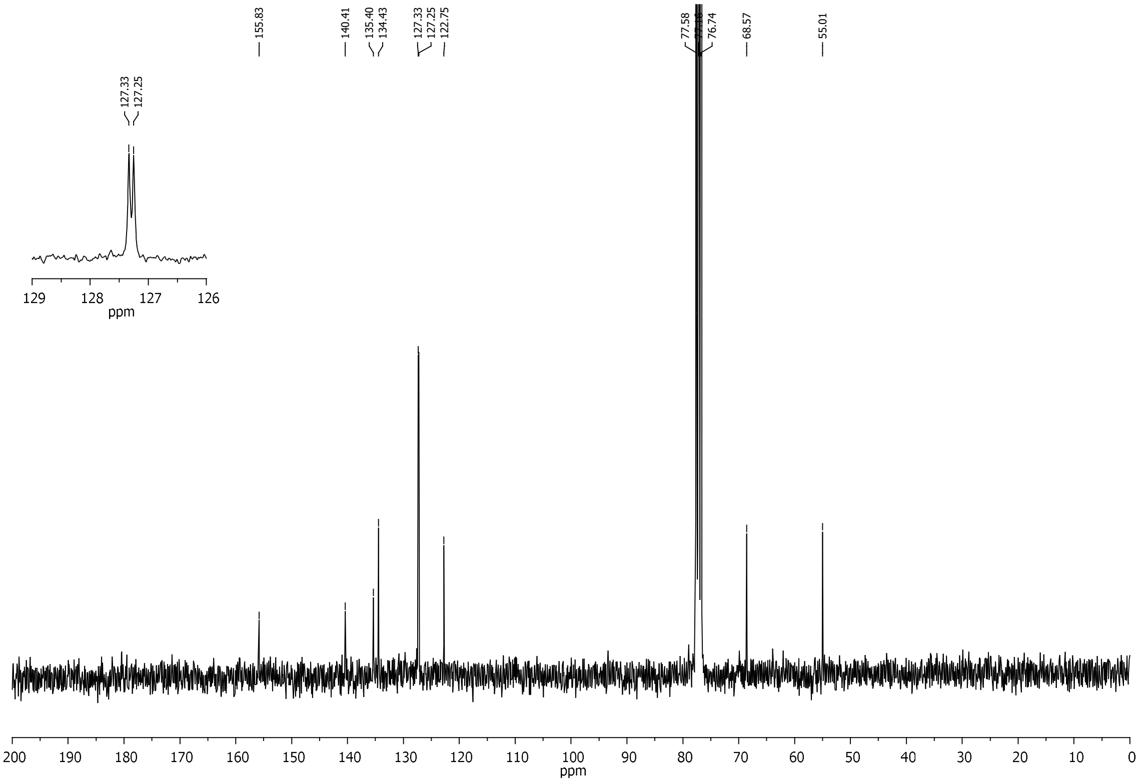

Compound **Rg**: ^{1}H-NMR (300 MHz, $CDCl_3$); ^{13}C-NMR (76 MHz, $CDCl_3$)

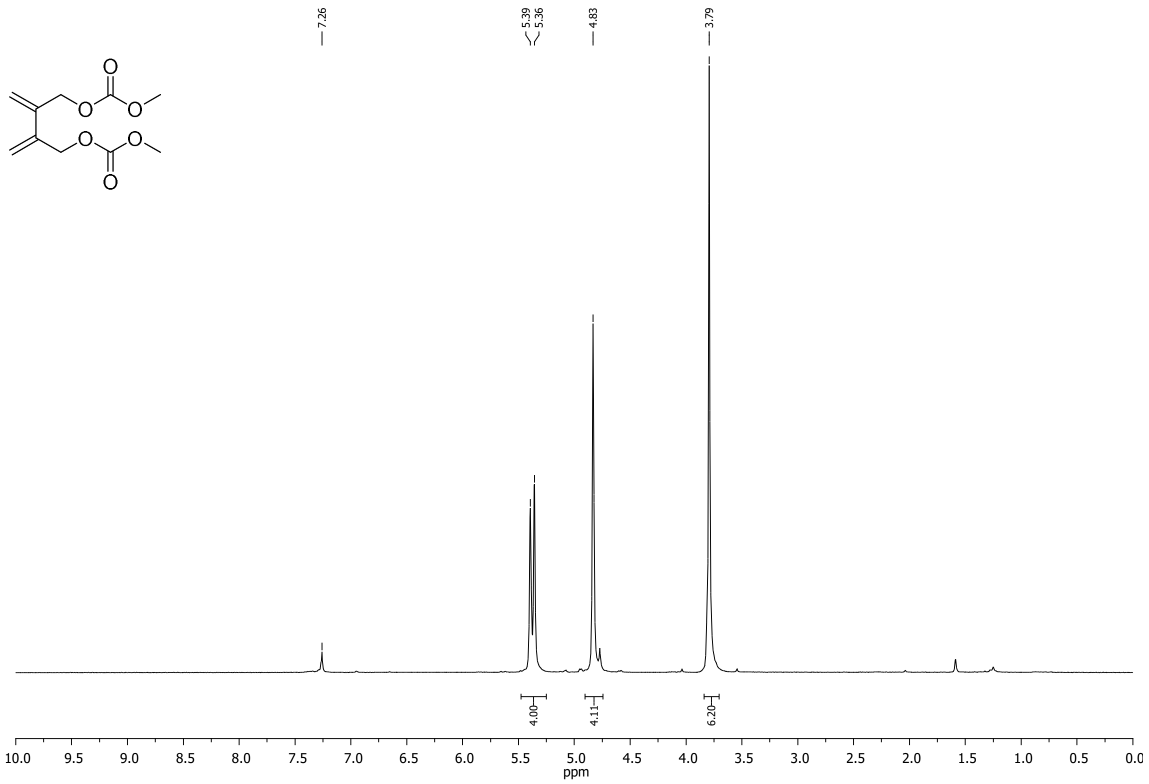

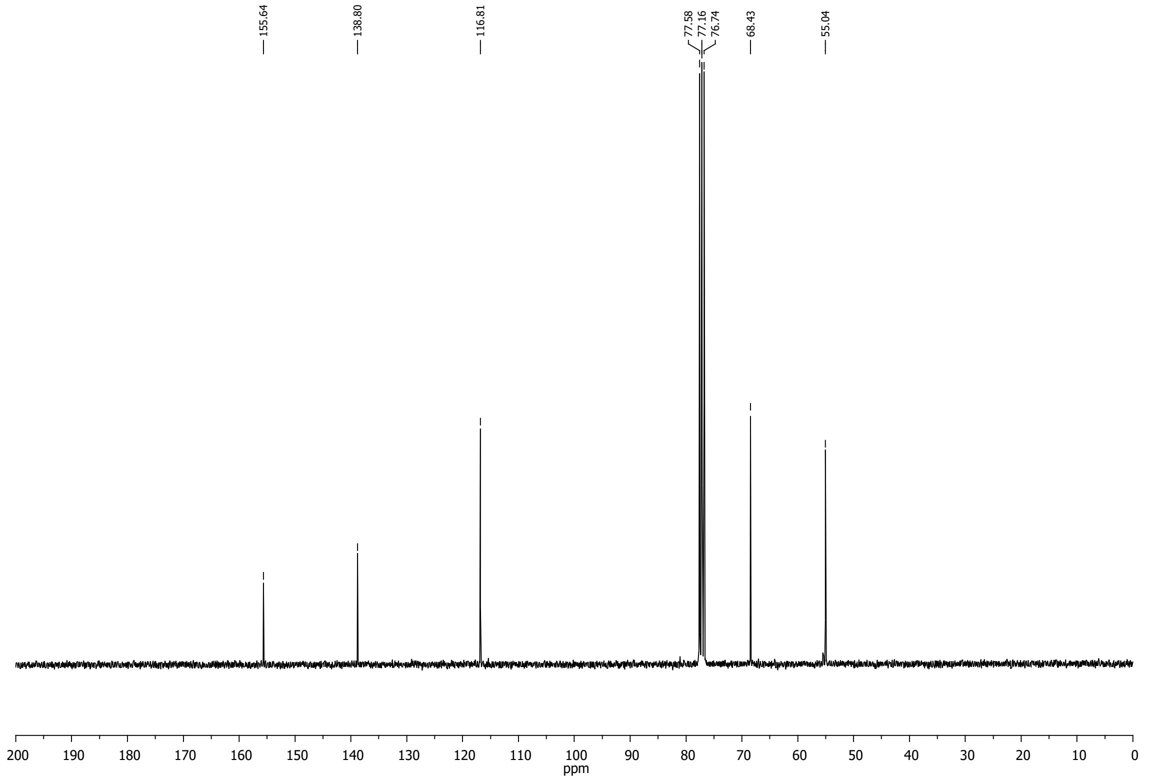

Compound **Rh**: ^{1}H-NMR (300 MHz, $CDCl_3$); ^{13}C-NMR (76 MHz, $CDCl_3$)

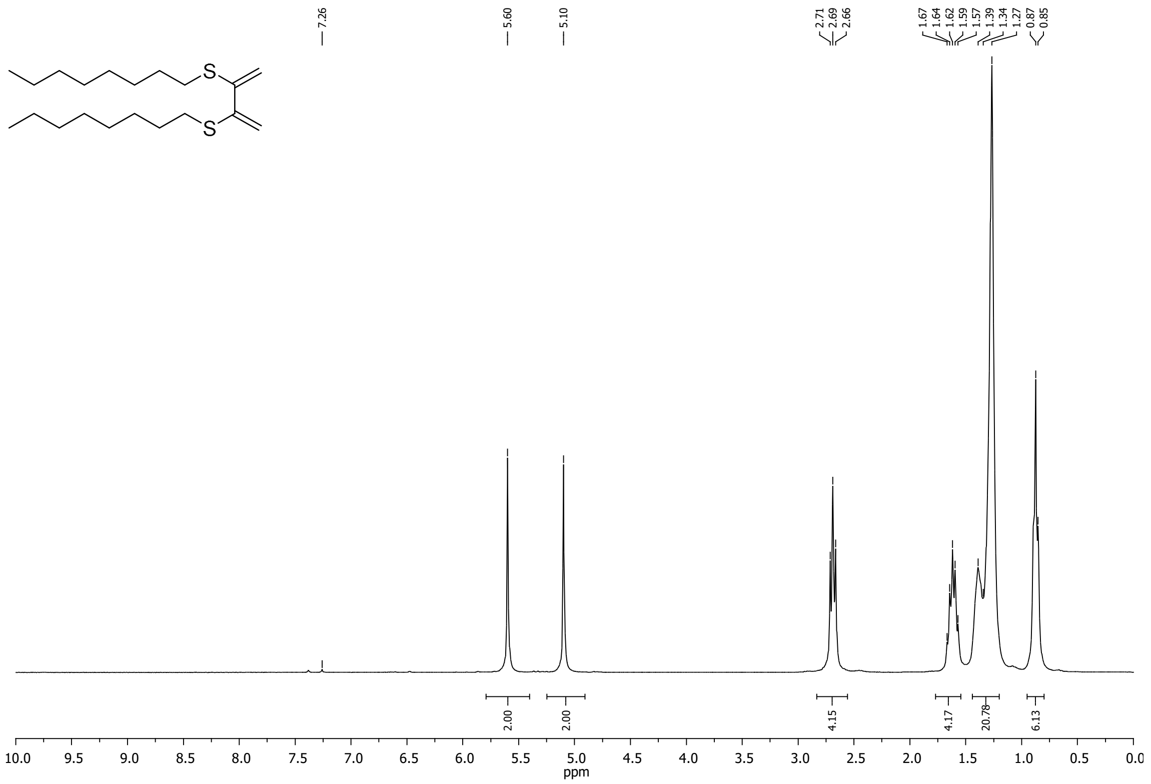

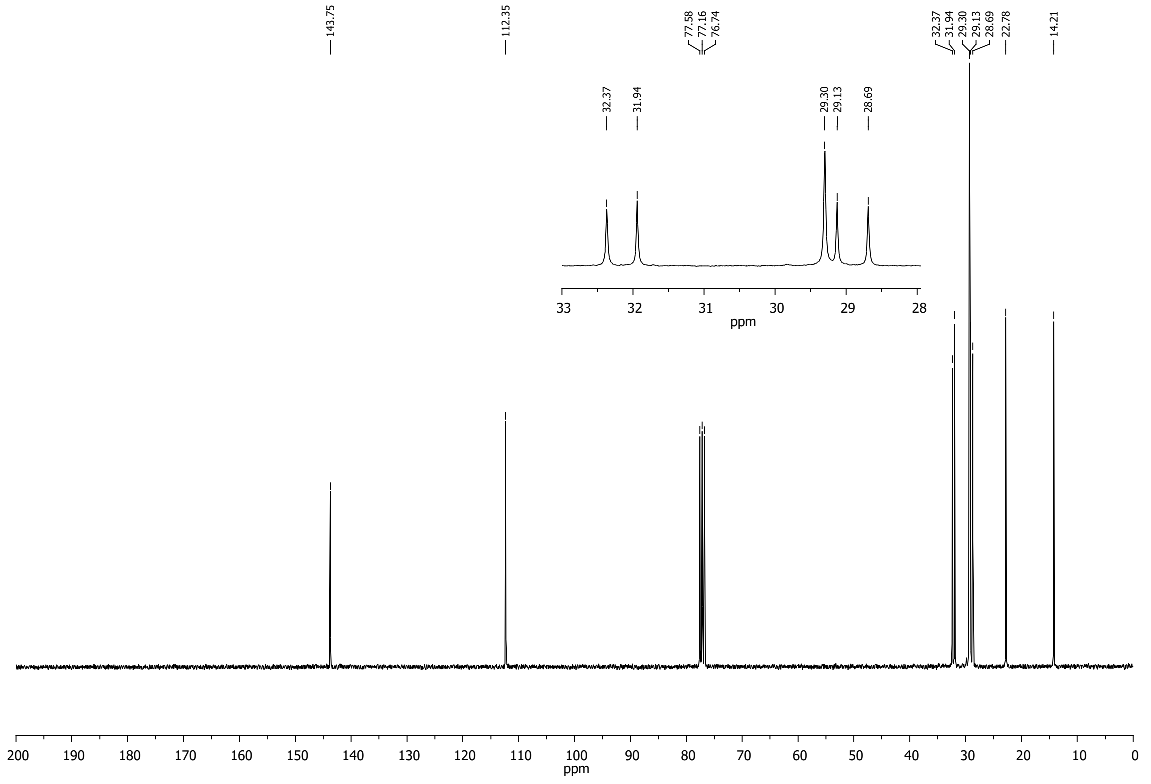

Compound **4**: ^{1}H-NMR (300 MHz, $CDCl_3$); ^{13}C-NMR (76 MHz, $CDCl_3$)

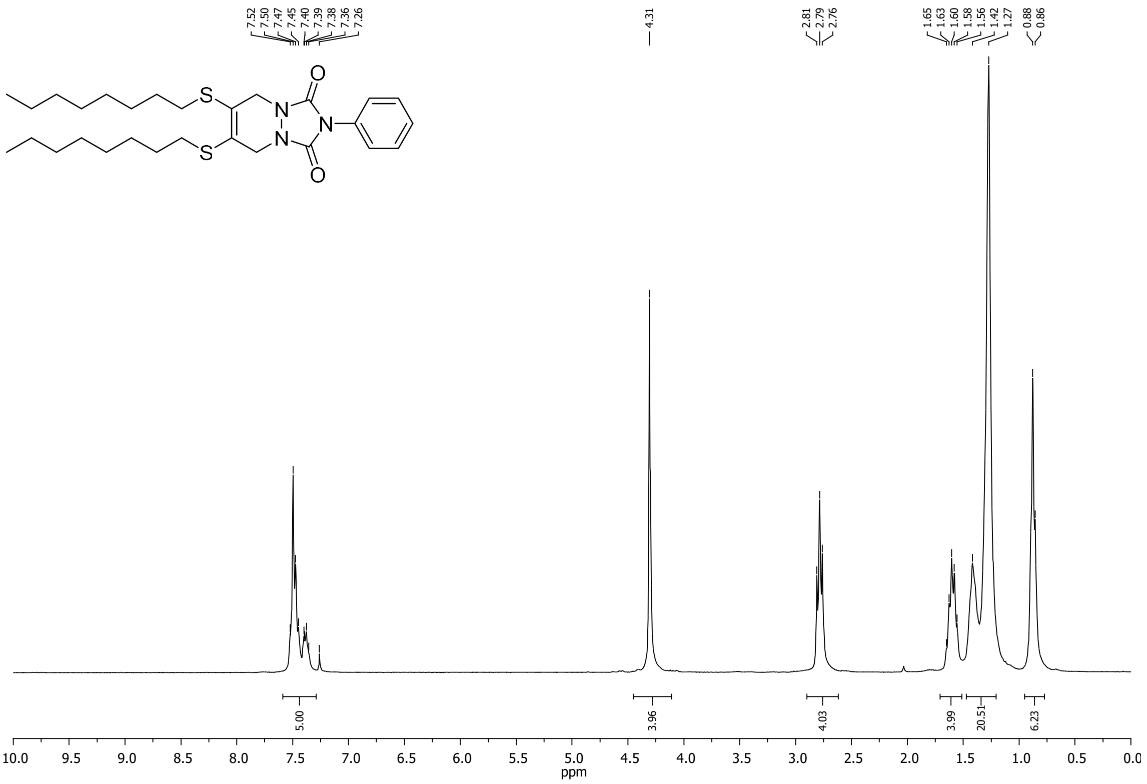

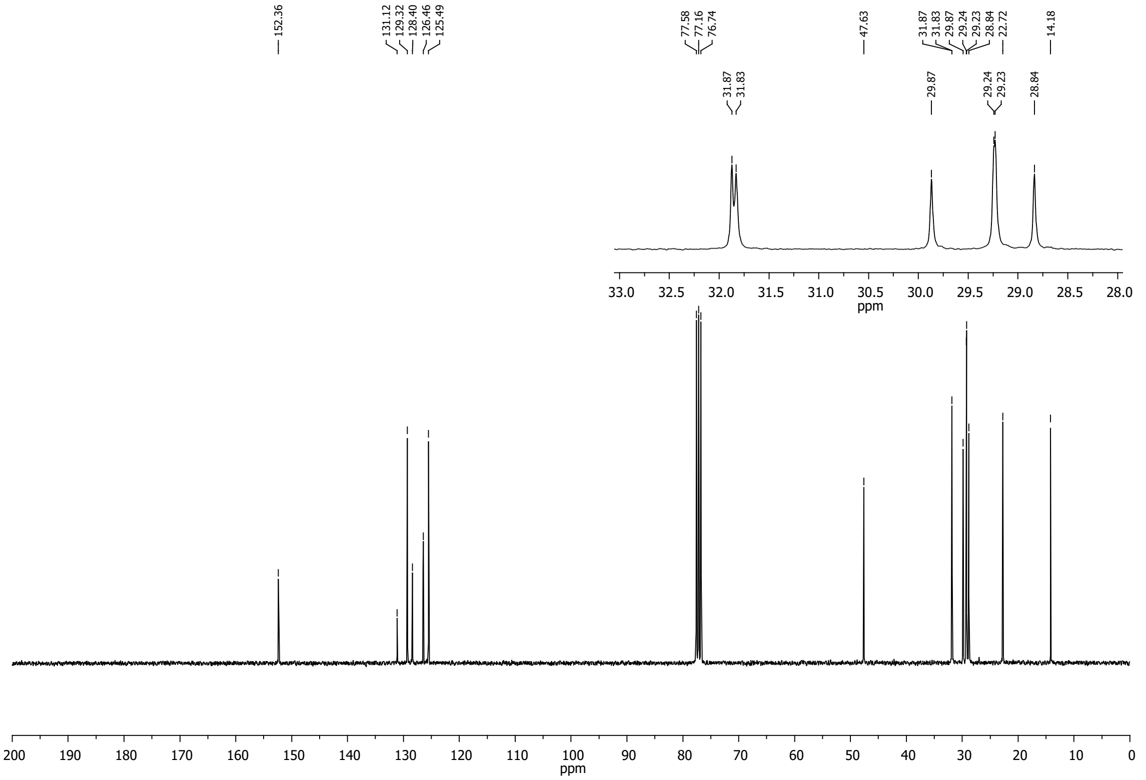

Compound **5**: ^{1}H-NMR (300 MHz, $CDCl_3$); ^{13}C-NMR (76 MHz, $CDCl_3$)

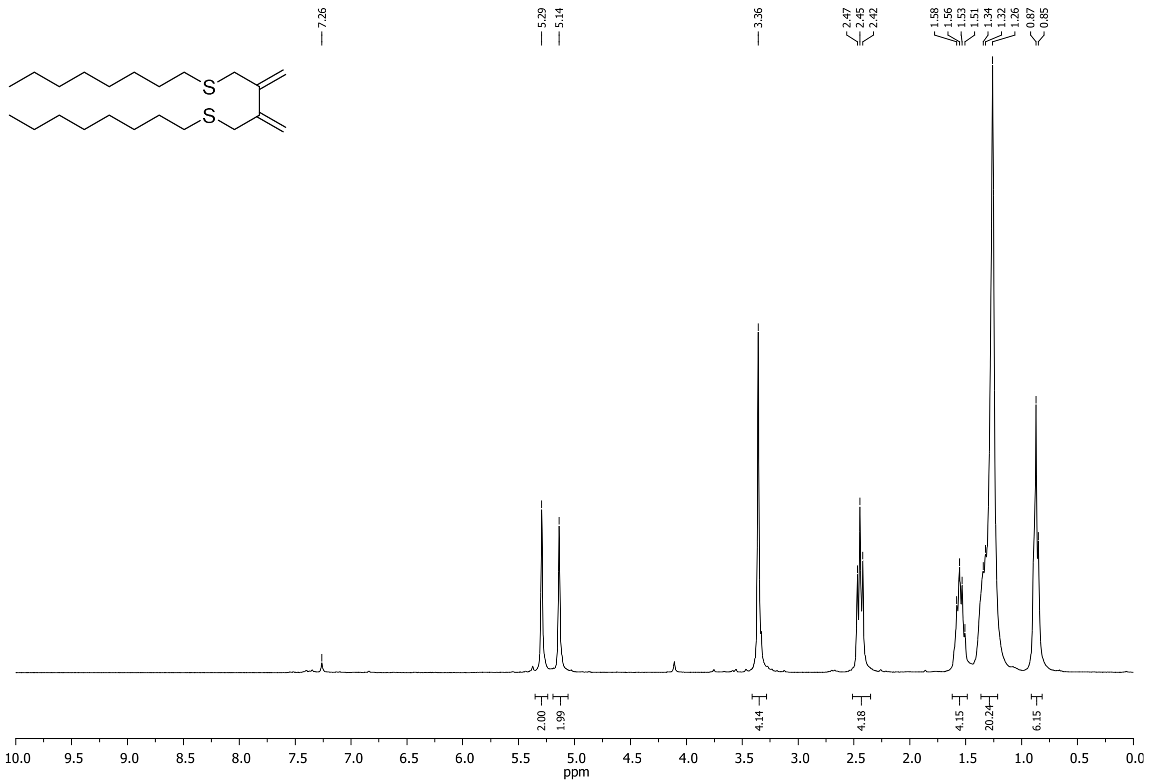

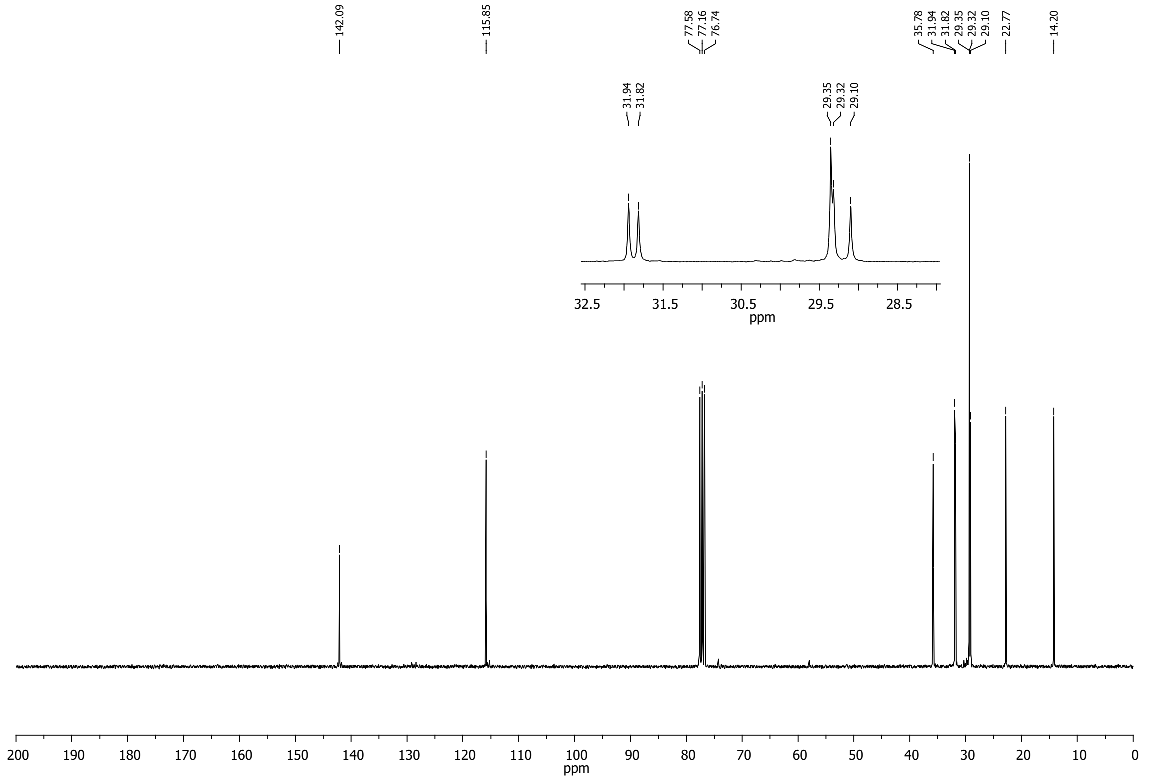

Compound **6**: ^{1}H-NMR (300 MHz, $CDCl_3$); ^{13}C-NMR (76 MHz, $CDCl_3$)

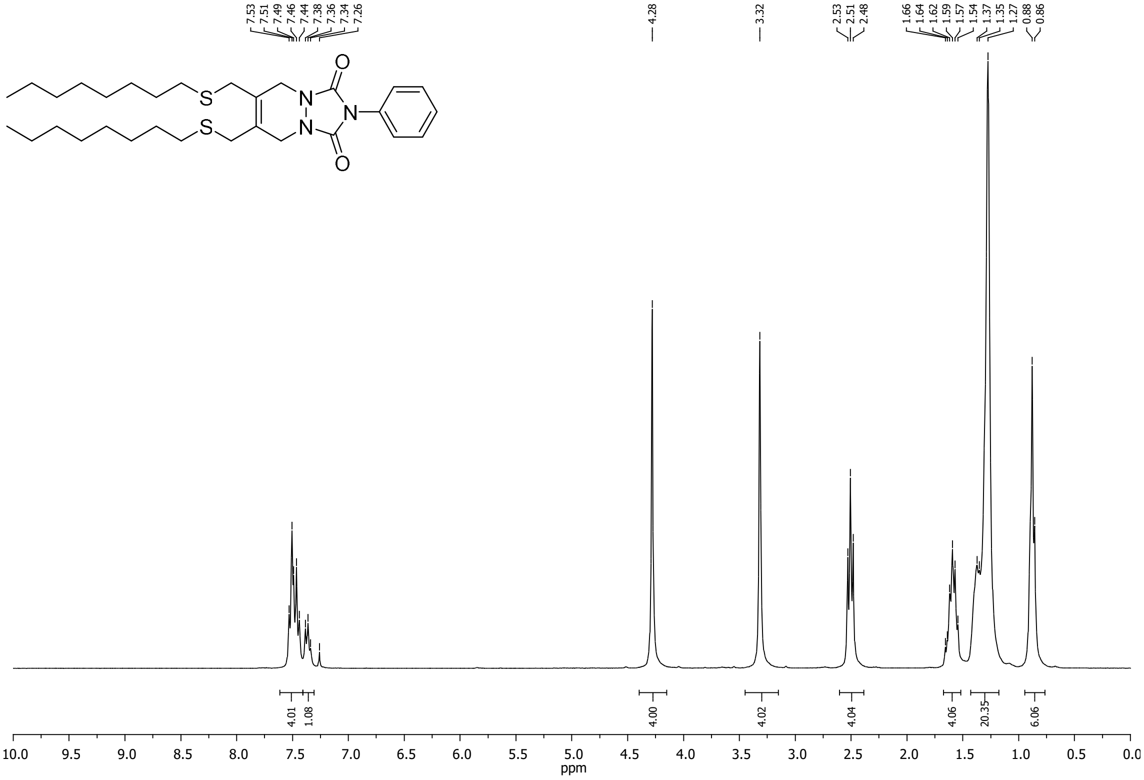

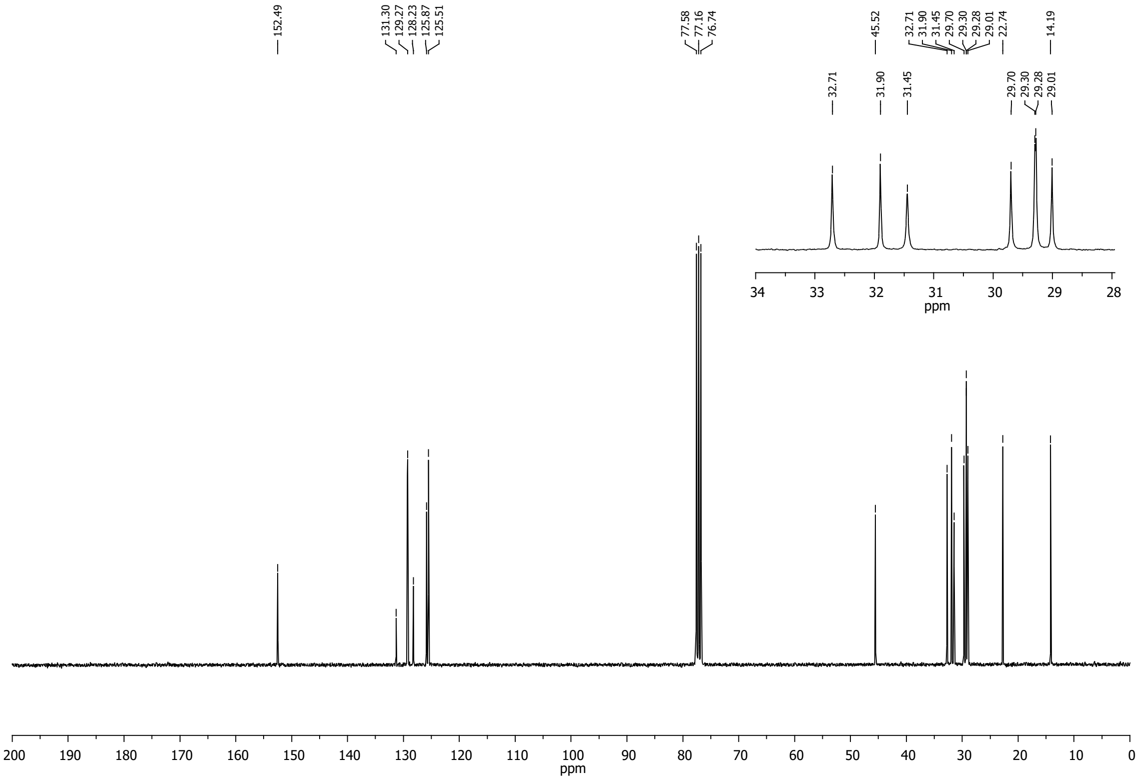

Compound **7**: ^{1}H-NMR (300 MHz, $CDCl_3$); ^{13}C-NMR (76 MHz, $CDCl_3$)

Analysis of the peptides

P1: cdGYGPNcGGK(PEG-Biotin)-NH_2

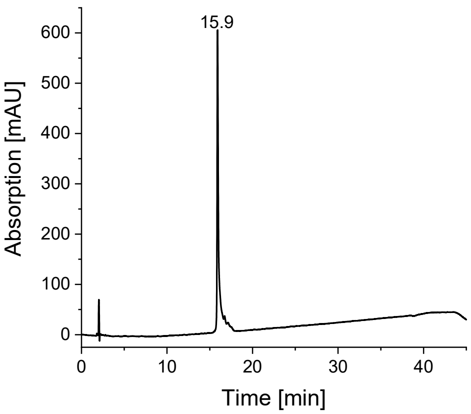

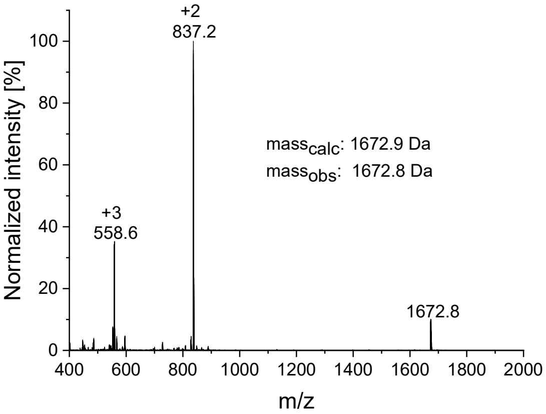

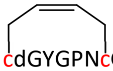

P1a: cdGYGPNcGGK(PEG-Biotin)-NH_2

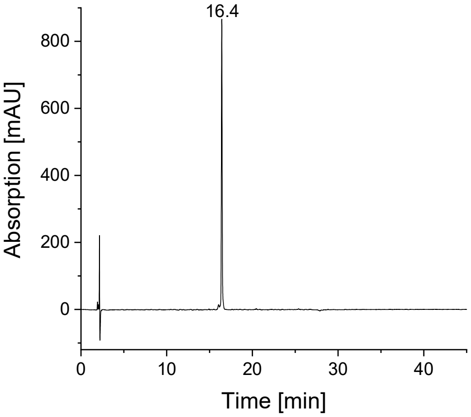

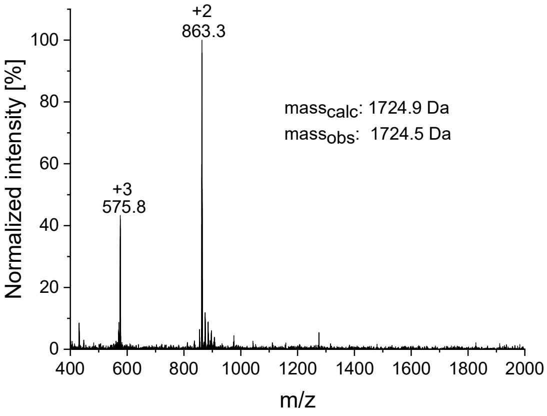

P1b: 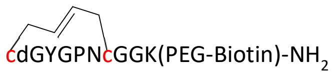
cdGYGPNcGGK(PEG-Biotin)-NH$_2$

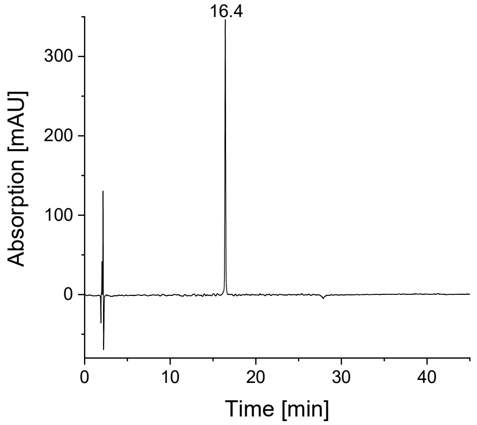
16.4
Absorption [mAU]
Time [min]

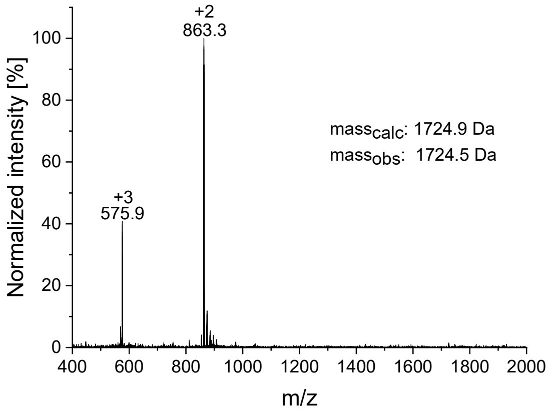
+2
863.3
+3
575.9
mass$_{calc}$: 1724.9 Da
mass$_{obs}$: 1724.5 Da
Normalized intensity [%]
m/z

P1c: 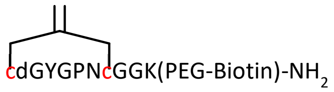
cdGYGPNcGGK(PEG-Biotin)-NH$_2$

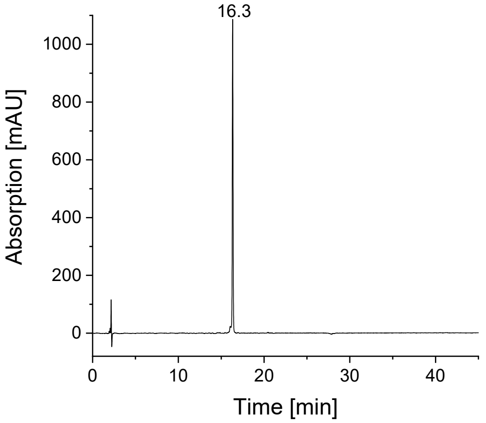
16.3
Absorption [mAU]
Time [min]

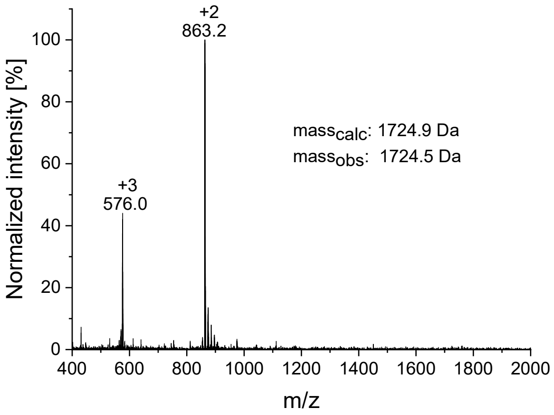
+2
863.2
+3
576.0
mass$_{calc}$: 1724.9 Da
mass$_{obs}$: 1724.5 Da
Normalized intensity [%]
m/z

P1d: 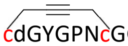
cdGYGPNcGGK(PEG-Biotin)-NH_2

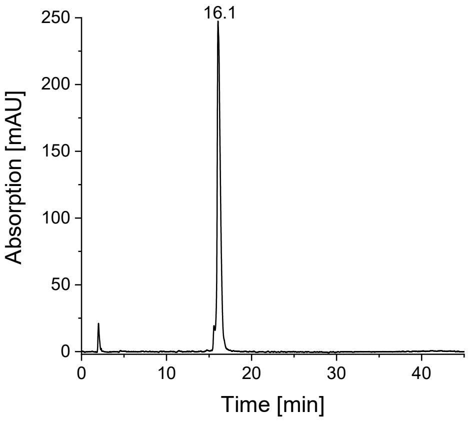

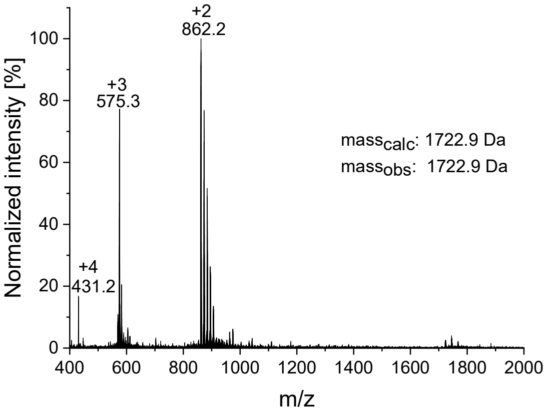

P1e: 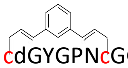
cdGYGPNcGGK(PEG-Biotin)-NH_2

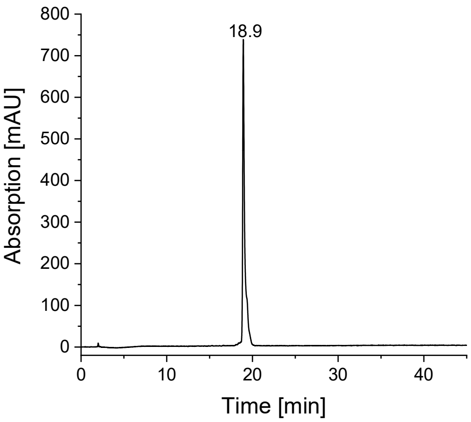

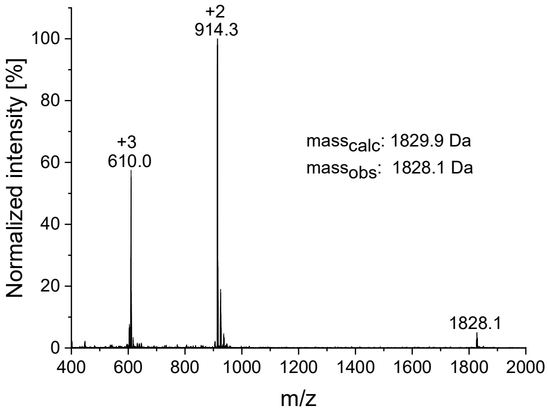

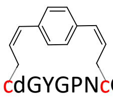

P1f: cdGYGPNcGGK(PEG-Biotin)-NH_2

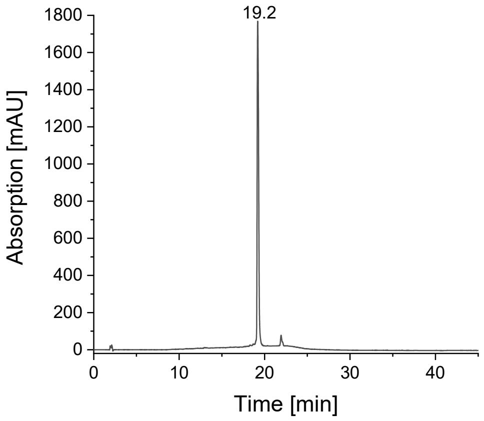

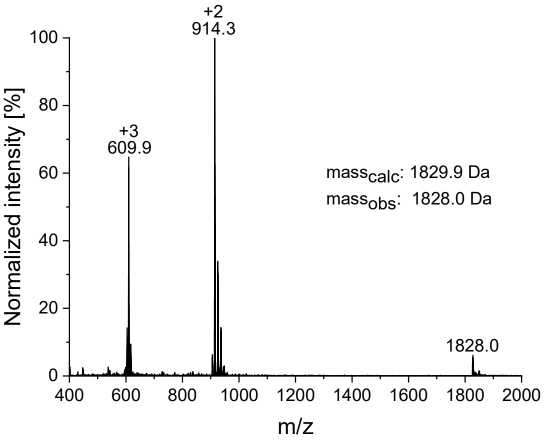

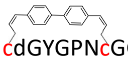

P1g: cdGYGPNcGGK(PEG-Biotin)-NH_2

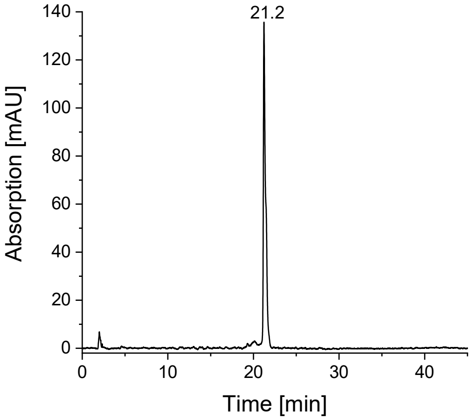

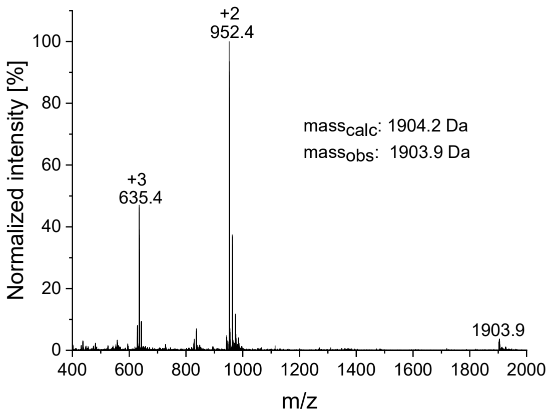

P3: EWACTACAKFLAAHA

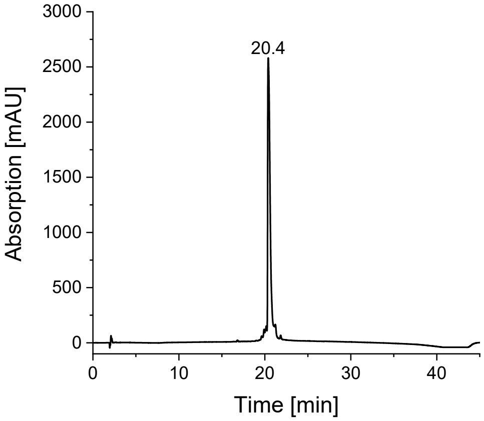

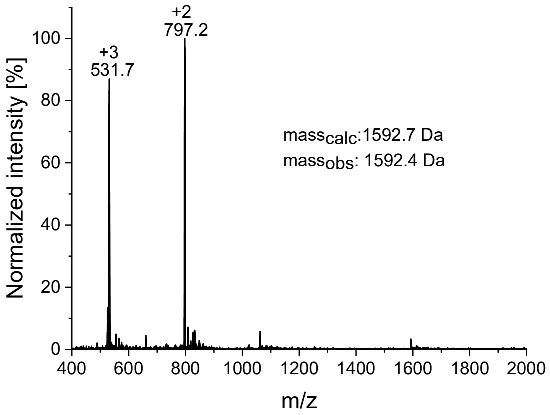

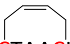

P3a: EWACTAACKFLAAHA

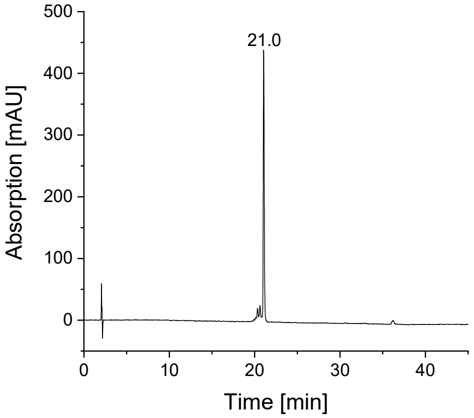

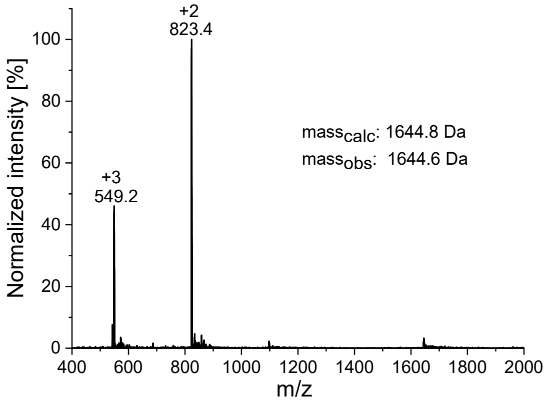

P3c: EWACTAACKFLAAHA

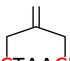

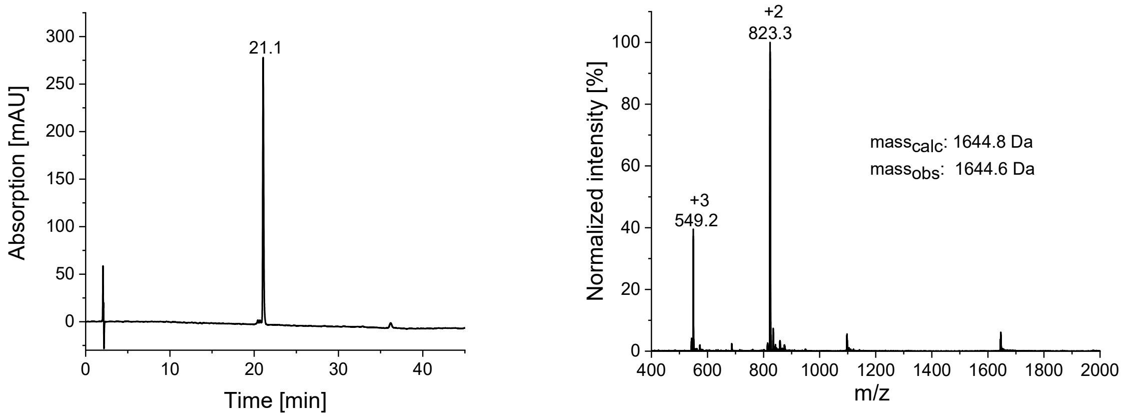

P3h: EWACTACAKFLAAHA

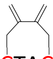

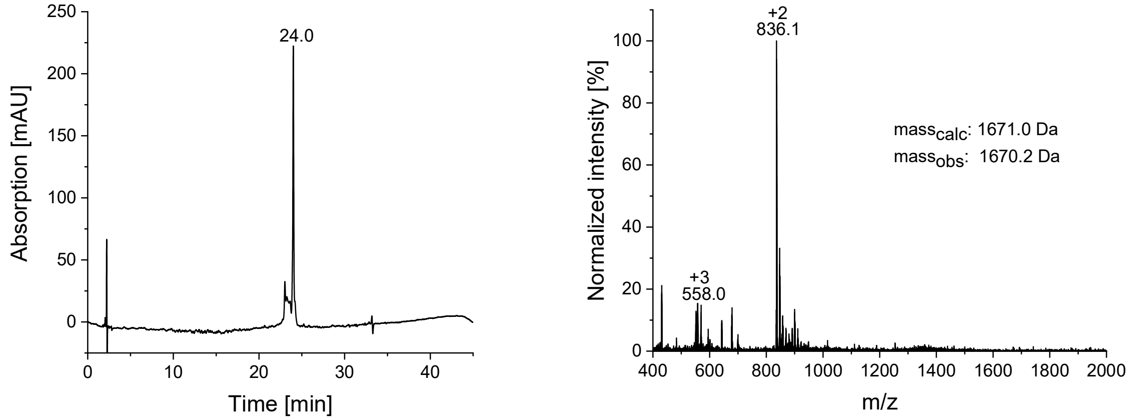

P4: AGCKNFFWKTFTSC

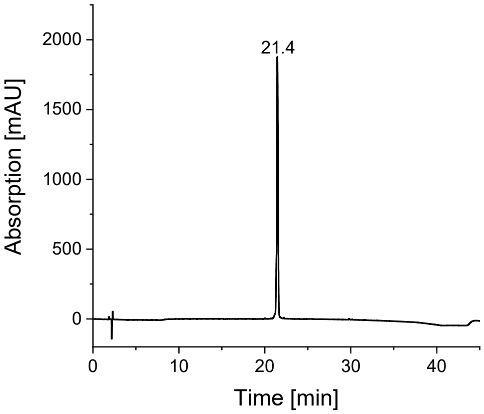

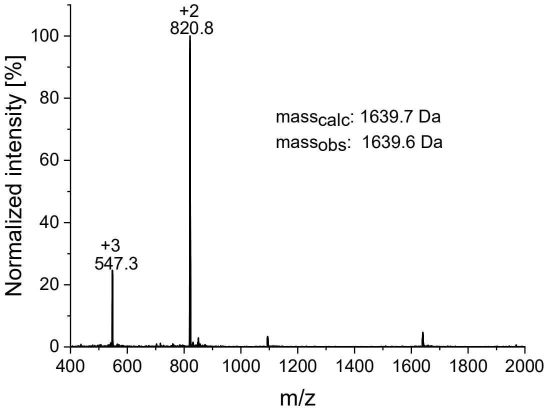

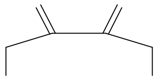

P4h: AGCKNFFWKTFTSC

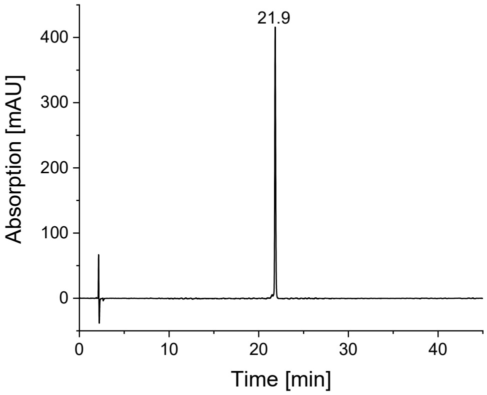

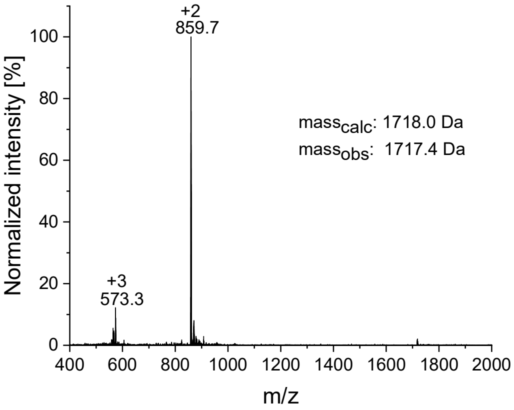

References

(1) Schlatzer, T.; Kriegesmann, J.; Schröder, H.; Trobe, M.; Lembacher-Fadum, C.; Santner, S.; Kravchuk, A. V.; Becker, C. F. W.; Breinbauer, R. Labeling and Natural Post-Translational Modification of Peptides and Proteins via Chemoselective Pd-Catalyzed Prenylation of Cysteine. *J Am Chem Soc* **2019**, *141* (37), 14931–14937. https://doi.org/10.1021/jacs.9b08279.

(2) Budavari, S.; O'Neil, M.; Smith, A.; Heckelman, P. E. The Merck Index : An Encyclopedia of Chemicals, Drugs, and Biologicals. **1983**, *44* (10), 44-5373-44–5373. https://doi.org/10.5860/CHOICE.44-5373.

(3) Gottlieb, H. E.; Kotlyar, V.; Nudelman, A. NMR Chemical Shifts of Common Laboratory Solvents as Trace Impurities. *J Org Chem* **1997**, *62* (21), 7512–7515. https://doi.org/10.1021/JO971176V.

(4) Delaglio, F.; Grzesiek, S.; Vuister, G. W.; Zhu, G.; Pfeifer, J.; Bax, A. NMRPipe: A Multidimensional Spectral Processing System Based on UNIX Pipes. *Journal of Biomolecular NMR 1995 6:3* **1995**, *6* (3), 277–293. https://doi.org/10.1007/BF00197809.

(5) Lee, W.; Tonelli, M.; Markley, J. L. NMRFAM-SPARKY: Enhanced Software for Biomolecular NMR Spectroscopy. *Bioinformatics* **2014**, *31* (8), 1325–1327. https://doi.org/10.1093/BIOINFORMATICS/BTU830.

(6) Krieger, E.; Koraimann, G.; Vriend, G. Increasing the Precision of Comparative Models with YASARA NOVA—a Self-Parameterizing Force Field. *Proteins: Structure, Function, and Bioinformatics* **2002**, *47* (3), 393–402. https://doi.org/10.1002/PROT.10104.

(7) Krieger, E.; Vriend, G. YASARA View—Molecular Graphics for All Devices—from Smartphones to Workstations. *Bioinformatics* **2014**, *30* (20), 2981–2982. https://doi.org/10.1093/BIOINFORMATICS/BTU426.

(8) Doshi, U.; Hamelberg, D. Reoptimization of the AMBER Force Field Parameters for Peptide Bond (Omega) Torsions Using Accelerated Molecular Dynamics. *Journal of Physical Chemistry B* **2009**, *113* (52), 16590–16595. https://doi.org/10.1021/jp907388m.

(9) Wang, L.; Li, P.; Menche, D. Concise Synthesis of Tetrahydropyrans by a Tandem Oxa-Michael/Tsuji-Trost Reaction. *Angew Chem Int Ed Engl* **2010**, *49* (48), 9270–9273. https://doi.org/10.1002/ANIE.201003304.

(10) Zhang, H. J.; Yang, Z. P.; Gu, Q.; You, S. L. Tandem Pd-Catalyzed Intermolecular Allylic Alkylation/Allylic Dearomatization Reaction of Benzoylmethyl Pyridines, Pyrazines, and Quinolines. *Org Lett* **2019**, *21* (9), 3314–3318. https://doi.org/10.1021/ACS.ORGLETT.9B01060.

(11) Damez, C.; Labrosse, J. R.; Lhoste, P.; Sinou, D. An Easy Palladium-Catalyzed Access to Substituted 3-Methylene-3,4-Dihydro-2H-1,5-Benzodioxepines. *Synthesis (Stuttg)* **2001**, No. 10. https://doi.org/10.1055/s-2001-16092.

(12) Nishigaki, S.; Shibata, Y.; Tanaka, K. Rhodium-Catalyzed Chemo- and Regioselective Intermolecular Cross-Cyclotrimerization of Nonactivated Terminal and Internal Alkynes. *Journal of Organic Chemistry* **2017**, *82* (20). https://doi.org/10.1021/acs.joc.7b02121.

(13) Gole, B.; Sanyal, U.; Banerjee, R.; Mukherjee, P. S. High Loading of Pd Nanoparticles by Interior Functionalization of MOFs for Heterogeneous Catalysis. *Inorg Chem* **2016**, *55* (5). https://doi.org/10.1021/acs.inorgchem.5b02739.

(14) Einaru, S.; Shitamichi, K.; Nagano, T.; Matsumoto, A.; Asano, K.; Matsubara, S. Trans-Cyclooctenes as Halolactonization Catalysts. *Angew Chem Int Ed Engl* **2018**, *57* (42), 13863–13867. https://doi.org/10.1002/ANIE.201808320.

(15) Liu, J.; Yuan, F.; Ma, X.; Auphedeous, D. i. Y.; Zhao, C.; Liu, C.; Shen, C.; Feng, C. The Cooperative Effect of Both Molecular and Supramolecular Chirality on Cell Adhesion. *Angewandte Chemie International Edition* **2018**, *57* (22), 6475–6479. https://doi.org/10.1002/ANIE.201801462.

(16) Einaru, S.; Shitamichi, K.; Nagano, T.; Matsumoto, A.; Asano, K.; Matsubara, S. Trans-Cyclooctenes as Halolactonization Catalysts. *Angewandte Chemie International Edition* **2018**, *57* (42), 13863–13867. https://doi.org/10.1002/ANIE.201808320.

(17) Fukuda, Y.; Seto, S.; Furuta, H.; Ebisu, H.; Oomori, Y.; Terashima, S. Novel Seco Cyclopropa[c]Pyrrolo[3,2-e]Indole Bisalkylators Bearing a 3,3’-Arylenebisacryloyl Group as a Linker. *J Med Chem* **2001**, *44* (9), 1396–1406. https://doi.org/10.1021/JM000107X.

(18) Hirata, G.; Yamada, N.; Sanada, S.; Onodera, G.; Kimura, M. Palladium-Catalyzed [4 + 2] Cycloaddition of Aldimines and 1,4-Dipolar Equivalents via Amphiphilic Allylation. *Org Lett* **2015**, *17* (3), 600–603. https://doi.org/10.1021/OL503614D.
